# Catalytic Thioglycoside Activation with Diazo-Derived Copper Carbenes

**DOI:** 10.3390/molecules29225367

**Published:** 2024-11-14

**Authors:** Surya Pratap Singh, Umesh Chaudhary, Indrajeet Sharma

**Affiliations:** Department of Chemistry and Biochemistry, University of Oklahoma, 101 Stephenson Parkway, Norman, OK 73019-5251, USA; surya.pratap@ou.edu (S.P.S.); umesh.c@ou.edu (U.C.)

**Keywords:** earth-abundant metals, copper catalysis, thioglycoside donor, orthogonal reactivity, iterative synthesis, intramolecular activation

## Abstract

Traditional glycosylation methods using thioglycosides often require harsh conditions or expensive metal catalysts. This study presents a more sustainable alternative by employing copper, an earth-abundant catalyst. We developed diazo-based thioglycoside donors that, through copper catalysis, undergo intramolecular activation to form glycosyl sulfonium ions, leading to the generation of oxocarbenium ions. This versatile approach efficiently accommodates a variety of *O*-nucleophiles, including primary, secondary, and tertiary, as well as complex bioactive molecules. It is compatible with various glycosyl donors and protecting groups, including superarmed, armed, and disarmed systems. Notably, the methodology operates orthogonally to traditional thioglycoside and alkyne donors and has been successfully applied to the orthogonal iterative synthesis of trisaccharides. Mechanistic insights were gained by studying the electronic effects of electron-donating (OMe) and electron-withdrawing (NO_2_) groups on the donors, offering a valuable understanding of the intramolecular reaction pathway.

## 1. Introduction

Carbohydrates are essential biological molecules in many physiological processes, including cell–cell recognition, signaling, and immune responses [1,2]. The synthesis of complex carbohydrates and glycoconjugates is crucial in understanding these processes and developing therapeutic agents [3]. Glycosylation, the process of attaching sugar moieties to other molecules, is critical in synthesizing these intricate structures [2,3]. Among various glycosyl donors, thioglycosides have gained prominence due to their stability and ease of handling, making them ideal candidates for glycosylation reactions [4,5].

**Figure 1:**
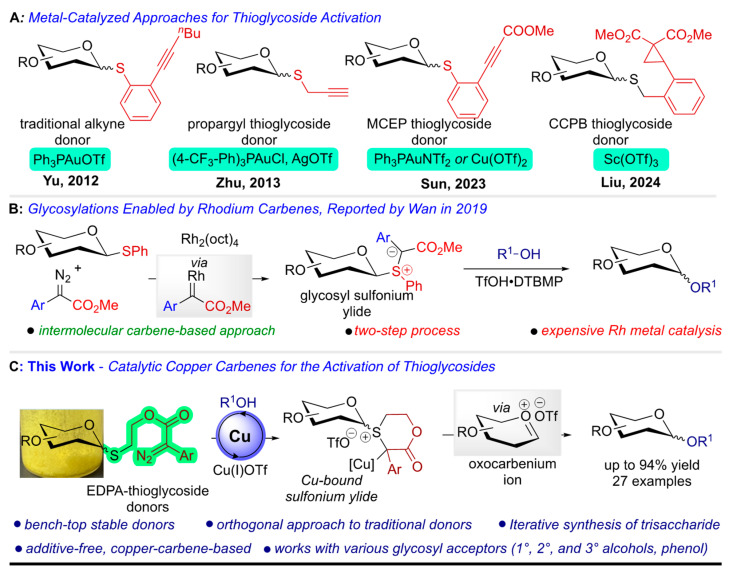
Catalytic activation of thioglycosides and our work. (**A**) Previous approaches for the catalytic thioglycoside activation [13,14,15,16]. (**B**) Catalytic thioglycoside activation with rhodium carbenes [21]. (**C**) Copper-carbene mediated catalytic activation of thioglycoside (this work).

The activation of thioglycoside donors is a crucial aspect of glycosylation chemistry. Typically, this activation involves promoters like *N*-iodosuccinimide (NIS) [6,7], tri-chloroisocyanuric acid (TCICA) [8], silver salts [9], and TMSOTf [10], which generate a reactive glycosyl oxocarbenium ion to facilitate glycosidic bond formation. Other methods have been recently reported, utilizing triperoxide/TMSOTf [11], electrophilic nitrogen mesitylenesulfonyl-hydroxylamine (MSH), and in situ benzyne for glycosylation [12]. While effective, these traditional and recent methods often require harsh conditions and stoichiometric amounts of promoters, limiting their applicability and functional group compatibility. Activating thioglycoside donors under catalytic conditions has been challenging for synthetic organic chemists. In 2012, Yu and coworkers first reported on the gold (Au(I))-catalyzed activation of well-established alkyne-based glycosyl donors [13]. Shortly after, in 2013, Adhikari et al. activated propargyl thioglycosides using Au–Ag dual-metal catalysts [14]. Recently, Sun and coworkers achieved Au- and Cu-catalyzed activation of ortho-methoxycarbonyl ethynyl phenyl thioglycosides [15], and very recently, Liu and coworkers harnessed the potential of the strained cyclopropyl ring in glycosylation activation using catalytic Sc(OTf)_3_ (Figure 1A) [16,17]. Apart from these reports, few known reports perform superacid-catalyzed activation of thioglycosides [18,19,20]. In 2019, the Wan group reported the catalytic activation of thioglycosides using rhodium carbenes [21]. They demonstrated the formation of glycosyl sulfonium ylide upon the reaction of thioglycoside with rhodium carbene, followed by Brønsted acid-derived cascade formation of a glycosyl sulfonium ion, which further transforms into a glycosyl oxocarbenium ion, leading to glycosidic linkages (Figure 1B).

There has been a remarkable development in the catalytic activation of thioglycoside, but it has noticeable shortcomings. More specifically, expensive metals, including Au, Ag, Rh, Sc, and harsh reaction conditions are used. To overcome these limitations, a methodology for activating thioglycosides using earth-abundant metal catalysts, which can proceed through mild reaction conditions and offer the orthogonal reactivity needed to establish traditional donors, must be developed.

We aim to develop a copper-catalyzed, carbene-based method using earth-abundant copper to activate thioglycosides, addressing the current challenges in this area. Our hypothesis is centered on the intramolecular activation of thioglycosides through copper carbenes (Figure 1C). The designed donor can be easily synthesized in a single step by coupling well-established anomeric thiols with linear donor–acceptor diazo linkers. This approach will introduce orthogonal reactivity compared to traditional thioglycoside and alkyne donors. We plan to screen a variety of nucleophiles, including primary, secondary, and tertiary alcohols, phenols, and biologically relevant molecules like cholesterol. Additionally, iterative oligosaccharide synthesis will be conducted to explore the broader applications of this methodology.

## 2. Results and Discussion

**Scheme 1:**
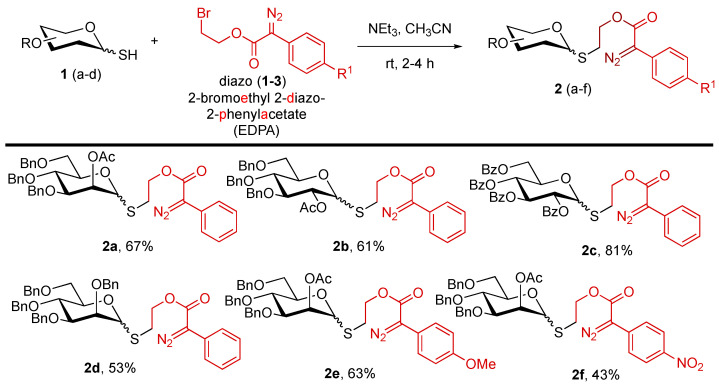
Synthesis of novel EDPA thioglycoside donors ^a^. ^a^ Reactions were performed with glycosyl thiol (1.0 equiv.), diazo linker 1-3 (1.2 equiv.), NEt_3_ (1.5 equiv.), and CH_3_CN (0.5 M). EDPA = 2-bromoethyl 2-diazo-2-phenylacetate.

We initiated our studies by synthesizing a designed thioglycoside donor. To access these donors, we coupled benzylated/benzoylated glycosyl thiols [22], which were used without purification. The crude thiols were coupled with a 2-bromomethyl 2-diazo-2-phenylacetate (EDPA) linker [23] using triethylamine as a base and acetonitrile as a solvent (Scheme 1) [16]. These donors were yellow, solid, and benchtop stable, showcasing the ease of practical utility. Using the same strategy, we synthesized both superarmed (2-*O*-Ac-3,4,6-tri-*O*-Bn) and disarmed (perbenzoylated) protecting groups with different classes of carbohydrates (glucose and mannose). Overall, synthesizing newly designed donors was simple and could be applied to various sugars.

In our efforts to optimize the reaction conditions, we chose 2-*O*-Ac-3,4,6-tri-*O*-Bn mannose (superarmed) EDPA thioglycoside **2a** as our model donor and methyl 6-OH, 2,3,4-tri-*O*-Bn-glucopyranoside **3a** as the model glycosyl acceptor. In our first attempt, Cu(I)OTf was used as a catalyst, and 2-ethyl 2-diazo-2-phenylacetate thioglycoside (EDPAT) donor **2a** was coupled with a glycosyl acceptor using CH_2_Cl_2_ as a solvent. To our delight, after 5 h, both starting materials were consumed, and the desired product was observed at a 68% yield (entry 1, Table 1). After well-crafted initial success, we turned our attention to screening other metal catalysts. In continuation of that, we screened Fe(BF_4_)_2_·6H_2_O (entry 2), ZnCl_2_ (entry 3), Zn(OTf)_2_ (entry 4), and Rh_2_OAc_4_ (entry 5). None of these catalysts were observed to produce the desired disaccharide in significant amounts. These screenings indicated that copper, along with the triflate counter-anion, is the best metal to provide a good yield of the desired product. To probe the role of the counter-anion, we tested other counter-anions, such as PF_6_ (entry 6), BF_4_ (entry 7), and I (entry 8); still, the triflate anion was the most optimal, further showcasing the importance of OTf in carbohydrate chemistry [24]. Our studies also found that Cu(OTf)_2_ was equally efficient in catalyzing the reaction (entry 9). Apart from the metal catalysts, we also screened strong Brønsted acids (TfOH), which are known to decompose donor-acceptor diazos. The diazo remained unreacted, and TfOH was quenched by molecular sieves (entry 10), indicating no potential TfOH generated in situ from the metal triflates responsible for the transformation. The reaction was performed at 0 °C, which further led to a slight increase in the yield but with a prolonged reaction time (entry 11). Increasing the amount of donor to 1.5 equivalent produces slightly higher yields (entry 12), while performing the reaction at room temperature produces a similar yield but with a lower reaction time (entry 13). We further reduced the catalyst loading (10 mol%), resulting in the exact yield as the desired product (entry 14). We further attempted to increase the yield and charged donors in portions. Adding donors in portions led to a significant increase in the product (entry 15). The portion-wise addition of Cu(OTf)_2_ also led to a considerable increment in the product yield (entry 16). We also screened various solvents, such as CH_3_CN (entry17), THF (entry 18), toluene (entry 19), and EtOAc (entry 20). Toluene provided better yields from these solvents, but CH_2_Cl_2_ was still the most optimal solvent.

In the end, we performed a reaction without the catalyst to further probe the role of the copper catalyst, leading to no decomposition of the donor. Finally, Cu(I)OTf in 10 mol% was the optimum catalyst, and the portion-wise addition of the donor was the most suitable condition for the best results.

**Scheme 2:**
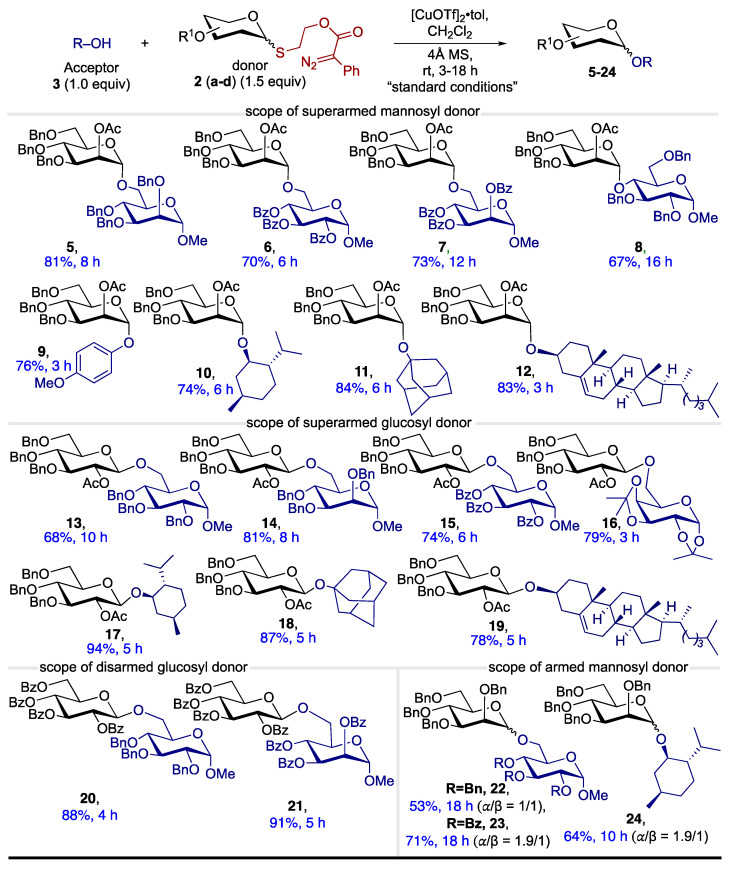
Substrate scope of our novel EDPAT donors ^a^. ^a^ Reactions were conducted with an acceptor (0.050 mmol), donor (0.075 mmol), promoters (0.005 mmol, 10 mol%), and CH_2_Cl_2_ (0.05 M).

After optimizing the reaction conditions with superarmed donor **2a**, we sought to broaden the scope of glycosyl donors and acceptors. We initially explored a range of primary glycosyl acceptors, including benzyl mannopyranoside and benzoyl-protected gluco- and mannopyranosides, coupling them with EDPAT donor **2a**. These acceptors all provided excellent yields of the desired disaccharides (**5**–**7**, Scheme 2).

Next, we tested a 2°-glycosyl acceptor, which also delivered a good yield of disaccharide **8**, although requiring a longer reaction time for completion. With these successful glycosyl acceptor screenings, we turned our attention to other nucleophiles. As a nucleophile, phenol gave a good yield of the desired product **9**. We also tested a secondary alcohol (*l*-menthol) and tertiary alcohol (1-adamantanol), both of which produced the desired products (**10** and **11**) in excellent yields. Furthermore, we demonstrated the tolerance of donor 2a by glycosylating the bioactive molecule cholesterol **12**, again achieving excellent yields. The success of mannosyl-superarmed EDPAT donor **2a** with a broad range of *O*-nucleophiles motivated us to investigate other glycosyl donors.

Next, we screened glucosyl EDPAT donor **2b** with various glycosyl acceptors, including benzyl-protected glucose and mannose, benzoyl-protected glucose, and acetonide-protected galactose. All these acceptors reacted well with donor **2b**, yielding the desired disaccharides (**13**–**16**) in excellent yields. Similarly, 2°- and 3°-nucleophiles such as chiral menthol, adamantanol, and bioactive cholesterol provided the expected products (**17**–**19**) with good yields. After successfully testing the superarmed donors (**2a**, **2b**) with a wide range of nucleophiles, we focused on screening a disarmed glycosyl donor. Using our synthesized glucosyl, EDPAT disarmed donor **2c**, we coupled it with various glycosyl acceptors, achieving excellent results and yielding the desired products (**20**, **21**). Finally, we also screened the armed protection group (benzyl) with mannosyl EDPAT donor **2d**, which successfully coupled with glycosyl acceptors, including menthol, yielding good products (**22**–**24**) and a mixture of diastereomers. Here, we observed an interesting reactivity pattern, where disarmed donors provided the disaccharides in less reaction time as compared to the armed donors. These experimental observations can be explained by neighboring group participation (NGP) in disarmed donor 2c and superarmed donor 2b. Both donors have similar reactivity and reaction times under our optimized conditions. More specifically, the benzoyl group stabilizes the oxocarbenium ion, leading to the efficient formation of glycosidic bonds. In the case of an armed glycosyl donor, there is no NGP to stabilize the oxocarbenium ion, which could be the reason for the low yields and prolonged reaction time. Overall, our methodology demonstrates robust compatibility with various glycosyl EDPAT donors and acceptors, highlighting its versatility. This approach offers a compelling catalytic glycosylation strategy compared to other precious metal-catalyzed thioglycoside activation methods.

**Figure 2:**
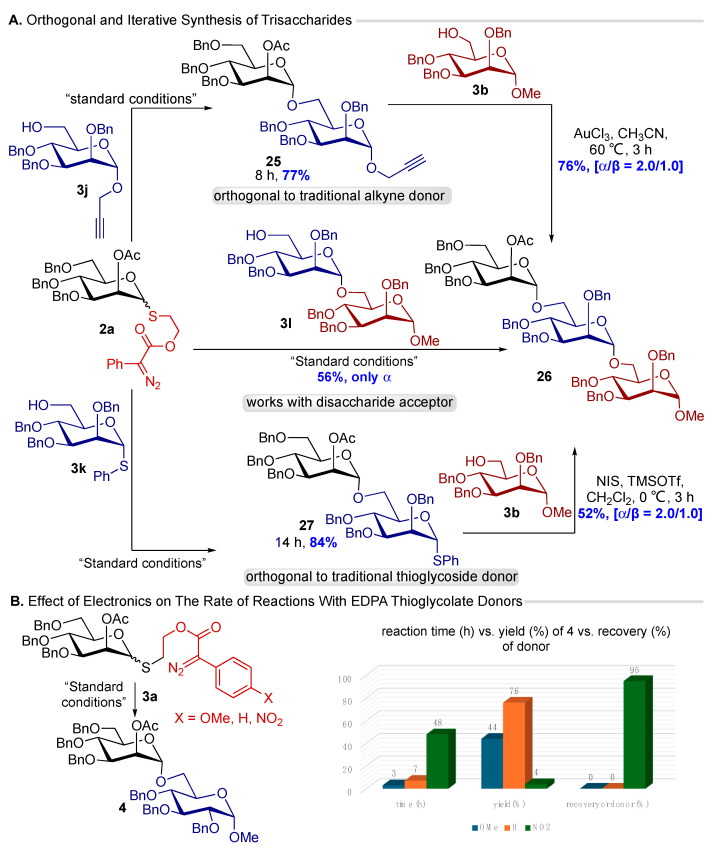
Applications of our EDPAT donor and control experiment. (**A**) Orthogonal and iterative synthesis of trisaccharide. (**B**) Effect of electronics on the rate and yield of the reaction.

After successfully screening a myriad of glycosyl donors and acceptors, we focused on accessing the oligosaccharides utilizing the orthogonal reactivity of our EDPAT donors. We synthesize literature-known acceptors bearing traditional alkyne **3j** and thioglycoside **3k** functional groups at the anomeric position. We coupled these acceptors with EDPAT donor **2a** under our optimized conditions (Figure 2), and ecstatically, both couplings work with excellent yields of the disaccharides (**25**, **27**), further providing evidence for the orthogonal reactivity. Furthermore, these disaccharides were activated under well-established reaction conditions (Figure 2) to access the trisaccharide **26**. After this orthogonal iterative synthesis of trisaccharide, we also synthesized disaccharide acceptor **3l** and coupled it with EDPAT donor **2a** to afford the same trisaccharide **26** (Figure 2). Overall, these studies further supported our claim that this methodology is one of the best approaches for the catalytic activation of thioglycosides for oligosaccharide synthesis.

In the end, we also synthesized EDPAT donors having different electronic functional groups OMe (**2e**) [25] and NO_2_ (**2f**) at the para position of the phenyl ring of diazo functionality. The coupling of these three donors (**2a**, **2e**, and **2f**) with the glycosyl acceptor **3a** demonstrates the effect of electronics. Specifically, donor **2e** decomposes rapidly, only providing 44% yield of the desired product, while **2f** was found to be less reactive under these conditions, providing the recovered yield of unreacted donors in 95%. The diazo compounds used in this study are donor-acceptor diazos. Their stability is enhanced by adding electron-withdrawing groups, while electron-donating groups destabilize the diazo compounds. An electron-donating group at the para position of the phenyl ring makes the diazo less stable. In contrast, an electron-withdrawing group (NO_2_) at the para position of the phenyl ring further stabilizes the diazo, making it hard to decompose to carbene under mild Cu-catalytic conditions. Ultimately, the OMe group will form copper carbenoid very quickly, increasing the concentration of highly reactive carbene in the reaction medium. This further leads to extensive side reactions. Overall, having no substitution at the para positions (parent donor **2a**) remains the best option to achieve the desired reactivity. These studies provide us with insights into the reaction pathway.

**Figure 3:**
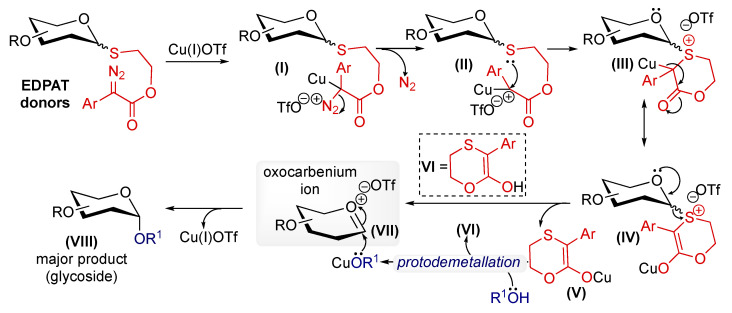
Plausible reaction mechanism for activating EDPAT donors using Cu(I)OTf for stereoselective glycosylation.

After completing the experiments, we proposed plausible reaction pathways for this transformation (Figure 3). The reaction begins with the formation of copper-bound diazo complex **I**, which provides the copper carbenoid **II** intermediate upon extrusion of the dinitrogen molecule. The copper carbenoid coordinates with the sulfur atom, bringing it near the in situ-generated carbene through chelation. Inserting the sulfur atom into copper carbene produces the copper-bound glycosyl sulfonium ion **III**. The copper-bound C-enolate then tautomerizes into the corresponding more stable metal-bound *O*-enolate **IV**, as described in our previous work [26]. This intermediate releases ((3-argio-5,6-dihydro-1,4-oxathiin-2-yl)oxy)copper as the possible byproduct **V** to generate the oxocarbenium ion **VII**, a reactive intermediate in glycosylation chemistry. Simultaneously, the byproduct **V** undergoes protodemetallation to generate copper-bound activated glycosyl acceptor (*O*-nucleophiles) and 3-argio-5,6-dihydro-1,4-oxathiin-2-ol as a plausible byproduct (**VI**), as described in our related work on copper carbene-mediated 1,2-cis-furanosylations [27]. Finally, the oxocarbenium ion reacts with the activated copper nucleophile to generate the major glycoside product **VIII**. The findings from all our studies support this proposed reaction pathway.

## 3. Experimental Section: Materials and Methods

### 3.1. General Considerations

Reagents: The reagents and solvents were obtained from Sigma-Aldrich (www.sigma-aldrich.com, St. Louis, MO, USA), Chem-Impex (www.chemimpex.com, Wood Dale, IL, USA), Acros Organics (www.fishersci.com, Geel, Belgium), or AmBeed (Arlington Heights, IL, USA) and used without further purification unless otherwise indicated. Dry solvents (acetonitrile) were obtained from Acros Organics (www.fishersci.com), and dichloromethane was distilled over CaH_2_ under N_2_ unless stated otherwise. THF purchased from Sigma-Aldrich was distilled over Na metal with a benzophenone indicator. Toluene was obtained from Sigma-Aldrich. Reactions: All reactions were performed in flame-dried glassware under positive N_2_ pressure with magnetic stirring unless otherwise noted. Liquid reagents and solutions were transferred through rubber septa via syringes flushed with N_2_ before use. Cold baths were generated as follows: 0 °C with wet ice/water and −78 °C using a Julabo chiller. Chromatography: TLC was performed on 0.25 mm E. Merck silica gel 60 F254 plates (Rahway, NJ, USA) and visualized under UV light (254 nm) or by staining with potassium permanganate (KMnO_4_), cerium ammonium molybdate (CAM), phosphomolybdic acid (PMA), and ninhydrin. Silica flash chromatography was performed on Sorbtech 230–400 mesh silica gel 60 (Norcross, Georgia). Analytical instrumentation: NMR spectra were recorded on a Varian VNMRS (Palo Alto, CA, USA) at 300, 400, 500, and 600 MHz and a Jeol 400 and 500 NMR spectrometer (Tokyo, Japan) in CDCl_3_ unless otherwise indicated. Chemical shifts are expressed in ppm relative to solvent signals: CDCl_3_ (^1^H, 7.26 ppm; ^13^C, 77.0 ppm); coupling constants are expressed in Hz. NMR spectra were processed using Mnova 14.2.1-27684 (www.mestrelab.com/software/mnova-nmr, accessed on 20 June 2023). Mass spectra were obtained at the OU Analytical Core Facility on an Agilent 6538 High-Mass-Resolution QTOF Mass Spectrometer (Santa Clara, CA, USA) and an Agilent 1290 UPLC. We utilized Kessil blue light lamps at four distinct wavelengths (nm)—PR160-390, PR160-440, PR160-456, and PR160-467—as the blue light source, accompanied by a cooling fan to regulate the ambient reaction temperature.

### 3.2. General Procedure A for the Synthesis of EDPA Thioglycoside Donor ***3*** (***a***–***f***)

To a solution of thiol, **1** (**a**–**d**) (1.0 equiv.) and a diazo linker (**1**–**3**) (1.15 equiv.) in anhydrous CH_3_CN (0.3 M) were added to triethylamine (1.5 equiv.). The mixture was stirred at room temperature for two hours before being concentrated in vacuo. The residue was purified using silica gel column chromatography (hexane:EtOAc = 5:1) to afford the desired EDPA thioglycoside donor **2** (**a**–**f**).

2-*O*-acetyl-3,4,6-tri-*O*-benzyl-α/β-d-mannopyranoside EDPA thioglycoside donor (**2a**): This was prepared according to general procedure A: a solution of 2-*O*-acetyl-3,4,6-tri-*O*-benzyl-d-mannopyranosyl thiol **1a** [28] (1 g, 1.98 mmol, 1.0 equiv.) was coupled with diazo linker-1 [23] (0.608 g, 2.26 mmol, 1.15 equiv.) to afford EDPA thioglycoside donor **2a** (0.93 g, 67%, α:β = 10:1). R*_f_* = 0.3 (hexane:ethyl acetate = 80:20). ^1^H NMR (400 MHz, CDCl_3_) δ 7.48 (d, *J* = 8.0 Hz, 2H), 7.39 (t, *J* = 7.8 Hz, 2H), 7.31 (dq, *J* = 11.5, 5.9 Hz, 13H), 7.22–7.15 (m, 3H), 5.46 (s, 1H), 5.37 (s, 1H), 4.86 (d, *J* = 10.7 Hz, 1H), 4.69 (dd, *J* = 11.6, 7.3 Hz, 2H), 4.51 (dd, *J* = 20.5, 11.3 Hz, 4H), 4.38 (dt, *J* = 11.4, 6.9 Hz, 1H), 4.17 (dd, *J* = 8.7, 3.2 Hz, 1H), 3.97–3.88 (m, 2H), 3.84 (dd, *J* = 10.5, 4.5 Hz, 1H), 3.71 (d, *J* = 10.7 Hz, 1H), 2.99 (dt, *J* = 13.8, 6.9 Hz, 1H), 2.88 (dt, *J* = 13.7, 6.5 Hz, 1H), 2.17 (s, 3H). ^13^C NMR (101 MHz, CDCl_3_) δ 170.44, 138.31, 138.18, 137.65, 129.07, 128.57, 128.46, 128.42, 128.30, 128.02, 127.99, 127.87, 127.80, 127.71, 126.01, 124.11, 83.31, 78.50, 75.35, 74.49, 73.52, 72.16, 72.05, 70.27, 68.77, 63.51, 30.37, 21.26. HRMS (ESI): calc. for C_39_H_40_N_2_NaO_8_S (M + Na): 719.2403; found: 719.2420.
2-*O*-acetyl-3,4,6-tri-*O*-benzyl-α/β-d-glucopyranoside EDPA thioglycoside donor (**2b**): This was prepared according to general procedure A: a solution of 2-*O*-acetyl-3,4,6-tri-*O*-benzyl-d-glucoopyranosyl thiol **1b** [28] (0.5 g, 0.99 mmol, 1.0 equiv.) was coupled with diazo linker-1 [23] (0.291 g, 1.13 mmol, 1.15 equiv.) to afford EDPA thioglycoside donor **2b** (0.415 g, 61%, α:β = 1:10). R*_f_* = 0.3 (hexane:ethyl acetate = 80:20). ^1^H NMR (400 MHz, CDCl_3_) δ 7.47 (d, *J* = 7.8 Hz, 2H), 7.39 (t, *J* = 7.7 Hz, 2H), 7.33–7.27 (m, 13H), 7.21–7.18 (m, 3H), 5.04 (t, *J* = 9.4 Hz, 1H), 4.81 (dd, *J* = 11.1, 9.1 Hz, 2H), 4.69 (d, *J* = 11.5 Hz, 1H), 4.59 (t, *J* = 11.7 Hz, 2H), 4.54 (d, *J* = 12.1 Hz, 1H), 4.48–4.40 (m, 3H), 3.77–3.66 (m, 5H), 3.52 (dq, *J* = 7.4, 2.2 Hz, 1H), 3.05 (ddd, *J* = 14.0, 7.7, 6.3 Hz, 1H), 2.88 (ddd, *J* = 13.9, 7.7, 6.2 Hz, 1H), 1.96 (s, 3H). ^13^C NMR (101 MHz, CDCl_3_) δ 169.64, 164.72, 138.13, 138.07, 137.86, 128.94, 128.43, 128.39, 128.34, 128.02, 127.88, 127.87, 127.80, 127.74, 127.70, 127.59, 125.87, 125.38, 124.02, 84.21, 83.54, 79.47, 77.74, 75.23, 75.11, 73.51, 73.49, 71.62, 68.81, 64.17, 28.59, 20.89. HRMS (ESI): calc. for C_39_H_40_N_2_NaO_8_S (M + Na): 719.2403; found: 719.2421.
2,3,4,6-tetra-*O*-benzoyl-α/β-d-glucopyranoside EDPA thioglycoside donor (**2c**): This was prepared according to general procedure A: a solution of 2,3,4,6-tetra-O-benzoyl-d-glucopyranosyl thiol **1c** [22] (0.25 g, 0.408 mmol, 1.0 equiv.) was coupled with diazo linker-1 [23] (0.132 g, 0.49 mmol, 1.15 equiv.) to afford EDPA thioglycoside donor **2c** (0.265 g, 81%, α:β = 1:8). R*_f_* = 0.2 (hexane:ethyl acetate = 80:20). ^1^H NMR (400 MHz, CDCl_3_) δ 8.02 (dd, *J* = 8.5, 1.4 Hz, 2H), 7.98–7.90 (m, 5H), 7.82 (dd, *J* = 8.5, 1.4 Hz, 2H), 7.44 (d, *J* = 1.4 Hz, 1H), 7.42 (d, *J* = 1.9 Hz, 2H), 7.40 (s, 1H), 7.39–7.35 (m, 8H), 7.29 (d, *J* = 8.2 Hz, 2H), 7.22–7.17 (m, 1H), 5.93 (t, *J* = 9.5 Hz, 1H), 5.68 (t, *J* = 9.8 Hz, 1H), 5.57 (t, *J* = 9.7 Hz, 1H), 4.92 (d, *J* = 10.1 Hz, 1H), 4.66 (dd, *J* = 12.2, 3.0 Hz, 1H), 4.52–4.46 (m, 2H), 4.44–4.35 (m, 1H), 4.19 (ddd, *J* = 9.0, 5.5, 2.9 Hz, 1H), 3.10 (ddd, *J* = 14.1, 8.0, 6.2 Hz, 1H), 2.94 (ddd, *J* = 14.0, 7.9, 6.1 Hz, 1H). ^13^C NMR (101 MHz, CDCl_3_) δ 166.16, 165.84, 165.31, 165.29, 133.62, 133.51, 133.40, 133.22, 130.09, 130.03, 129.96, 129.84, 129.81, 129.79, 129.60, 129.07, 129.02, 128.81, 128.77, 128.55, 128.52, 128.46, 128.42, 126.05, 125.36, 124.16, 84.27, 76.56, 73.99, 70.57, 69.50, 64.01, 63.22, 29.23. HRMS (ESI): calc. for C_44_H_36_N_2_NaO_11_S (M + Na): 823.1938; found: 823.1956.
2,3,4,6-tetra-*O*-benzyl-α/β-d-mannopyranoside EDPA thioglycoside donor (**2d**): This was prepared according to general procedure A: a solution of 2,3,4,6-tetra-*O*-benzyl-d-mannopyranosyl thiol **1d** [28] (0.25 g, 0.449 mmol, 1.0 equiv.) was coupled with diazo linker-1 [23] (0.139 g, 0.52 mmol, 1.15 equiv.) to afford EDPA thioglycoside donor **2d** (0.177 g, 53%, α:β=10:1). R*_f_* = 0.4 (hexane:ethyl acetate = 80:20). ^1^H NMR (400 MHz, CDCl_3_) δ 7.47 (dd, *J* = 8.6, 1.2 Hz, 2H), 7.41–7.36 (m, 4H), 7.34 (t, *J* = 2.0 Hz, 1H), 7.31 (dd, *J* = 6.9, 2.3 Hz, 7H), 7.29–7.27 (m, 7H), 7.21–7.15 (m, 3H), 5.44 (s, 1H), 4.88 (d, *J* = 10.7 Hz, 1H), 4.73 (d, *J* = 12.3 Hz, 1H), 4.67 (s, 1H), 4.64 (s, 1H), 4.55 (d, *J* = 4.3 Hz, 2H), 4.51 (dd, *J* = 12.1, 7.8 Hz, 4H), 4.35–4.28 (m, 1H), 4.14–4.09 (m, 1H), 4.05–3.99 (m, 1H), 3.84–3.79 (m, 3H), 3.72 (dd, *J* = 10.8, 2.0 Hz, 1H), 2.99–2.91 (m, 1H), 2.82 (dt, *J* = 13.7, 6.8 Hz, 1H). ^13^C NMR (101 MHz, CDCl_3_) δ 164.91, 138.46, 138.35, 138.23, 138.01, 129.08, 128.49, 128.43, 128.38, 128.07, 127.92, 127.84, 127.83, 127.79, 127.72, 127.59, 126.00, 125.46, 124.09, 82.42, 80.18, 76.07, 75.26, 74.98, 73.43, 72.31, 72.19, 72.04, 69.12, 63.23, 30.10. HRMS (ESI): calc. for C_44_H_44_N_2_NaO_7_S (M + Na): 767.2767; found: 767.2784.
2-*O*-acetyl-3,4,6-tri-*O*-benzyl-α/β-d-mannopyranoside 4-OMe-EDPA thioglycoside donor (**2e**): This was prepared according to general procedure A: a solution of 2-*O*-acetyl-3,4,6-tri-*O*-benzyl-d-mannopyranosyl thiol **1a** [28] (0.1 g, 0.197 mmol, 1.0 equiv.) was coupled with diazo linker-2 [25] (0.068 g, 0.226 mmol, 1.15 equiv.) to afford EDPA thioglycoside donor **2e** (0.087 g, 61%, α:β = 10:1). R*_f_* = 0.3 (hexane:ethyl acetate = 80:20). ^1^H NMR (400 MHz, CDCl_3_) δ 7.38–7.35 (m, 3H), 7.33–7.27 (m, 17H), 7.16 (dd, *J* = 7.2, 2.3 Hz, 3H), 6.95–6.92 (m, 2H), 5.45 (dd, *J* = 3.0, 1.7 Hz, 1H), 5.35 (d, *J* = 1.6 Hz, 1H), 4.85 (d, *J* = 10.7 Hz, 1H), 4.68 (dd, *J* = 11.6, 6.2 Hz, 3H), 4.52 (d, *J* = 11.1 Hz, 1H), 4.50–4.45 (m, 4H), 4.35 (dt, *J* = 11.3, 7.0 Hz, 1H), 3.93–3.89 (m, 2H), 3.80 (s, 3H), 3.70 (dd, *J* = 10.7, 2.1 Hz, 1H), 2.97 (dt, *J* = 13.8, 7.0 Hz, 1H), 2.90–2.82 (m, 1H), 2.16 (s, 3H). ^13^C NMR (101 MHz, CDCl_3_) δ 170.46, 158.21, 138.32, 138.19, 137.65, 128.57, 128.45, 128.42, 128.30, 128.28, 128.05, 128.01, 127.99, 127.96, 127.91, 127.88, 127.80, 127.72, 126.14, 116.78, 114.72, 114.37, 114.22, 114.16, 83.28, 78.49, 77.34, 75.34, 74.49, 73.57, 73.51, 72.14, 72.04, 70.27, 68.77, 63.48, 55.45, 55.41, 49.57, 30.35, 29.81, 21.23, 21.17, 14.30, 14.24.
2-*O*-acetyl-3,4,6-tri-*O*-benzyl-α/β-d-mannopyranoside 4-NO_2_-EDPA thioglycoside donor (**2f**): This was prepared according to general procedure A: a solution of 2-*O*-acetyl-3,4,6-tri-*O*-benzyl-d-mannopyranosyl thiol **1a** [28] (0.1 g, 0.196 mmol, 1.0 equiv.) was coupled with diazo linker-3 (0.071 g, 0.226 mmol, 1.15 equiv.) to afford EDPA thioglycoside donor **2f** (0.063 g, 43%, α:β = 10:1). R*_f_* = 0.2 (hexane:ethyl acetate = 80:20). ^1^H NMR (400 MHz, CDCl_3_) δ 8.22 (d, *J* = 9.1 Hz, 2H), 7.64 (d, *J* = 9.1 Hz, 2H), 7.31 (q, *J* = 7.9 Hz, 13H), 7.19–7.13 (m, 2H), 5.43 (s, 1H), 5.35 (s, 1H), 4.85 (d, *J* = 10.7 Hz, 1H), 4.70–4.62 (m, 2H), 4.53 (d, *J* = 11.1 Hz, 2H), 4.49 (d, *J* = 4.7 Hz, 1H), 4.46 (d, *J* = 3.2 Hz, 1H), 4.41 (dt, *J* = 11.3, 6.8 Hz, 1H), 4.15 (dd, *J* = 7.6, 4.5 Hz, 1H), 3.94–3.86 (m, 2H), 3.81 (dd, *J* = 10.7, 4.6 Hz, 1H), 3.72–3.68 (m, 1H), 2.99 (dt, *J* = 13.7, 6.8 Hz, 1H), 2.88 (dt, *J* = 13.6, 6.4 Hz, 1H), 2.17 (s, 3H). ^13^C NMR (101 MHz, CDCl_3_) δ 170.28, 163.25, 145.09, 138.13, 138.05, 137.49, 133.64, 128.43, 128.33, 128.27, 128.10, 127.88, 127.86, 127.70, 127.64, 127.57, 124.27, 123.19, 83.15, 78.32, 75.23, 74.43, 73.38, 72.14, 71.93, 70.17, 68.79, 63.82, 30.17, 21.05. HRMS (ESI): calc. for C_39_H_39_N_3_NaO_10_S (M + Na): 764.2254; found: 764.2269.

### 3.3. General Procedure B for the Activation of EDPA Thioglycosides for the Synthesis of Disaccharides (***4***–***27***)

In an oven-dried vial equipped with a stir bar, accepter 3 (**a**–**l**) (0.050 mmol) was combined with EDPA thioglycoside donor 2 (**a**–**f**) (0.075 mmol, 1.5 equiv.), 4 Å molecular sieves (50 mg or 100 wt.%), and [Cu(OTf)]_2_·tol (0.002 mmol, 0.1 equiv.) dissolved in CH_2_Cl_2_ (0.05 M). Upon complete addition, the reaction mixture was stirred at the ambient temperature, and the reaction progress was monitored using TLC and quenched with saturated aqueous NaHCO_3_ upon completion. The layers were separated, and the aqueous layer was washed with ethyl acetate. The organic layers were combined, dried over Na_2_SO_4_, filtered, and evaporated under reduced pressure. The crude product was dissolved in ethyl acetate and passed through a short silica pad to remove traces of iron (to check the crude material using ^1^H-NMR). The filtrate was evaporated, and the resulting crude material was purified using column chromatography.

**Note:** The donor was added in three portions initially; 0.8 equiv. was charged and TLC monitored during the reaction progress, and upon complete consumption of the donor, 0.35 equiv. was added into the reaction mixture twice.

Methyl 2-*O*-acetyl-3,4,6-tri-*O*-benzyl-α-d-mannopyranosyl-(1→6)-2,3,4-tri-*O*-benzyl-α-d-glucopyranoside (**4**): Following general procedure B, glycosyl acceptor **3a** (24.0 mg, 50.0 μmol) was coupled with EDPAT donor **2a** (54.0 mg, 75.0 μmol) to afford **4** (43.0 mg, 45.8 μmol, 88% yield) as a colorless viscous oil. R*_f_* = 0.3 (hexane:EtOAc = 70:30). ^1^H NMR (400 MHz, CDCl_3_) δ 7.38–7.35 (m, 2H), 7.34–7.30 (m, 10H), 7.29–7.26 (m, 10H), 7.25–7.23 (m, 3H), 7.20 (dd, *J* = 5.5, 2.1 Hz, 2H), 7.15 (dd, *J* = 7.5, 2.0 Hz, 1H), 7.13 (dd, *J* = 7.2, 2.1 Hz, 2H), 5.39 (td, *J* = 3.8, 1.9 Hz, 1H), 4.99 (d, *J* = 10.8 Hz, 1H), 4.90 (d, *J* = 1.9 Hz, 1H), 4.88–4.86 (m, 1H), 4.86–4.84 (m, 1H), 4.79 (d, *J* = 4.1 Hz, 1H), 4.78 (d, *J* = 5.3 Hz, 1H), 4.70–4.69 (m, 1H), 4.67 (s, 1H), 4.64–4.62 (m, 1H), 4.58 (d, *J* = 3.5 Hz, 1H), 4.53 (d, *J* = 6.0 Hz, 1H), 4.52–4.49 (m, 1H), 4.48 (s, 1H), 4.46 (d, *J* = 3.5 Hz, 1H), 4.43 (d, *J* = 8.6 Hz, 1H), 3.98 (t, *J* = 9.3 Hz, 1H), 3.91 (dd, *J* = 9.5, 3.2 Hz, 1H), 3.86 (t, *J* = 9.5 Hz, 1H), 3.81–3.78 (m, 1H), 3.73–3.70 (m, 3H), 3.66 (dd, *J* = 10.8, 4.2 Hz, 1H), 3.63 (dd, *J* = 11.4, 1.9 Hz, 1H), 3.54 (ddd, *J* = 13.3, 10.2, 2.8 Hz, 2H), 3.45–3.41 (m, 1H), 3.32 (s, 3H), 2.13 (s, 3H). ^13^C NMR (101 MHz, CDCl_3_) δ 170.31, 138.67, 138.50, 138.27, 138.20, 138.16, 138.09, 137.76, 128.45, 128.38, 128.34, 128.31, 128.24, 128.21, 128.15, 128.09, 128.07, 128.00, 127.92, 127.89, 127.86, 127.75, 127.72, 127.66, 127.63, 127.61, 127.59, 127.52, 127.48, 97.99, 97.78, 82.07, 80.07, 77.61, 77.58, 75.75, 75.12, 75.03, 74.90, 74.48, 74.17, 73.48, 73.32, 71.79, 71.57, 71.45, 71.38, 69.62, 68.90, 68.67, 68.48, 66.07, 55.08, 21.12. The data are identical to the literature report [29].
Methyl 2-*O*-acetyl-3,4,6-tri-*O*-benzyl-α-d-mannopyranosyl-(1→6)-2,3,4-tri-*O*-benzyl-α-d-mannopyranoside (**5**): Following general procedure B, glycosyl acceptor **3b** (24.0 mg, 50.0 μmol) was coupled with EDPAT donor **2a** (54.0 mg, 75.0 μmol) to afford **5** (39.0 mg, 41.5 μmol, 81% yield) as a colorless viscous oil. R*_f_* = 0.3 (hexane:EtOAc = 70:30). ^1^H NMR (400 MHz, CDCl_3_) δ 7.39–7.36 (m, 2H), 7.34–7.27 (m, 21H), 7.24–7.22 (m, 4H), 7.16–7.11 (m, 3H), 5.48–5.45 (m, 1H), 4.96 (d, *J* = 2.1 Hz, 1H), 4.91 (d, *J* = 11.2 Hz, 1H), 4.86 (dd, *J* = 10.9, 3.4 Hz, 1H), 4.73–4.70 (m, 3H), 4.67–4.65 (m, 1H), 4.64 (d, *J* = 3.9 Hz, 1H), 4.59 (d, *J* = 1.2 Hz, 2H), 4.55 (d, *J* = 6.9 Hz, 1H), 4.50 (d, *J* = 3.6 Hz, 1H), 4.48–4.46 (m, 2H), 4.44 (d, *J* = 4.3 Hz, 1H), 3.96 (dd, *J* = 9.2, 3.2 Hz, 1H), 3.91 (d, *J* = 9.4 Hz, 1H), 3.87 (t, *J* = 2.3 Hz, 2H), 3.80–3.77 (m, 2H), 3.72 (d, *J* = 5.2 Hz, 1H), 3.70 (t, *J* = 3.3 Hz, 1H), 3.67 (s, 1H), 3.59 (dd, *J* = 10.8, 1.9 Hz, 1H), 3.25 (s, 3H), 2.14 (s, 3H). ^13^C NMR (101 MHz, CDCl_3_) δ 170.28, 138.59, 138.45, 138.42, 138.30, 138.28, 137.82, 128.38, 128.35, 128.32, 128.30, 128.28, 128.23, 128.19, 128.06, 127.92, 127.86, 127.81, 127.76, 127.72, 127.62, 127.60, 127.53, 127.48, 127.43, 98.69, 97.94, 80.25, 77.56, 75.12, 74.99, 74.94, 74.59, 74.53, 74.47, 74.18, 73.48, 73.32, 72.58, 71.97, 71.79, 71.39, 71.35, 71.31, 70.90, 69.18, 68.90, 68.72, 68.52, 66.70, 54.64, 21.12. The data are identical to the literature report [30].
Methyl 2-*O*-acetyl-3,4,6-tri-*O*-benzyl-α-d-mannopyranosyl-(1→6)-2,3,4-tri-*O*-benzoyl-α-d-glucopyranoside (**6**): Following general procedure B, glycosyl acceptor **3c** (25.0 mg, 50.0 μmol) was coupled with EDPAT donor **2a** (52.0 mg, 75.0 μmol) to afford **6** (34.0 mg, 34.7 μmol, 70% yield) as a colorless viscous oil. R*_f_* = 0.2 (hexane:EtOAc = 70:30). ^1^H NMR (400 MHz, CDCl_3_) δ 7.98 (dd, *J* = 8.5, 1.3 Hz, 2H), 7.94 (dd, *J* = 8.5, 1.3 Hz, 2H), 7.87 (dd, *J* = 8.4, 1.4 Hz, 2H), 7.50 (ddt, *J* = 14.1, 7.1, 1.3 Hz, 2H), 7.46–7.41 (m, 2H), 7.41–7.35 (m, 3H), 7.33–7.27 (m, 15H), 7.16 (dd, *J* = 7.4, 2.1 Hz, 2H), 6.13 (t, *J* = 9.9 Hz, 1H), 5.56 (t, *J* = 9.9 Hz, 1H), 5.33 (dd, *J* = 3.3, 1.8 Hz, 1H), 5.26 (dd, *J* = 10.2, 3.7 Hz, 1H), 5.19 (d, *J* = 3.7 Hz, 1H), 4.87–4.83 (m, 2H), 4.60 (d, *J* = 2.2 Hz, 1H), 4.57 (s, 1H), 4.46 (d, *J* = 7.3 Hz, 1H), 4.43 (d, J = 7.7 Hz, 1H), 4.38 (d, *J* = 12.0 Hz, 1H), 4.20 (ddd, *J* = 10.2, 5.4, 3.2 Hz, 1H), 3.94 (dd, *J* = 9.3, 3.4 Hz, 1H), 3.91–3.86 (m, 1H), 3.84 (d, *J* = 9.5 Hz, 1H), 3.74–3.69 (m, 1H), 3.69–3.65 (m, 1H), 3.63 (dd, *J* = 11.6, 3.7 Hz, 1H), 3.53 (dd, *J* = 10.7, 1.9 Hz, 1H), 3.39 (s, 3H), 2.13 (s, 3H). ^13^C NMR (101 MHz, CDCl_3_) δ 170.30, 165.79, 165.15, 138.56, 138.18, 137.97, 133.31, 133.03, 129.92, 129.67, 129.24, 129.07, 128.94, 128.40, 128.38, 128.32, 128.25, 128.22, 128.12, 127.79, 127.71, 127.66, 127.50, 127.48, 97.88, 77.84, 77.21, 75.01, 74.15, 73.30, 72.09, 71.73, 71.45, 70.48, 69.64, 68.65, 68.60, 68.05, 66.30, 55.49, 21.09. The data are identical to the literature report [30].
Methyl 2-*O*-acetyl-3,4,6-tri-*O*-benzyl-α-d-mannopyranosyl-(1→6)-2,3,4-tri-*O*-benzoyl-α-d-mannopyranoside (**7**): Following general procedure B, glycosyl acceptor **3d** (25.0 mg, 50.0 μmol) was coupled with EDPAT donor **2a** (52.0 mg, 75.0 μmol) to afford **7** (36.0 mg, 36.7 μmol, 73% yield) as a colorless viscous oil. R*_f_* = 0.2 (hexane:EtOAc = 70:30). ^1^H NMR (400 MHz, CDCl_3_) δ 8.08 (d, *J* = 7.7 Hz, 2H), 7.96 (d, *J* = 7.9 Hz, 2H), 7.82 (d, *J* = 7.6 Hz, 2H), 7.62–7.51 (m, 1H), 7.44 (dt, *J* = 15.1, 7.8 Hz, 4H), 7.30 (dd, *J* = 19.6, 5.3 Hz, 17H), 7.19–7.09 (m, 2H), 5.97–5.76 (m, 2H), 5.65 (s, 1H), 5.34 (s, 1H), 4.91 (d, *J* = 16.7 Hz, 2H), 4.83 (d, *J* = 11.1 Hz, 1H), 4.65–4.29 (m, 6H), 4.27–4.17 (m, 1H), 4.00–3.81 (m, 3H), 3.71 (dd, *J* = 23.6, 11.8 Hz, 3H), 3.54 (d, *J* = 11.2 Hz, 1H), 3.45 (s, 3H), 2.12 (s, 3H). ^13^C NMR (101 MHz, CDCl_3_) δ 170.47, 165.66, 165.60, 165.53, 138.64, 138.24, 138.03, 133.64, 133.50, 133.25, 130.02, 129.99, 129.87, 129.48, 129.23, 129.14, 128.75, 128.59, 128.55, 128.48, 128.40, 128.38, 128.24, 128.18, 128.11, 128.03, 127.96, 127.92, 127.89, 127.82, 127.67, 98.63, 98.02, 78.25, 75.21, 74.21, 73.44, 71.76, 71.58, 70.63, 70.13, 69.18, 68.65, 68.60, 67.60, 66.83, 55.54, 21.27. The data are identical to the literature report [31].
Methyl 2-*O*-acetyl-3,4,6-tri-*O*-benzyl-α-d-mannopyranosyl-(1→4)-2,3,6-tri-*O*-benzyl-α-d-glucopyranoside (**8**): Following general procedure B, glycosyl acceptor **3e** (24.0 mg, 50.0 μmol) was coupled with EDPAT donor **2a** (54.0 mg, 75.0 μmol) to afford **8** (33.0 mg, 35.1 μmol, 67% yield) as a colorless viscous oil. R*_f_* = 0.3 (hexane:EtOAc = 70:30). ^1^H NMR (400 MHz, CDCl_3_) δ 7.38–7.34 (m, 1H), 7.33 (d, *J* = 1.9 Hz, 3H), 7.31–7.26 (m, 21H), 7.25–7.19 (m, 3H), 7.16–7.13 (m, 2H), 5.48–5.44 (m, 1H), 5.42 (d, *J* = 2.2 Hz, 1H), 5.04 (d, *J* = 11.2 Hz, 1H), 4.81 (d, *J* = 10.8 Hz, 1H), 4.76–4.72 (m, 1H), 4.67 (d, *J* = 18.7 Hz, 1H), 4.62 (d, *J* = 2.8 Hz, 1H), 4.59 (d, *J* = 2.3 Hz, 2H), 4.54 (d, *J* = 11.8 Hz, 2H), 4.48–4.41 (m, 3H), 4.41–4.34 (m, 2H), 3.93 (t, *J* = 9.1 Hz, 1H), 3.88–3.85 (m, 1H), 3.85–3.80 (m, 2H), 3.72 (s, 1H), 3.69 (t, *J* = 4.2 Hz, 2H), 3.67–3.63 (m, 1H), 3.54 (dd, *J* = 9.5, 3.5 Hz, 1H), 3.50 (dd, *J* = 10.8, 1.9 Hz, 1H), 3.38 (s, 3H), 1.98 (s, 3H). ^13^C NMR (101 MHz, CDCl_3_) δ 169.85, 138.52, 138.45, 138.20, 137.97, 137.92, 128.44, 128.30, 128.24, 128.20, 128.14, 128.05, 127.93, 127.88, 127.82, 127.61, 127.56, 127.53, 127.40, 127.27, 127.25, 99.30, 97.76, 81.74, 80.05, 78.20, 77.22, 75.71, 75.13, 75.09, 74.02, 73.44, 73.27, 72.41, 71.67, 69.57, 69.20, 68.72, 55.28, 20.95. The data are identical to the literature report [30].
*p*-Methoxyphenyl 2-*O*-acetyl-3,4,6-tri-*O*-benzyl-α-d-mannopyranoside (**9**): Following general procedure B, glycosyl acceptor **3f** (6.0 mg, 50.0 μmol) was coupled with EDPAT donor **2a** (54.0 mg, 75.0 μmol) to afford **9** (22.0 mg, 36.8 μmol, 76% yield) as a colorless viscous oil. R*_f_* = 0.5 (hexane:EtOAc = 70:30). ^1^H NMR (400 MHz, CDCl_3_) δ 7.38–7.27 (m, 13H), 7.18 (dd, *J* = 7.8, 1.7 Hz, 2H), 7.01–6.97 (m, 2H), 6.81–6.78 (m, 2H), 5.55 (dd, *J* = 3.4, 2.0 Hz, 1H), 5.46 (d, *J* = 2.0 Hz, 1H), 4.89 (d, *J* = 10.7 Hz, 1H), 4.77 (d, *J* = 11.1 Hz, 1H), 4.66 (d, *J* = 12.0 Hz, 1H), 4.62 (d, *J* = 11.1 Hz, 1H), 4.52 (d, *J* = 10.7 Hz, 1H), 4.46 (d, *J* = 12.0 Hz, 1H), 4.19 (dd, *J* = 9.0, 3.4 Hz, 1H), 4.00 (t, *J* = 9.5 Hz, 1H), 3.96 (ddd, *J* = 9.8, 4.1, 1.8 Hz, 1H), 3.82 (dd, *J* = 10.8, 4.1 Hz, 1H), 3.76 (s, 3H), 3.68 (dd, *J* = 10.8, 1.9 Hz, 1H), 2.18 (s, 3H). ^13^C NMR (101 MHz, CDCl_3_) δ 170.44, 155.10, 149.95, 138.31, 138.13, 137.87, 128.43, 128.32, 128.27, 128.09, 127.84, 127.80, 127.78, 127.65, 127.57, 117.81, 114.57, 96.86, 78.02, 75.22, 74.16, 73.35, 71.97, 71.86, 68.68, 68.65, 55.60, 21.12. The data are identical to the literature report [32].
*l*-Menthyl 2-*O*-acetyl-3,4,6-tri-*O*-benzyl-α-d-mannopyranoside (**10**): Following general procedure B, glycosyl acceptor **3g** (8.0 mg, 50.0 μmol) was coupled with EDPAT donor **2a** (54.0 mg, 75.0 μmol) to afford **10** (22.0 mg, 36.8 μmol, 74% yield) as a colorless viscous oil. R*_f_* = 0.6 (hexane:EtOAc = 70:30). ^1^H NMR (400 MHz, CDCl_3_) δ 7.39–7.26 (m, 13H), 7.18 (dd, *J* = 7.4, 2.1 Hz, 2H), 5.26 (dd, *J* = 3.3, 1.9 Hz, 1H), 4.94–4.86 (m, 2H), 4.71 (dd, *J* = 11.7, 6.6 Hz, 2H), 4.58 (d, *J* = 11.3 Hz, 1H), 4.50 (dd, *J* = 11.4, 10.0 Hz, 2H), 4.02–3.95 (m, 2H), 3.91–3.80 (m, 2H), 3.71 (dd, *J* = 10.6, 1.9 Hz, 1H), 3.35 (td, *J* = 10.6, 4.3 Hz, 1H), 2.16 (s, 3H), 2.05 (pd, *J* = 7.0, 2.5 Hz, 1H), 1.66–1.57 (m, 2H), 1.41–1.31 (m, 1H), 1.24–1.15 (m, 1H), 1.03–0.90 (m, 5H), 0.85 (d, *J* = 6.5 Hz, 3H), 0.78 (d, *J* = 7.0 Hz, 4H). ^13^C NMR (101 MHz, CDCl_3_) δ 170.58, 138.40, 138.31, 137.98, 128.37, 128.32, 128.25, 128.08, 127.92, 127.72, 127.70, 127.62, 127.50, 99.27, 81.63, 77.97, 75.19, 74.62, 73.36, 71.76, 71.41, 69.34, 69.05, 48.55, 42.71, 34.26, 31.58, 25.84, 23.30, 22.20, 21.20, 21.02, 16.31. The data are identical to the literature report [33].
Adamantanyl 2-*O*-acetyl-3,4,6-tri-*O*-benzyl-α-d-mannopyranoside (**11**): Following general procedure B, glycosyl acceptor **3h** (8.0 mg, 50.0 μmol) was coupled with EDPAT donor **2a** (54.0 mg, 75.0 μmol) to afford **11** (28.0 mg, 44.8 μmol, 84% yield) as a colorless viscous oil. R*_f_* = 0.7 (hexane:EtOAc = 70:30). ^1^H NMR (400 MHz, CDCl_3_) δ 7.37–7.26 (m, 12H), 7.17 (dd, *J* = 7.4, 2.1 Hz, 2H), 5.27 (d, *J* = 2.1 Hz, 1H), 5.18 (dd, *J* = 3.3, 2.0 Hz, 1H), 4.86 (d, *J* = 10.6 Hz, 1H), 4.73–4.68 (m, 2H), 4.55 (d, *J* = 11.1 Hz, 1H), 4.48 (dd, *J* = 11.3, 4.4 Hz, 2H), 4.08–3.99 (m, 2H), 3.90 (t, *J* = 9.6 Hz, 1H), 3.83 (dd, *J* = 10.6, 4.2 Hz, 1H), 3.68 (dd, *J* = 10.6, 2.0 Hz, 1H), 2.15 (s, 3H), 2.14–2.10 (m, 3H), 1.79 (d, *J* = 2.0 Hz, 6H), 1.64–1.55 (m, 6H). ^13^C NMR (101 MHz, CDCl_3_) δ 170.75, 138.46, 138.35, 138.11, 128.35, 128.30, 128.22, 128.06, 127.94, 127.69, 127.65, 127.59, 127.46, 90.95, 78.24, 75.20, 75.03, 74.68, 73.36, 71.71, 70.91, 70.44, 69.07, 42.28, 36.20, 30.59, 21.24. The data are identical to the literature report [33].
Cholesteryl 2-*O*-acetyl-3,4,6-tri-*O*-benzyl-α-d-mannopyranoside (**12**): Following general procedure B, glycosyl acceptor **3i** (19.0 mg, 50.0 μmol) was coupled with EDPAT donor **2a** (54.0 mg, 75.0 μmol) to afford **12** (34.0 mg, 40.8 μmol, 83% yield) as a colorless viscous oil. R*_f_* = 0.7 (hexane:EtOAc = 70:30). ^1^H NMR (400 MHz, CDCl_3_) δ 7.38–7.27 (m, 12H), 7.19–7.15 (m, 2H), 5.34 (dd, *J* = 3.4, 1.8 Hz, 1H), 5.29 (dd, *J* = 4.8, 2.7 Hz, 1H), 5.01 (d, *J* = 1.8 Hz, 1H), 4.86 (d, *J* = 10.6 Hz, 1H), 4.71 (dd, *J* = 11.5, 9.0 Hz, 2H), 4.55 (d, *J* = 11.1 Hz, 1H), 4.50 (d, *J* = 9.3 Hz, 1H), 4.48 (d, *J* = 8.0 Hz, 1H), 4.03 (dt, *J* = 6.1, 3.3 Hz, 1H), 3.92–3.89 (m, 2H), 3.85–3.81 (m, 1H), 3.72 (dd, *J* = 10.6, 1.3 Hz, 1H), 3.49 (dq, *J* = 10.9, 5.6 Hz, 1H), 2.35–2.27 (m, 2H), 2.15 (s, 3H), 2.05–1.97 (m, 2H), 1.95–1.81 (m, 4H), 1.61–1.42 (m, 8H), 1.41–1.30 (m, 4H), 1.29–1.24 (m, 2H), 1.13 (ddt, *J* = 15.7, 9.9, 5.6 Hz, 6H), 1.00 (s, 4H), 0.92 (d, *J* = 6.5 Hz, 3H), 0.88 (dd, *J* = 6.6, 1.8 Hz, 6H), 0.68 (s, 3H). ^13^C NMR (101 MHz, CDCl_3_) δ 170.58, 140.54, 138.37, 138.25, 138.06, 128.36, 128.32, 128.26, 128.03, 127.95, 127.78, 127.67, 127.63, 127.52, 121.92, 109.99, 95.81, 78.35, 77.22, 75.24, 74.49, 73.38, 71.76, 71.32, 69.31, 68.93, 56.75, 56.14, 50.07, 42.31, 39.84, 39.75, 39.52, 36.96, 36.67, 36.19, 35.78, 31.93, 31.87, 28.23, 28.01, 27.68, 24.29, 23.82, 22.82, 22.57, 21.18, 21.04, 19.33, 18.72, 11.86. The data are identical to the literature report [33].
Methyl 2-*O*-acetyl-3,4,6-tri-*O*-benzyl-β-d-glucopyranosyl-(1→6)-2,3,4-tri-*O*-benzyl-α-d-glucopyranoside (**13**): Following general procedure B, glycosyl acceptor **3a** (24.0 mg, 50.0 μmol) was coupled with EDPAT donor **2b** (54.0 mg, 75.0 μmol) to afford **13** (33.0 mg, 35.2 μmol, 68% yield) as a colorless viscous oil. R*_f_* = 0.3 (hexane:EtOAc = 70:30). ^1^H NMR (400 MHz, CDCl_3_) δ 7.35 (dd, *J* = 8.2, 1.6 Hz, 2H), 7.33–7.29 (m, 16H), 7.29–7.25 (m, 9H), 7.24 (s, 1H), 7.18 (dd, *J* = 7.4, 2.1 Hz, 2H), 5.05 (t, *J* = 8.6 Hz, 1H), 4.97 (d, *J* = 11.1 Hz, 1H), 4.83 (d, *J* = 10.8 Hz, 1H), 4.80–4.76 (m, 4H), 4.65 (dd, *J* = 11.8, 5.1 Hz, 2H), 4.59–4.55 (m, 3H), 4.55–4.51 (m, 2H), 4.39 (d, *J* = 8.1 Hz, 1H), 4.09 (dd, *J* = 10.8, 2.0 Hz, 1H), 3.97 (t, *J* = 9.3 Hz, 1H), 3.78–3.74 (m, 1H), 3.73 (dd, *J* = 11.1, 2.1 Hz, 1H), 3.69–3.61 (m, 4H), 3.52 (dd, *J* = 9.6, 3.6 Hz, 1H), 3.50–3.47 (m, 1H), 3.44 (t, *J* = 9.5 Hz, 1H), 3.35 (s, 3H), 1.87 (s, 3H). ^13^C NMR (101 MHz, CDCl_3_) δ 169.17, 138.83, 138.25, 138.14, 138.09, 137.81, 128.44, 128.42, 128.33, 128.15, 128.01, 127.89, 127.86, 127.83, 127.73, 127.72, 127.65, 127.56, 127.51, 100.95, 98.00, 79.82, 78.03, 77.74, 75.66, 75.36, 75.04, 75.00, 74.86, 73.43, 73.41, 72.93, 69.70, 68.81, 67.90, 55.06, 20.93. The data are identical to the literature report [33].
Methyl 2-*O*-acetyl-3,4,6-tri-*O*-benzyl-β-d-glucopyranosyl-(1→6)-2,3,4-tri-*O*-benzyl-α-d-mannopyranoside (**14**): Following general procedure B, glycosyl acceptor **3b** (24.0 mg, 50.0 μmol) was coupled with EDPAT donor **2b** (54.0 mg, 75.0 μmol) to afford **14** (39.0 mg, 41.6 μmol, 81% yield) as a colorless viscous oil. R*_f_* = 0.3 (hexane:EtOAc = 70:30). ^1^H NMR (400 MHz, CDCl_3_) δ 7.36 (dd, *J* = 8.3, 1.9 Hz, 2H), 7.34–7.27 (m, 26H), 7.18 (dd, *J* = 7.3, 2.2 Hz, 2H), 5.04 (dd, *J* = 9.5, 7.9 Hz, 1H), 4.91 (d, *J* = 11.2 Hz, 1H), 4.78 (d, *J* = 11.7 Hz, 2H), 4.72 (s, 2H), 4.68 (s, 2H), 4.65–4.63 (m, 1H), 4.61 (s, 1H), 4.58 (s, 3H), 4.55 (s, 1H), 4.51 (d, *J* = 7.3 Hz, 1H), 4.44 (t, *J* = 8.6 Hz, 2H), 4.18–4.14 (m, 1H), 3.88–3.85 (m, 1H), 3.77 (dd, *J* = 3.1, 1.9 Hz, 1H), 3.75–3.71 (m, 3H), 3.68 (d, *J* = 3.8 Hz, 1H), 3.66 (t, J = 2.2 Hz, 1H), 3.63 (s, 1H), 3.29 (s, 3H), 1.92 (s, 3H). ^13^C NMR (101 MHz, CDCl_3_) δ 169.31, 138.43, 138.25, 138.17, 137.97, 128.39, 128.38, 128.34, 128.32, 127.97, 127.89, 127.82, 127.79, 127.75, 127.69, 127.67, 127.64, 127.57, 127.53, 101.31, 98.83, 82.95, 80.29, 78.00, 77.21, 75.21, 75.04, 74.98, 74.94, 74.81, 74.45, 73.49, 73.10, 72.72, 72.07, 71.51, 68.91, 68.69, 54.58, 20.91. The data are identical to the literature report [34].
Methyl 2-*O*-acetyl-3,4,6-tri-*O*-benzyl-β-d-glucopyranosyl-(1→6)-2,3,4-tri-*O*-benzoyl-α-d-glucopyranoside (**15**): Following general procedure B, glycosyl acceptor **3c** (25.0 mg, 50.0 μmol) was coupled with EDPAT donor **2b** (52.0 mg, 75.0 μmol) to afford **15** (36.0 mg, 36.7 μmol, 74% yield) as a colorless viscous oil. R*_f_* = 0.2 (hexane:EtOAc = 70:30). ^1^H NMR (400 MHz, CDCl_3_) δ 7.99–7.97 (m, 2H), 7.94–7.92 (m, 2H), 7.85 (dd, *J* = 8.5, 1.3 Hz, 2H), 7.51 (td, *J* = 7.3, 1.4 Hz, 2H), 7.42 (tt, *J* = 7.2, 1.4 Hz, 1H), 7.38 (d, *J* = 8.5 Hz, 2H), 7.37–7.35 (m, 2H), 7.33 (dt, *J* = 8.5, 1.4 Hz, 1H), 7.30 (dd, *J* = 10.7, 1.2 Hz, 3H), 7.29–7.26 (m, 10H), 7.24 (dd, *J* = 4.9, 3.7 Hz, 1H), 7.18–7.16 (m, 2H), 6.14 (t, *J* = 9.7 Hz, 1H), 5.42 (t, *J* = 9.9 Hz, 1H), 5.23 (dd, *J* = 10.1, 3.7 Hz, 1H), 5.20 (d, *J* = 3.7 Hz, 1H), 5.06–5.03 (m, 1H), 4.79 (dd, *J* = 11.1, 7.2 Hz, 2H), 4.69 (d, *J* = 11.4 Hz, 1H), 4.55–4.53 (m, 2H), 4.47–4.42 (m, 2H), 4.27 (ddd, *J* = 9.2, 7.2, 1.9 Hz, 1H), 4.07 (dd, *J* = 10.9, 2.0 Hz, 1H), 3.70–3.67 (m, 4H), 3.66–3.64 (m, 1H), 3.45 (s, 3H), 2.02 (s, 3H). ^13^C NMR (101 MHz, CDCl_3_) δ 169.50, 165.81, 165.69, 165.37, 138.17, 138.01, 137.87, 133.40, 133.31, 133.01, 129.91, 129.85, 129.63, 129.26, 129.08, 128.87, 128.41, 128.38, 128.31, 128.22, 127.98, 127.83, 127.80, 127.71, 127.69, 127.55, 101.35, 96.64, 82.86, 77.81, 75.20, 75.03, 75.01, 73.41, 73.07, 72.14, 70.56, 69.53, 68.70, 68.39, 55.33, 20.95. The data are identical to the literature report [33].
Methyl 2-*O*-acetyl-3,4,6-tri-*O*-benzyl-β-d-glucopyranosyl-(1→6)-1,2:3,4-di-*O*-isopropylidene-α-d-galactopyranoside (**16**): Following general procedure B, glycosyl acceptor **3j** (13.0 mg, 50.0 μmol) was coupled with EDPAT donor **2b** (53.0 mg, 75.0 μmol) to afford **16** (29.0 mg, 39.5 μmol, 79% yield) as a colorless viscous oil. R*_f_* = 0.4 (hexane:EtOAc = 70:30). ^1^H NMR (400 MHz, CDCl_3_) (600 MHz, cdcl3) δ 7.35–7.31 (m, 4H), 7.31 (d, *J* = 2.3 Hz, 1H), 7.29 (d, *J* = 2.8 Hz, 2H), 7.27 (d, *J* = 12.1 Hz, 6H), 7.18 (dd, *J* = 7.7, 2.0 Hz, 2H), 5.49 (d, *J* = 5.0 Hz, 1H), 5.02–4.98 (m, 1H), 4.78 (dd, *J* = 11.1, 8.1 Hz, 2H), 4.68 (d, *J* = 11.4 Hz, 1H), 4.63 (d, *J* = 12.1 Hz, 1H), 4.58–4.55 (m, 2H), 4.54 (d, *J* = 6.4 Hz, 1H), 4.44 (d, *J* = 8.1 Hz, 1H), 4.27 (dd, *J* = 4.9, 2.4 Hz, 1H), 4.18 (dd, *J* = 8.1, 1.9 Hz, 1H), 4.06 (dd, *J* = 11.3, 3.5 Hz, 1H), 3.92 (ddt, *J* = 5.4, 3.5, 1.9 Hz, 1H), 3.73 (d, *J* = 2.9 Hz, 2H), 3.71–3.65 (m, 2H), 3.62 (dd, *J* = 11.3, 7.4 Hz, 1H), 3.47 (dt, *J* = 9.4, 3.2 Hz, 1H), 2.01 (s, 3H), 1.50 (s, 3H), 1.42 (s, 3H), 1.31 (s, 3H), 1.30 (s, 3H). ^13^C NMR (101 MHz, CDCl_3_) δ 169.75, 138.23, 138.16, 137.96, 128.39, 128.38, 128.33, 127.99, 127.83, 127.79, 127.76, 127.67, 127.55, 109.29, 108.65, 101.83, 96.20, 82.79, 77.94, 75.17, 75.03, 74.96, 73.51, 73.05, 71.30, 70.62, 70.53, 69.48, 68.60, 67.84, 26.06, 25.94, 25.10, 24.30, 20.95. The data are identical to the literature report [35].
*l*-Menthyl 2-*O*-acetyl-3,4,6-tri-*O*-benzyl-β-d-glucopyranoside (**17**): Following general procedure B, glycosyl acceptor **3g** (8.0 mg, 50.0 μmol) was coupled with EDPAT donor **2b** (54.0 mg, 75.0 μmol) to afford **17** (30.0 mg, 47.6 μmol, 94% yield) as a colorless viscous oil. R*_f_* = 0.6 (hexane:EtOAc = 70:30). ^1^H NMR (400 MHz, CDCl_3_) δ 7.34–7.30 (m, 7H), 7.30–7.26 (m, 6H), 7.23 (dd, *J* = 7.8, 1.8 Hz, 2H), 4.94 (dd, *J* = 9.0, 8.0 Hz, 1H), 4.80 (dd, *J* = 11.2, 4.3 Hz, 2H), 4.67 (d, *J* = 11.5 Hz, 1H), 4.64–4.60 (m, 2H), 4.55 (d, *J* = 12.1 Hz, 1H), 4.40 (d, *J* = 8.0 Hz, 1H), 3.73–3.64 (m, 4H), 3.44 (ddd, *J* = 9.3, 4.5, 2.1 Hz, 1H), 3.39 (td, *J* = 10.7, 4.3 Hz, 1H), 2.31 (td, *J* = 7.0, 2.7 Hz, 1H), 1.96 (s, 3H), 1.93 (d, *J* = 3.2 Hz, 1H), 1.63 (ddt, *J* = 13.0, 6.3, 3.3 Hz, 2H), 1.33 (dtd, *J* = 12.0, 6.0, 3.2 Hz, 1H), 1.23–1.18 (m, 1H), 0.95–0.93 (m, 1H), 0.90 (d, *J* = 6.5 Hz, 3H), 0.88 (d, *J* = 7.1 Hz, 3H), 0.83–0.80 (m, 1H), 0.78 (d, *J* = 6.9 Hz, 3H). ^13^C NMR (101 MHz, CDCl_3_) δ 169.41, 138.33, 138.30, 137.98, 128.42, 128.37, 128.33, 128.11, 127.83, 127.81, 127.63, 127.60, 127.51, 98.96, 83.14, 78.33, 78.10, 75.10, 75.02, 74.83, 73.71, 73.42, 69.14, 47.50, 40.89, 34.30, 31.38, 24.99, 23.00, 22.29, 21.00, 20.97, 15.76. The data are identical to the literature report [33].
Adamantanyl 2-*O*-acetyl-3,4,6-tri-*O*-benzyl-β-d-glucopyranoside (**18**): Following general procedure B, glycosyl acceptor **3h** (8.0 mg, 50.0 μmol) was coupled with EDPAT donor **2b** (54.0 mg, 75.0 μmol) to afford **18** (29.0 mg, 46.3 μmol, 87% yield) as a colorless viscous oil. R*_f_* = 0.7 (hexane:EtOAc = 70:30). ^1^H NMR (400 MHz, CDCl_3_) δ 7.34 (d, *J* = 7.3 Hz, 2H), 7.32 (d, *J* = 6.0 Hz, 6H), 7.28 (d, *J* = 8.2 Hz, 5H), 7.16 (dd, *J* = 7.5, 1.9 Hz, 2H), 5.46 (d, *J* = 3.8 Hz, 1H), 4.81 (d, *J* = 2.5 Hz, 1H), 4.80 (s, 1H), 4.78–4.74 (m, 1H), 4.66 (d, *J* = 12.1 Hz, 1H), 4.52–4.48 (m, 2H), 4.04 (t, *J* = 9.5 Hz, 2H), 3.78 (dd, *J* = 10.8, 3.9 Hz, 1H), 3.70 (t, *J* = 9.4 Hz, 1H), 3.65 (dd, *J* = 10.7, 2.2 Hz, 1H), 2.13–2.09 (m, 3H), 2.02 (s, 3H), 1.78 (dd, *J* = 10.8, 1.8 Hz, 3H), 1.73–1.69 (m, 3H), 1.62 (d, *J* = 11.9 Hz, 3H), 1.59–1.55 (m, 3H). ^13^C NMR (101 MHz, CDCl_3_) δ 170.42, 138.86, 138.19, 138.14, 128.37, 128.34, 128.30, 127.99, 127.78, 127.72, 127.56, 127.49, 127.44, 88.80, 80.41, 78.04, 75.28, 75.14, 74.43, 74.17, 73.43, 69.90, 68.68, 42.30, 36.20, 30.54, 21.02. The data are identical to the literature report [33].
Cholesteryl 2-*O*-acetyl-3,4,6-tri-*O*-benzyl-β-d-glucopyranoside (**19**): Following general procedure B, glycosyl acceptor **3i** (19.0 mg, 50.0 μmol) was coupled with EDPAT donor **2b** (54.0 mg, 75.0 μmol) to afford **19** (32.0 mg, 38.4 μmol, 78% yield) as a colorless viscous oil. R*_f_* = 0.7 (hexane:EtOAc = 70:30). ^1^H NMR (400 MHz, CDCl_3_) δ 7.34–7.29 (m, 7H), 7.28 (ddd, *J* = 8.8, 3.6, 1.4 Hz, 6H), 7.21–7.19 (m, 2H), 5.34–5.32 (m, 1H), 4.96 (dd, *J* = 9.3, 8.0 Hz, 1H), 4.79 (dd, *J* = 11.1, 4.3 Hz, 2H), 4.67 (d, *J* = 11.4 Hz, 1H), 4.62 (d, *J* = 12.2 Hz, 1H), 4.56 (dd, *J* = 11.5, 3.9 Hz, 2H), 4.43 (d, *J* = 8.0 Hz, 1H), 3.76–3.73 (m, 1H), 3.70–3.64 (m, 3H), 3.50–3.45 (m, 2H), 2.24–2.18 (m, 1H), 1.97 (s, 6H), 1.83 (dt, *J* = 13.4, 3.0 Hz, 2H), 1.62–1.44 (m, 8H), 1.36–1.33 (m, 2H), 1.26 (td, *J* = 6.3, 3.3 Hz, 2H), 1.17–1.02 (m, 7H), 0.99 (s, 5H), 0.92 (d, *J* = 6.5 Hz, 4H), 0.87 (dd, *J* = 6.6, 2.6 Hz, 6H), 0.68 (s, 3H). ^13^C NMR (101 MHz, CDCl_3_) δ 169.42, 140.63, 138.21, 137.89, 128.41, 128.39, 128.31, 128.03, 127.86, 127.83, 127.68, 127.67, 127.53, 121.83, 99.95, 83.02, 79.54, 78.13, 77.20, 75.11, 75.02, 74.94, 73.42, 73.36, 68.90, 56.75, 56.14, 50.18, 42.31, 39.76, 39.51, 39.05, 37.26, 36.71, 36.18, 35.77, 31.94, 31.86, 29.61, 28.22, 28.01, 24.28, 23.80, 22.81, 22.55, 21.04, 20.96, 19.37, 18.71, 11.84. The data are identical to the literature report [33].
Methyl (2,3,4,6-tetra-*O*-benzoyl-β-d-glucopyranosyl)-(1→6)-2,3,4-tri-*O*-benzyl-α-d-glucopyranoside (**20**): Following general procedure B, glycosyl acceptor **3a** (24.0 mg, 50.0 μmol) was coupled with EDPAT donor **2c** (62.0 mg, 75.0 μmol) to afford **20** (47.0 mg, 45.2 μmol, 88% yield) as a colorless viscous oil. R*_f_* = 0.3 (hexane:EtOAc = 70:30). ^1^H NMR (400 MHz, CDCl_3_) δ 7.98–7.96 (m, 2H), 7.87 (ddd, *J* = 8.3, 3.2, 1.3 Hz, 5H), 7.80 (dd, *J* = 8.4, 1.3 Hz, 2H), 7.53–7.45 (m, 3H), 7.38 (dd, *J* = 15.1, 7.8 Hz, 6H), 7.33–7.29 (m, 6H), 7.29–7.25 (m, 7H), 7.24–7.17 (m, 6H), 7.04–7.01 (m, 2H), 5.87 (t, *J* = 9.7 Hz, 1H), 5.65 (t, *J* = 9.7 Hz, 1H), 5.58 (dd, *J* = 9.8, 7.8 Hz, 1H), 4.87 (d, *J* = 10.9 Hz, 1H), 4.80 (d, *J* = 7.8 Hz, 1H), 4.72 (d, *J* = 12.1 Hz, 1H), 4.66 (d, *J* = 11.0 Hz, 1H), 4.61–4.56 (m, 2H), 4.52–4.45 (m, 3H), 4.26 (d, *J* = 11.0 Hz, 1H), 4.13 (d, *J* = 8.7 Hz, 1H), 4.11–4.05 (m, 1H), 3.86 (t, *J* = 9.3 Hz, 1H), 3.75–3.68 (m, 2H), 3.41 (dd, *J* = 9.7, 3.6 Hz, 1H), 3.36 (t, *J* = 9.3 Hz, 1H), 3.19 (s, 3H). ^13^C NMR (101 MHz, CDCl_3_) δ 166.23, 165.95, 165.27, 165.03, 138.87, 138.26, 138.22, 133.55, 133.37, 133.25, 133.23, 129.92, 129.86, 129.85, 129.82, 129.80, 129.62, 129.23, 128.85, 128.80, 128.54, 128.52, 128.46, 128.44, 128.41, 128.38, 128.24, 128.01, 127.99, 127.71, 127.59, 127.57, 101.41, 98.04, 81.97, 79.79, 75.65, 74.81, 73.49, 72.91, 72.27, 71.86, 69.84, 69.52, 68.38, 63.32, 55.11. The data are identical to the literature report [36].
Methyl (2,3,4,6-tetra-*O*-benzoyl-β-d-glucopyranosyl)-(1→6)-2,3,4-tri-*O*-benzyl-α-d-mannopyranoside (**21**): Following general procedure B, glycosyl acceptor **3d** (25.0 mg, 50.0 μmol) was coupled with EDPAT donor **2c** (59.0 mg, 75.0 μmol) to afford **21** (49.0 mg, 45.2 μmol, 91% yield) as a colorless viscous oil. R*_f_* = 0.2 (hexane:EtOAc = 70:30). ^1^H NMR (400 MHz, CDCl_3_) δ 8.08–8.04 (m, 2H), 8.00–7.97 (m, 2H), 7.93 (dd, *J* = 8.3, 1.4 Hz, 2H), 7.89–7.85 (m, 4H), 7.82 (dd, *J* = 8.5, 1.4 Hz, 2H), 7.76 (dd, *J* = 8.3, 1.3 Hz, 2H), 7.64–7.59 (m, 1H), 7.56–7.45 (m, 6H), 7.44–7.37 (m, 5H), 7.33 (td, *J* = 8.1, 3.9 Hz, 6H), 7.30–7.27 (m, 2H), 7.22 (d, *J* = 7.5 Hz, 2H), 5.90 (t, *J* = 9.7 Hz, 1H), 5.78 (dd, *J* = 9.9, 3.4 Hz, 1H), 5.69–5.62 (m, 2H), 5.60–5.56 (m, 1H), 5.54 (dd, *J* = 3.5, 1.8 Hz, 1H), 4.97 (d, *J* = 7.9 Hz, 1H), 4.64–4.59 (m, 2H), 4.43 (dd, *J* = 12.1, 5.1 Hz, 1H), 4.25 (t, *J* = 8.5 Hz, 1H), 4.15 (d, *J* = 14.9 Hz, 2H), 3.85 (dd, *J* = 11.1, 8.4 Hz, 1H), 3.11 (s, 3H). ^13^C NMR (101 MHz, CDCl_3_) δ 166.21, 165.90, 165.77, 165.58, 165.39, 165.26, 165.24, 133.57, 133.54, 133.35, 133.29, 133.24, 130.02, 129.92, 129.90, 129.86, 129.83, 129.77, 129.42, 128.90, 128.85, 128.83, 128.70, 128.54, 128.49, 128.40, 128.33, 102.02, 98.05, 72.90, 72.29, 71.91, 70.51, 69.91, 69.80, 69.71, 67.41, 63.08, 54.96, 29.81. The data are identical to the literature report [37].
Methyl (2,3,4,6-tetra-*O*-benzyl-α/β-d-mannopyranosyl)-(1→6)-2,3,4-tri-*O*-benzyl-α-d-glucopyranoside (**22**): Following general procedure B, glycosyl acceptor **3a** (24.0 mg, 50.0 μmol) was coupled with EDPAT donor **2d** (58.0 mg, 77.5 μmol) to afford **22** (27.0 mg, 27.4 μmol, 53% yield) as a colorless viscous oil. R*_f_* = 0.3 (hexane:EtOAc = 70:30). ^1^H NMR (400 MHz, CDCl_3_) δ 7.39–7.20 (m, 37H), 7.17–7.13 (m, 2H), 4.99 (d, *J* = 10.9 Hz, 2H), 4.87 (dd, *J* = 10.9, 7.8 Hz, 2H), 4.82–4.75 (m, 3H), 4.73–4.65 (m, 4H), 4.64–4.59 (m, 3H), 4.57 (d, *J* = 3.5 Hz, 1H), 4.52–4.42 (m, 4H), 3.99 (q, *J* = 9.4 Hz, 3H), 3.88–3.81 (m, 2H), 3.79 (s, 1H), 3.73–3.57 (m, 6H), 3.46 (dd, *J* = 9.7, 3.4 Hz, 1H), 3.43–3.37 (m, 1H), 3.31 (s, 3H). ^13^C NMR (101 MHz, CDCl_3_) δ 138.66, 138.63, 138.46, 138.42, 138.36, 138.18, 138.13, 128.48, 128.40, 128.35, 128.26, 128.21, 128.01, 127.99, 127.93, 127.84, 127.75, 127.70, 127.64, 127.60, 127.59, 127.55, 127.48, 127.44, 127.37, 98.24, 97.81, 82.13, 79.98, 79.56, 77.62, 75.80, 75.01, 74.92, 74.86, 73.25, 72.42, 71.98, 71.93, 69.80, 69.11, 65.79, 55.07. The data are identical to the literature report [38].
Methyl (2,3,4,6-tetra-*O*-benzyl-d-mannopyranosyl)-(1→6) 2,3,4-tri-*O*-benzoyl-α-d-glucopyranoside (**23**): Following general procedure B, glycosyl acceptor **3b** (25.0 mg, 50.0 μmol) was coupled with EDPAT donor **2d** (55.0 mg, 77.5 μmol) to afford **23** (36.0 mg, 35.0 μmol, 71% yield) as a colorless viscous oil. R*_f_* = 0.2 (hexane:EtOAc = 70:30). ^1^H NMR (400 MHz, CDCl_3_) δ 8.01–7.97 (m, 2H), 7.93–7.86 (m, 4H), 7.52 (t, *J* = 7.4 Hz, 1H), 7.47–7.33 (m, 10H), 7.29 (dt, *J* = 9.8, 5.1 Hz, 16H), 7.20–7.16 (m, 2H), 6.13 (t, *J* = 9.9 Hz, 1H), 5.57 (t, *J* = 9.9 Hz, 1H), 5.24 (dd, *J* = 10.1, 3.6 Hz, 1H), 5.18 (d, *J* = 3.6 Hz, 1H), 4.93–4.91 (m, 1H), 4.88 (d, *J* = 10.9 Hz, 1H), 4.75–4.66 (m, 2H), 4.59–4.52 (m, 1H), 4.47 (dd, *J* = 11.2, 7.8 Hz, 3H), 4.40 (d, *J* = 12.1 Hz, 1H), 3.99–3.85 (m, 3H), 3.70 (s, 1H), 3.68–3.60 (m, 3H), 3.55 (d, *J* = 9.0 Hz, 1H), 3.39 (s, 3H). ^13^C NMR (101 MHz, CDCl_3_) δ 165.83, 165.80, 165.06, 138.69, 138.59, 138.47, 138.40, 133.34, 133.23, 133.04, 129.92, 129.89, 129.65, 129.10, 129.08, 128.40, 128.37, 128.29, 128.26, 128.21, 127.91, 127.75, 127.66, 127.65, 127.49, 127.43, 127.35, 98.19, 96.91, 79.87, 74.99, 74.75, 73.20, 72.55, 72.10, 72.05, 71.92, 70.51, 69.83, 69.03, 68.07, 66.16, 55.53. The data are identical to the literature report [30].
*l*-Menthyl 2,3,4,6-tetra-*O*-benzyl-d-mannopyranoside (**24**): Following general procedure B, glycosyl acceptor **3g** (8.0 mg, 50.0 μmol) was coupled with EDPAT donor **2d** (57.0 mg, 75.0 μmol) to afford **24** (22.0 mg, 32.4 μmol, 64% yield) as a colorless viscous oil. R*_f_* = 0.6 (hexane:EtOAc = 70:30). α-anomer: ^1^H NMR (400 MHz, CDCl_3_) δ 7.37–7.27 (m, 18H), 7.15 (dd, *J* = 7.5, 2.0 Hz, 2H), 5.04 (d, *J* = 3.7 Hz, 1H), 4.99 (d, *J* = 11.0 Hz, 1H), 4.87–4.82 (m, 2H), 4.74–4.68 (m, 2H), 4.65 (d, *J* = 12.1 Hz, 1H), 4.48 (dd, *J* = 11.4, 5.1 Hz, 2H), 4.03 (t, *J* = 9.3 Hz, 1H), 3.99 (ddd, *J* = 10.1, 4.0, 2.2 Hz, 1H), 3.77 (dd, *J* = 10.5, 4.0 Hz, 1H), 3.67–3.63 (m, 2H), 3.56 (dd, *J* = 9.8, 3.7 Hz, 1H), 3.37 (dd, *J* = 10.6, 4.5 Hz, 1H), 2.43 (pd, *J* = 7.1, 2.6 Hz, 1H), 2.17–2.13 (m, 1H), 1.62 (dp, *J* = 13.2, 3.3 Hz, 2H), 1.41–1.30 (m, 2H), 1.08–1.02 (m, 1H), 0.96–0.92 (m, 1H), 0.86 (dd, *J* = 6.7, 3.2 Hz, 7H), 0.72 (d, *J* = 6.9 Hz, 3H). ^13^C NMR (101 MHz, CDCl_3_) δ 138.91, 138.38, 138.30, 138.05, 128.34, 128.32, 128.30, 128.23, 127.89, 127.87, 127.63, 127.62, 127.61, 127.48, 127.46, 98.62, 81.97, 80.99, 80.54, 75.47, 75.03, 73.44, 73.18, 70.29, 68.66, 48.75, 43.04, 34.26, 31.73, 24.57, 22.95, 22.28, 21.10, 16.05. β-anomer: ^1^H NMR (400 MHz, CDCl_3_) δ 7.36–7.32 (m, 5H), 7.31–7.26 (m, 9H), 7.20 (dd, *J* = 7.8, 1.8 Hz, 1H), 4.94 (dd, *J* = 15.9, 11.1 Hz, 2H), 4.80 (dd, *J* = 16.7, 11.0 Hz, 2H), 4.69 (d, *J* = 10.8 Hz, 1H), 4.63–4.53 (m, 3H), 4.48 (d, *J* = 7.9 Hz, 1H), 3.70 (d, *J* = 3.4 Hz, 2H), 3.65–3.58 (m, 2H), 3.51 (td, *J* = 10.8, 4.3 Hz, 1H), 3.43–3.39 (m, 2H), 2.35 (pd, *J* = 6.8, 2.5 Hz, 1H), 2.16–2.12 (m, 1H), 1.69–1.64 (m, 2H), 1.39–1.32 (m, 1H), 1.30–1.27 (m, 1H), 1.04–0.94 (m, 2H), 0.92 (dd, *J* = 12.2, 6.8 Hz, 6H), 0.89–0.84 (m, 1H), 0.83 (d, *J* = 6.9 Hz, 3H). ^13^C NMR (101 MHz, CDCl_3_) δ 138.82, 138.56, 138.38, 138.22, 128.36, 128.30, 128.27, 128.06, 127.76, 127.70, 127.61, 127.57, 127.48, 127.46, 100.75, 84.96, 82.21, 77.96, 77.74, 75.57, 74.97, 74.80, 73.66, 69.33, 48.12, 40.96, 34.45, 31.46, 25.27, 23.21, 22.23, 21.07, 15.95. The data are identical to the literature report [39].
Propargyl 2-*O*-acetyl-3,4,6-tri-*O*-benzyl-α-d-mannopyranosyl-(1→6) 2,3,4-tri-*O*-benzyl-α-d-mannopyranoside (**25**): Following general procedure B, glycosyl acceptor **3k** (25.0 mg, 51.0 μmol) was coupled with EDPAT donor **2a** (54.0 mg, 77.5 μmol) to afford **25** (38.0 mg, 39.5 μmol, 77% yield) as a colorless viscous oil. R*_f_* = 0.3 (hexane:EtOAc = 70:30). ^1^H NMR (400 MHz, CDCl_3_) δ 7.42–7.18 (m, 28H), 7.17–7.09 (m, 2H), 5.46 (dd, *J* = 3.2, 1.9 Hz, 1H), 4.99 (dd, *J* = 27.0, 1.8 Hz, 2H), 4.88 (dd, *J* = 21.7, 11.0 Hz, 2H), 4.73 (s, 2H), 4.69–4.62 (m, 2H), 4.59 (d, *J* = 1.5 Hz, 2H), 4.45 (ddd, *J* = 13.8, 11.6, 4.9 Hz, 4H), 4.13 (dd, *J* = 4.0, 2.4 Hz, 2H), 3.98–3.81 (m, 6H), 3.80–3.76 (m, 1H), 3.69 (td, *J* = 10.9, 10.1, 2.6 Hz, 3H), 3.59 (dd, *J* = 10.9, 1.9 Hz, 1H), 2.38 (t, *J* = 2.4 Hz, 1H), 2.15 (s, 3H). ^13^C NMR (101 MHz, CDCl_3_) δ 170.49, 138.69, 138.47, 138.35, 138.25, 137.91, 128.56, 128.52, 128.50, 128.46, 128.40, 128.34, 128.05, 127.95, 127.89, 127.85, 127.80, 127.77, 127.73, 127.67, 127.59, 98.10, 96.38, 80.14, 78.93, 75.16, 74.94, 74.56, 74.43, 74.24, 73.46, 72.80, 72.11, 71.64, 71.53, 71.47, 68.78, 68.61, 66.66, 54.30, 29.86, 21.34. The data are identical to the literature report [31].
Methyl 6-*O*-(6-*O*-(2-*O*-Acetyl-3,4,6-tri-*O*-benzyl-α-d-mannopyranosyl)-2,3,4-tri-*O*-benzyl-α-d-mannopyranosyl)-2,3,4-tri-*O*-benzyl-α-d-mannopyranoside (**26**): Procedure 1: This was prepared according to the literature procedure [40] using donor **25** (7 μmol), acceptor **3b** (1.2 equiv.), and AuCl_3_ (1 mol%) in acetonitrile at 60 °C in 3 h (76%, α:β = 2:1). Procedure 2: This was prepared according to the literature procedure [41] using donor **27** (20 μmol), acceptor **3b** (1.1 equiv.), and NIS (1.5 equiv.)/TMSOTf (10 mol%) in CH_2_Cl_2_ at 0 °C in 3 h (52%, α:β = 2:1). ^1^H NMR (400 MHz, CDCl_3_) δ 7.47–7.44 (m, 1H), 7.40–7.37 (m, 2H), 7.34 (dt, *J* = 4.3, 2.4 Hz, 9H), 7.32–7.26 (m, 20H), 7.26–7.15 (m, 39H), 7.09 (dd, *J* = 6.6, 3.0 Hz, 4H), 5.49 (q, *J* = 3.2 Hz, 2H), 5.09 (d, *J* = 1.7 Hz, 1H), 5.00–4.87 (m, 7H), 4.85–4.80 (m, 3H), 4.73–4.52 (m, 19H), 4.50–4.35 (m, 12H), 4.20 (d, *J* = 16.7 Hz, 2H), 3.98–3.82 (m, 15H), 3.82–3.74 (m, 5H), 3.72–3.59 (m, 9H), 3.53 (ddd, *J* = 21.6, 10.8, 1.9 Hz, 4H), 3.40 (dd, *J* = 9.4, 3.0 Hz, 1H), 3.22 (s, 3H_α_), 3.18 (s, 1.5H_β_), 2.13 (s, 3H_α_), 2.11 (s, 1.5H_β_). ^13^C NMR (101 MHz, CDCl_3_) δ 170.22, 138.71, 138.69, 138.64, 138.62, 138.60, 138.46, 138.41, 138.24, 138.21, 138.17, 138.06, 137.84, 137.80, 128.61, 128.36, 128.33, 128.31, 128.26, 128.23, 128.17, 128.16, 128.14, 128.09, 127.92, 127.89, 127.86, 127.79, 127.78, 127.73, 127.71, 127.67, 127.64, 127.62, 127.60, 127.58, 127.53, 127.48, 127.44, 127.41, 127.39, 127.32, 127.27, 102.22, 98.85, 98.74, 98.16, 98.06, 97.81, 82.15, 80.27, 79.25, 77.80, 77.67, 77.22, 74.98, 74.85, 74.67, 74.56, 74.44, 74.27, 74.04, 73.52, 73.30, 72.88, 72.77, 72.62, 72.30, 72.05, 71.89, 71.41, 71.29, 71.23, 70.99, 68.55, 68.34, 66.40, 65.93, 54.67, 54.58, 21.18, 21.16. The data are identical to the literature report [31].
Phenyl 2-*O*-acetyl-3,4,6-tri-*O*-benzyl-α-d-mannopyranosyl-(1→6) 2,3,4-tri-*O*-benzyl-1-deoxy-1-thio-α-d-mannopyranoside (**27**): Following general procedure B, glycosyl acceptor **3m** (27.0 mg, 50.0 μmol) was coupled with EDPAT donor **2a** (54.0 mg, 77.5 μmol) to afford **27** (43.0 mg, 42.5 μmol, 84% yield) as a colorless viscous oil. R*_f_* = 0.3 (hexane:EtOAc = 70:30). ^1^H NMR (400 MHz, CDCl_3_) δ 7.32 (qd, *J* = 24.1, 4.6 Hz, 32H), 7.18–7.09 (m, 3H), 5.57 (s, 1H), 5.45 (s, 1H), 4.94 (d, *J* = 7.8 Hz, 2H), 4.86 (d, *J* = 10.8 Hz, 1H), 4.74 (d, *J* = 13.3 Hz, 1H), 4.71–4.57 (m, 6H), 4.50 (d, *J* = 10.9 Hz, 1H), 4.47–4.36 (m, 2H), 4.23 (dd, *J* = 10.7, 6.2 Hz, 1H), 4.01 (s, 1H), 3.97–3.89 (m, 3H), 3.89–3.76 (m, 3H), 3.70 (dd, *J* = 17.7, 12.6 Hz, 2H), 3.59 (d, *J* = 10.9 Hz, 1H), 2.14 (s, 3H). ^13^C NMR (101 MHz, CDCl_3_) δ 170.45, 138.70, 138.42, 138.32, 138.16, 137.97, 137.94, 134.79, 131.97, 131.01, 129.27, 128.60, 128.57, 128.54, 128.50, 128.41, 128.33, 128.09, 128.00, 127.91, 127.86, 127.82, 127.74, 127.68, 127.61, 127.34, 98.20, 86.11, 85.52, 80.38, 77.95, 76.19, 75.25, 74.71, 74.20, 73.44, 72.24, 72.08, 71.96, 71.62, 71.43, 68.71, 68.56, 66.87, 21.34. The data are identical to the literature report [31].

## 4. Conclusions

In conclusion, we have developed a sustainable and efficient glycosylation approach by utilizing copper, an earth-abundant catalyst, to activate ethyl 2-diazo-2-phenylacetate (EDPA)-based thioglycoside donors. This method provides an alternative to traditional glycosylation strategies that often require harsh conditions or precious metals. The versatility of this approach allows it to accommodate a broad range of nucleophiles, glycosyl donors, and protecting groups, demonstrating its wide applicability. Additionally, the orthogonality of this method to other thioglycoside and alkyne donors further enhances its utility. The successful application of this methodology to the iterative synthesis of trisaccharides, along with the mechanistic insights gained from investigating the electronic effects of different donor groups, highlights the potential of this approach for advancing glycosylation chemistry.

## Figures and Tables

**Table 1:** Optimization of the reaction conditions for EDPAT donor **2a** ^a^.

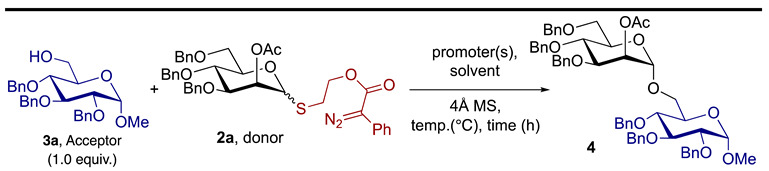						
entry	promoter(s)	donor (2a)	solvent(s)	time	temp.	4 (% yield) ^b^
1.	[Cu(OTf)]_2_•tol (30 mol%)	1.2 equiv.	CH_2_Cl_2_	5 h	rt	68% ^c^
2.	Fe(BF_4_)_2_•6H_2_O (30 mol%)	1.2 equiv.	CH_2_Cl_2_	20 h	rt	<10%
3.	ZnCl_2_ (30 mol%)	1.2 equiv.	CH_2_Cl_2_	6 h	rt	trace
4.	Zn(OTf)_2_ (30 mol%)	1.2 equiv.	CH_2_Cl_2_	72 h	rt	n.r.
5.	Rh_2_(OAc)_4_ (30 mol%)	1.2 equiv.	CH_2_Cl_2_	2 h	rt	<10%
6.	[Cu(CH_3_CN)_4_]PF_6_ (30 mol%)	1.2 equiv.	CH_2_Cl_2_	2 h	rt	40%
7.	[Cu(CH_3_CN)_4_]BF_4_ (30 mol%)	1.2 equiv.	CH_2_Cl_2_	72 h	rt	35%
8.	CuI (30 mol%)	1.2 equiv.	CH_2_Cl_2_	24 h	rt	n.r.
9.	Cu(OTf)_2_ (30 mol%)	1.2 equiv.	CH_2_Cl_2_	5 h	rt	61%
10.	TfOH (100 mol%)	1.2 equiv.	CH_2_Cl_2_	24 h	rt	n.r.
11.	[Cu(OTf)]_2_•tol (30 mol%)	1.2 equiv.	CH_2_Cl_2_	24 h	0 °C	70%
12.	[Cu(OTf)]_2_•tol (30 mol%)	1.5 equiv.	CH_2_Cl_2_	24 h	0 °C	74%
13.	[Cu(OTf)]_2_•tol (30 mol%)	1.5 equiv.	CH_2_Cl_2_	5 h	rt	75%
14.	[Cu(OTf)]_2_•tol (10 mol%)	1.5 equiv.	CH_2_Cl_2_	7 h	rt	75%
**15. ^d^ **	**[Cu(OTf)]_2_•tol (10 mol%)**	**1.5 equiv.**	**CH_2_Cl_2_**	**6 h**	**rt**	**88% ^c^**
16. ^d^	Cu(OTf)_2_ (10 mol%)	1.5 equiv.	CH_2_Cl_2_	5 h	rt	77% ^c^
17.	[Cu(OTf)]_2_•tol (10 mol%)	1.2 equiv.	CH_3_CN	6 h	rt	28% ^c^
18.	[Cu(OTf)]_2_•tol (10 mol%)	1.2 equiv.	THF	6 h	rt	<10%
19.	[Cu(OTf)]_2_•tol (10 mol%)	1.2 equiv.	Toluene	8 h	rt	56%
20	[Cu(OTf)]_2_•tol (10 mol%)	1.2 equiv.	EtOAc	8 h	rt	<10%
21.	-	1.2 equiv.	CH_2_Cl_2_	24 h	rt	n.r.

^a^ Reactions were conducted with an acceptor (0.025 mmol), donor (0.030–0.0375 mmol), promoters (0.0025–0.0075 mmol, 10–30 mol%), 4 Å molecular sieves (100 wt %), and CH_2_Cl_2_ (0.05 M). ^b^ % Yield was determined using ^1^H NMR, with 1,3,5-trimethoxybenzene as the internal standard. ^c^ Isolated yields. ^d^ The donor was added in portions to the reaction mixture. The blue color here highlights the optimized reaction conditions.

## Data Availability

Data are contained within the article and Supplementary Materials.

## Supplementary Materials

The following supporting information can be downloaded at: https://www.mdpi.com/article/10.3390/molecules29225367/s1, Figure S1: List of synthesized sugar anomeric thiols; Figure S2: List of synthesized diazo linkers; Figure S3: List of literature-known glycosyl acceptors; references for the synthesis of glycosyl thiols and diazo linkers; copy of the NMR spectra.
