# Supplementary material for: Catalytic Thioglycoside Activation with Diazo-Derived Copper Carbenes
Source: Molecules. 2024 Nov 14;29(22):5367. doi: 10.3390/molecules29225367 (PMC11597044; doi:10.3390/molecules29225367)
Supplement: Supplementary file 1 [file molecules-29-05367-s001.zip › molecules-3296605-supplementary.pdf]

Supporting Information
For

Catalytic Thioglycoside Activation with Diazo-derived Copper-Carbenes

Surya Pratap Singh[a], Umesh Chaudhary[a,] and Indrajeet Sharma[a]*

[a]Department of Chemistry and Biochemistry, University of Oklahoma, 101 Stephenson Parkway, Norman, OK 73019, USA,

E-mail: isharma@ou.edu;

Webpage: https://indrajeetsharma.com

Table of Contents

**Materials**	**S3**
**Figure S1. List of synthesized sugar anomeric thiols**	**S3**
**Figure S2. List of synthesized diazo linkers**	**S3**
**Figure S3. List of literature known glycosyl acceptors**	**S3**
**References**	**S4**
**Copy of NMR spectra for all new EDPA thioglycoside donors (2a-2f)**	**S5**
**Copy of NMR spectra for all literature known disaccharides and trisaccharide**	**S11**

MATERIALS:

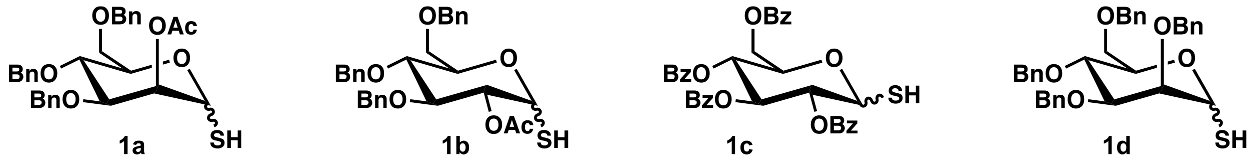

Figure S1. List of synthesized sugar anomeric thiols. The anomeric thiols were synthesized using literature-reported protocols {1a, 1b, 1d} [1] , and {1c} [2].

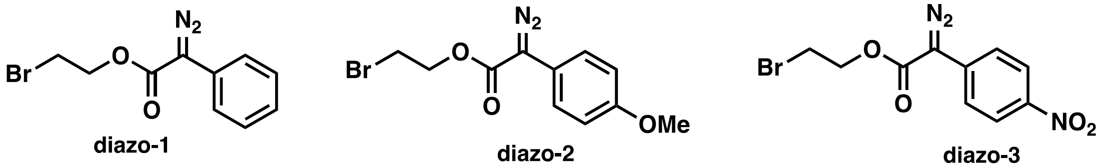

Figure S2. List of synthesized diazo linkers. The diazo linkers were synthesized using literature-reported protocols {diazo-1}[3], {diazo-2}[4].

Note: All the newly synthesized EDPA donors have been provided in Table 1 in the manuscript. The synthetic procedures and characterization data are also provided in the manuscript. In this file, we have attached copies of NMR spectra for all six EDPA thioglycoside donors.

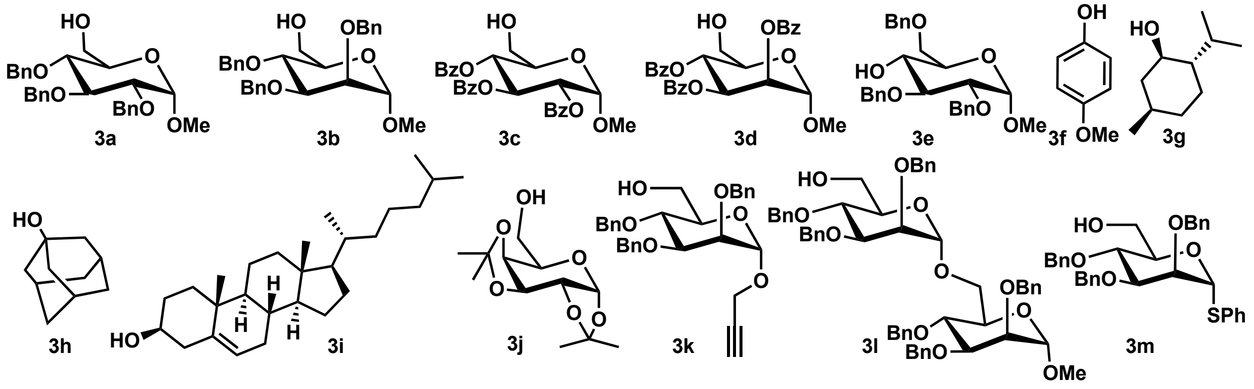

Figure S3. List of literature known glycosyl acceptors.

Note: All the compounds provided in Table 3 and Figure 2 are literature known. The respective literature has been cited in the manuscript. In this file, we have attached copies of NMR spectra for all literature-known compounds (disaccharides, and a trisaccharide)

Reference:

1. Johnston, B.D.; Pinto, B.M. Synthesis of Thio-Linked Disaccharides by 1→2 Intramolecular Thioglycosyl Migration: Oxacarbenium versus Episulfonium Ion Intermediates. *J. Org. Chem.* **2000**, *65*, 4607-4617.
2. Doyle, L.M.; O'Sullivan, S.; Di Salvo, C.; McKinney, M.; McArdle, P.; Murphy, P.V. Stereoselective Epimerizations of Glycosyl Thiols. *Org. Lett.* **2017**, *19*, 5802-5805.
3. Ba, D.; Wen, S.; Tian, Q.; Chen, Y.; Lv, W.; Cheng, G. Rhodium(II)-catalyzed multicomponent assembly of α,α,α-trisubstituted esters via formal insertion of O–C(sp3)–C(sp2) into C–C bonds. *Nat. Commun.* **2020**, *11*, 4219.
4. Liu, Z.; Xia, Y.; Feng, S.; Zhang, Y.; Wang, J. Rh(i)-Catalyzed coupling of 2-bromoethyl aryldiazoacetates with tertiary propargyl alcohols through carbene migratory insertion. *Org. Chem. Front.* **2016**, *3*, 1691-1698.

Copy of spectra for all new EDPA thioglycoside donors (2a-2f):

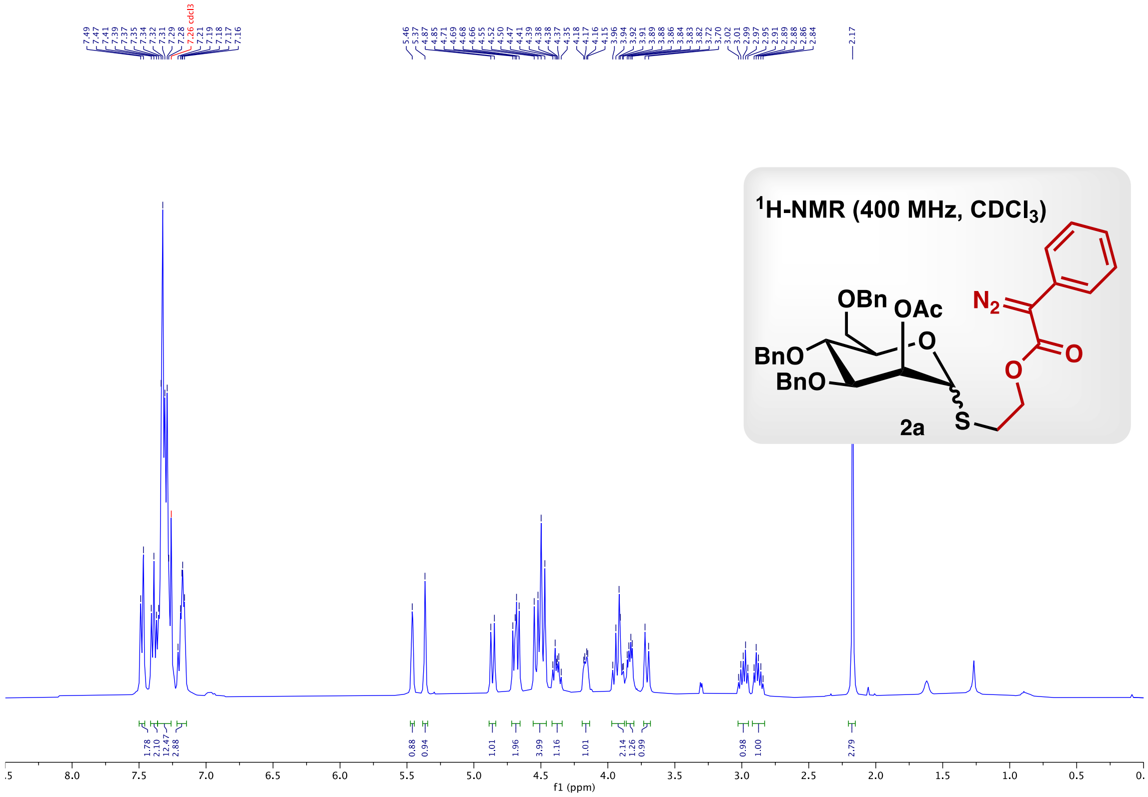

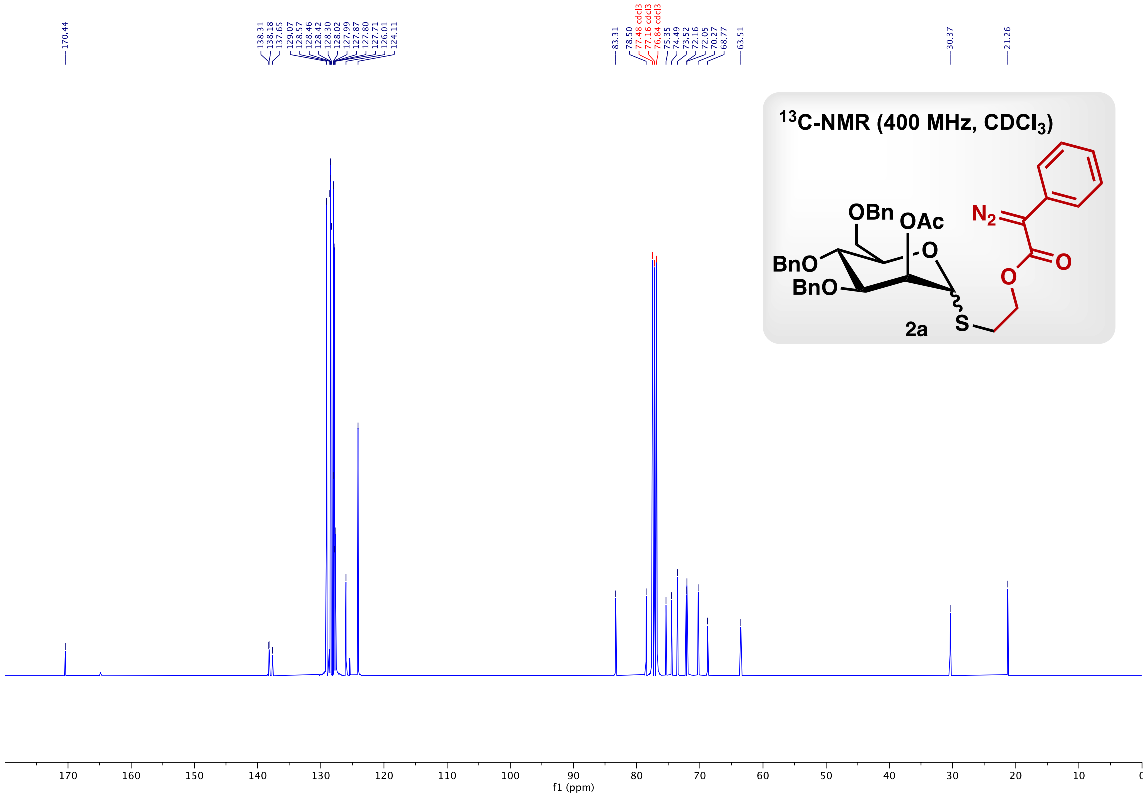

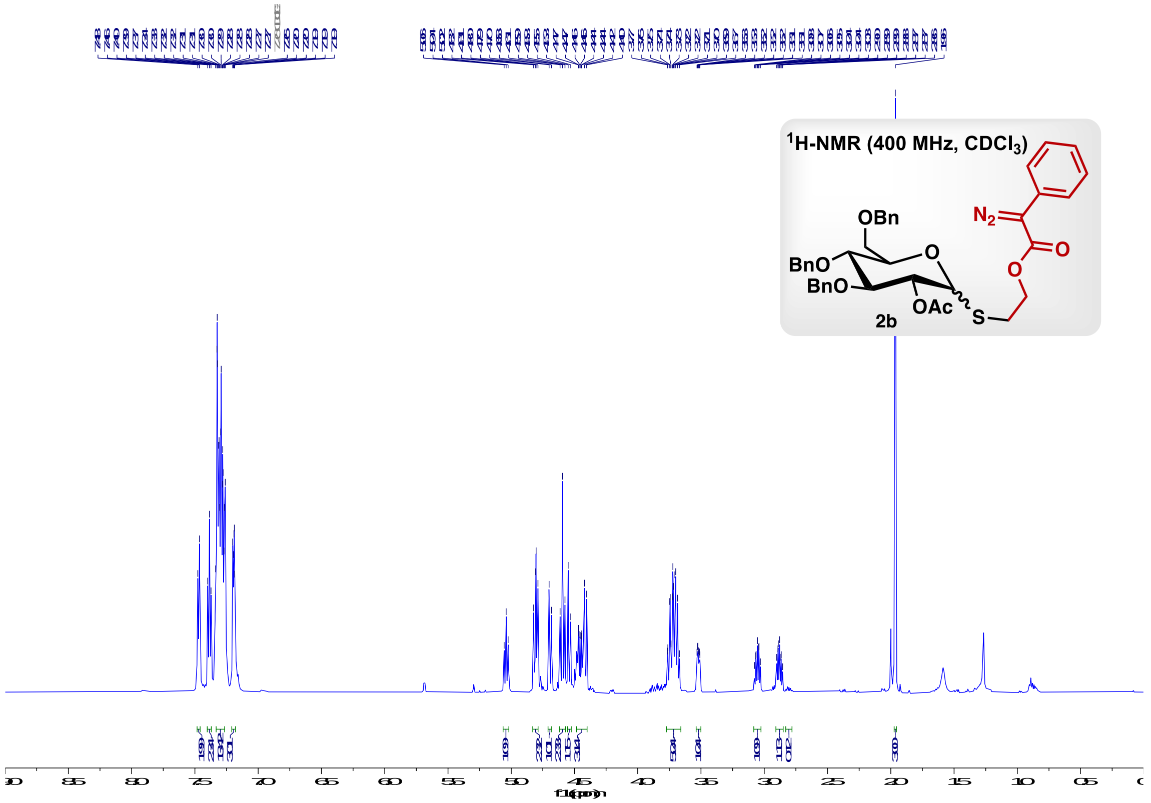

1H-NMR (400 MHz, CDCl3)
OBn
BnO
BnO
O
OAc
S
O
O
N2
2b
f1 (ppm)

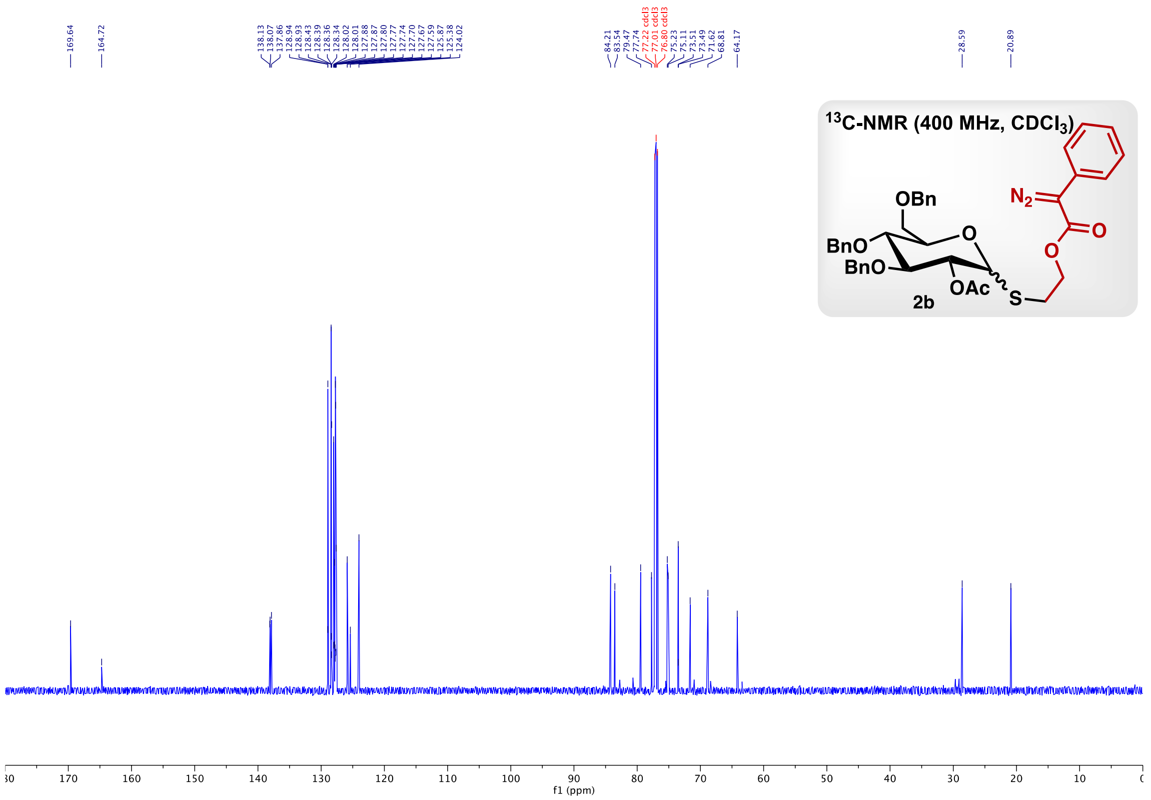

169.64
164.72
138.13
138.07
137.86
128.94
128.93
128.43
128.39
128.36
128.34
128.02
128.01
127.88
127.87
127.80
127.77
127.74
127.70
127.67
127.59
125.87
125.38
124.02
84.21
83.54
79.47
77.74
77.22 cdcl3
77.01 cdcl3
76.80 cdcl3
75.23
75.11
73.51
73.49
71.62
68.81
64.17
28.59
20.89
13C-NMR (400 MHz, CDCl3)
OBn
BnO
BnO
O
OAc
S
O
O
N2
2b
f1 (ppm)

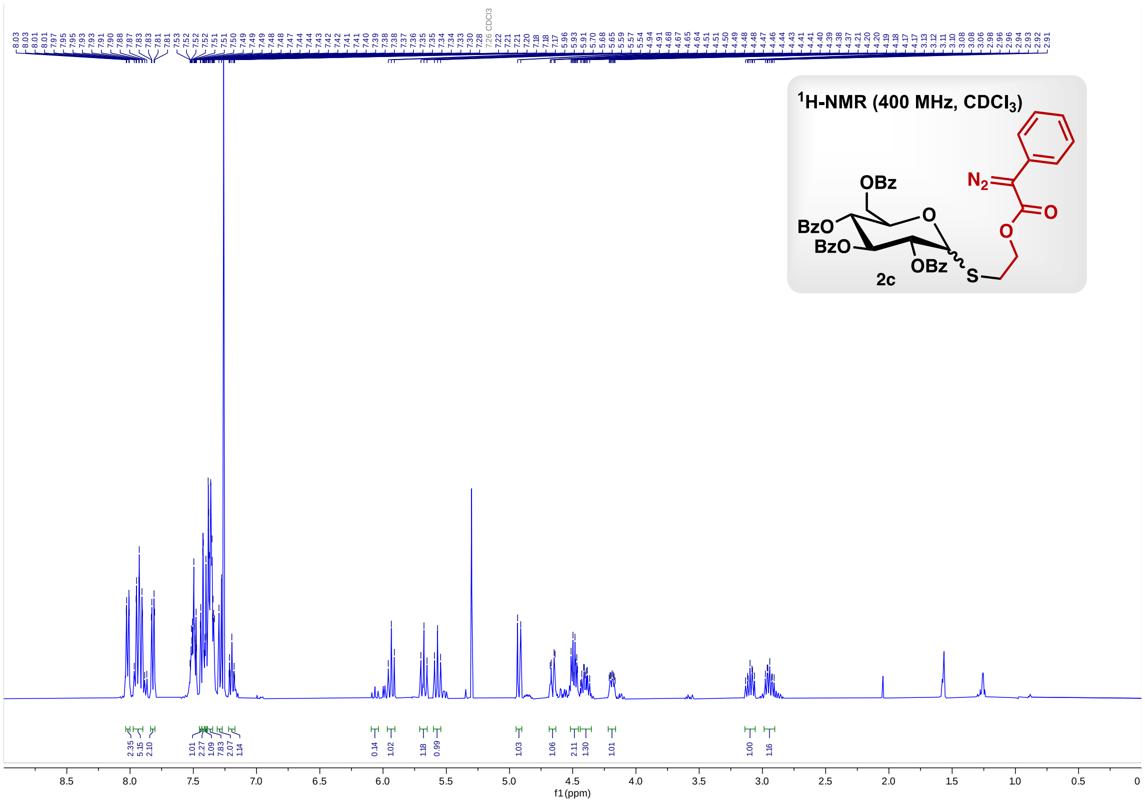
1H-NMR (400 MHz, CDCl3)
OBz
BzO
BzO
OBz
O
N2
O
O
S
2c
f1 (ppm)

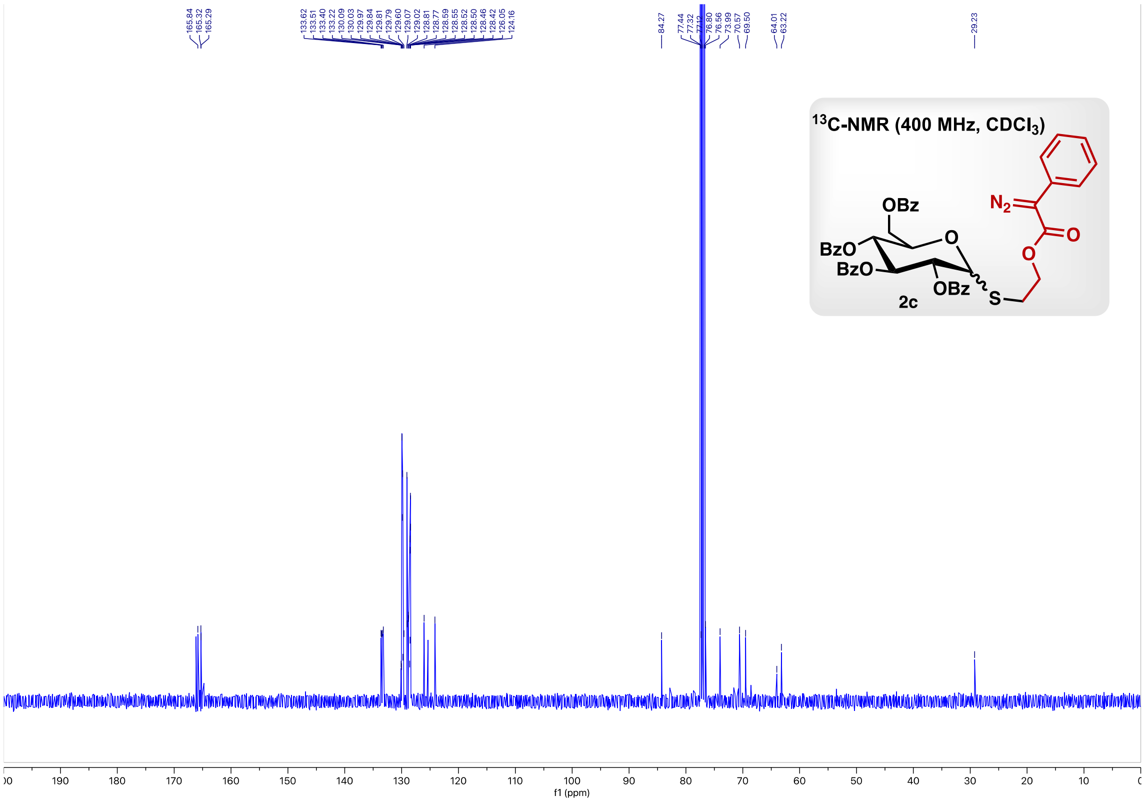
13C-NMR (400 MHz, CDCl3)
OBz
BzO
BzO
OBz
O
N2
O
O
S
2c
f1 (ppm)

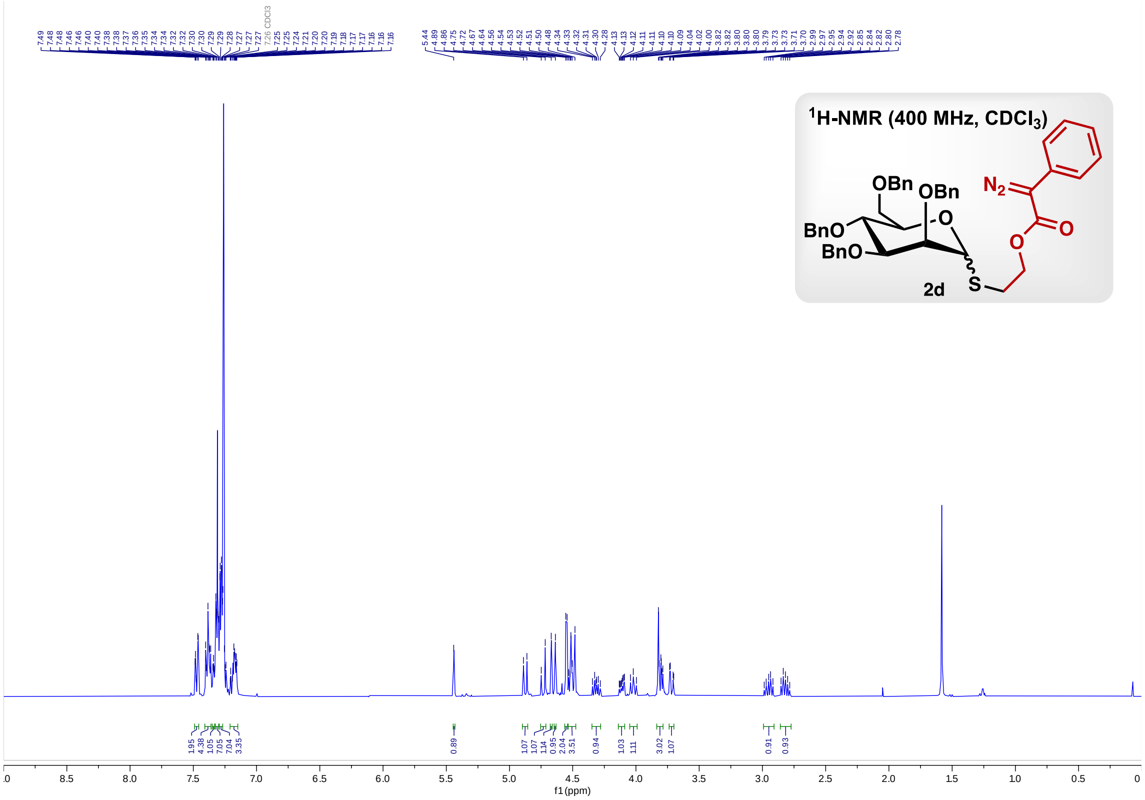

1H-NMR (400 MHz, CDCl3)
OBn
OBn
N2
BnO
O
O
O
BnO
2d
S
f1 (ppm)

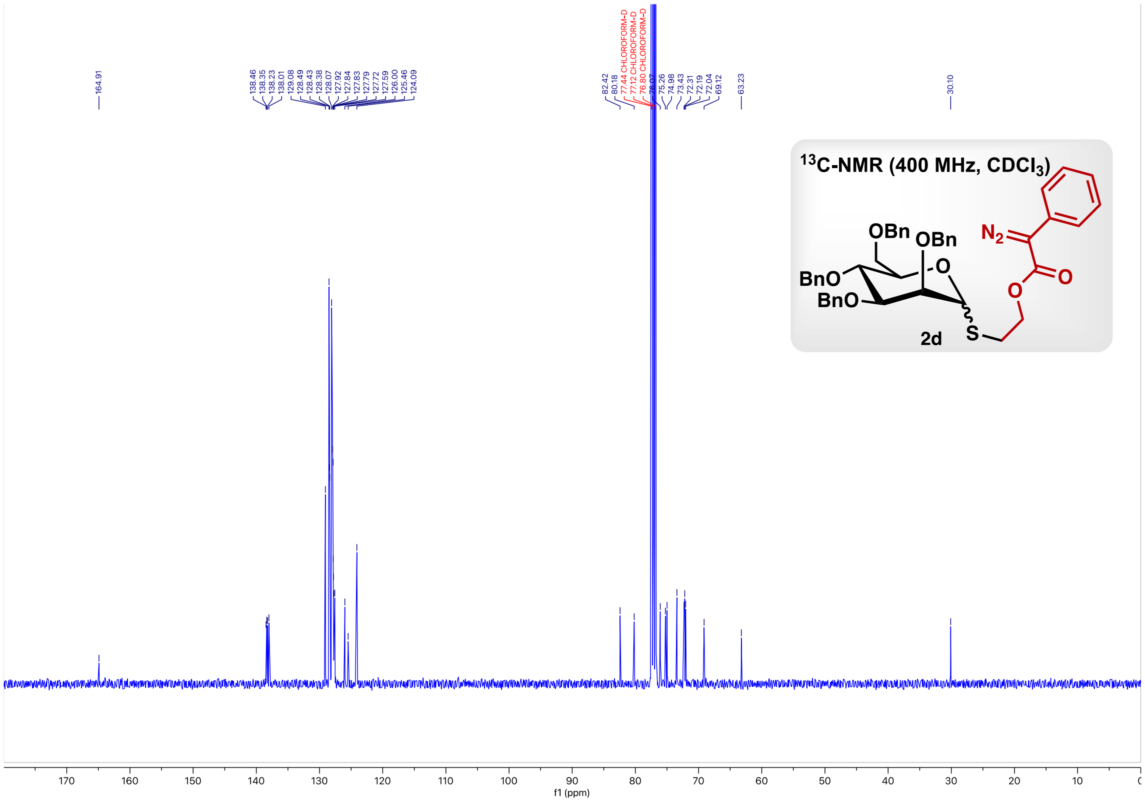

13C-NMR (400 MHz, CDCl3)
OBn
OBn
N2
BnO
O
O
O
BnO
2d
S
f1 (ppm)

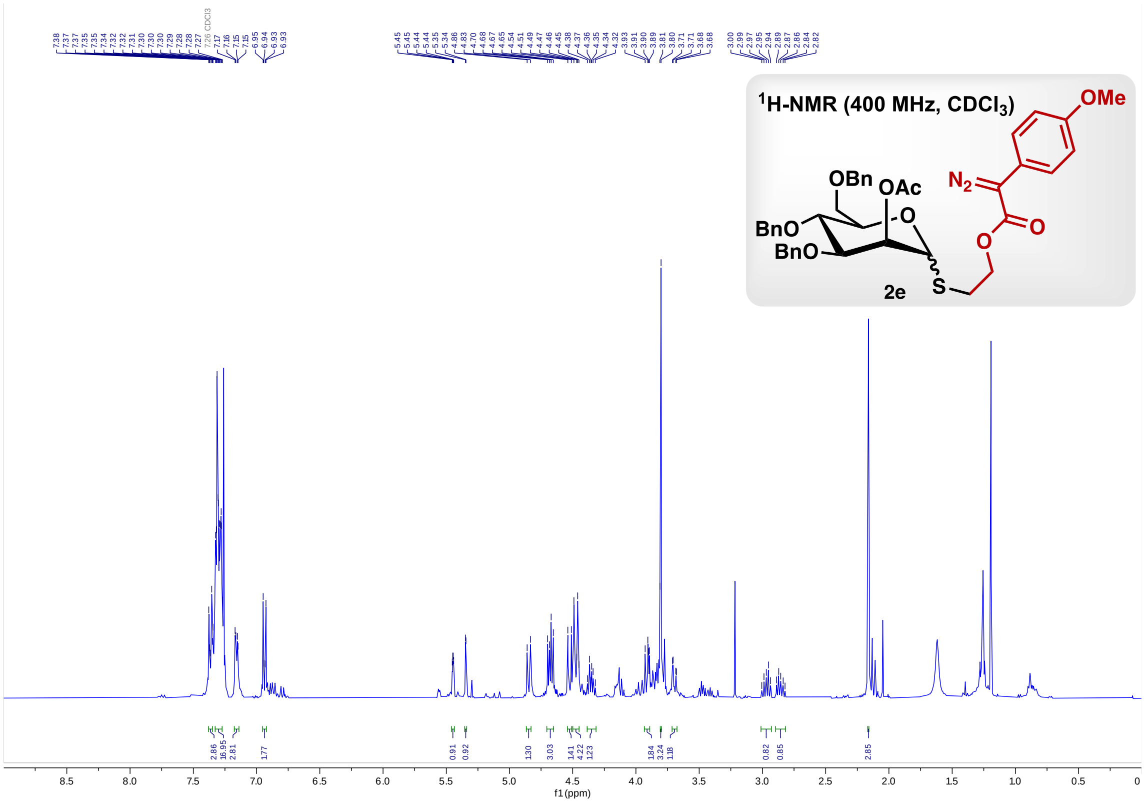

1H-NMR (400 MHz, CDCl3)
OMe
N2
OBn
OAc
O
BnO
BnO
S
2e
f1 (ppm)

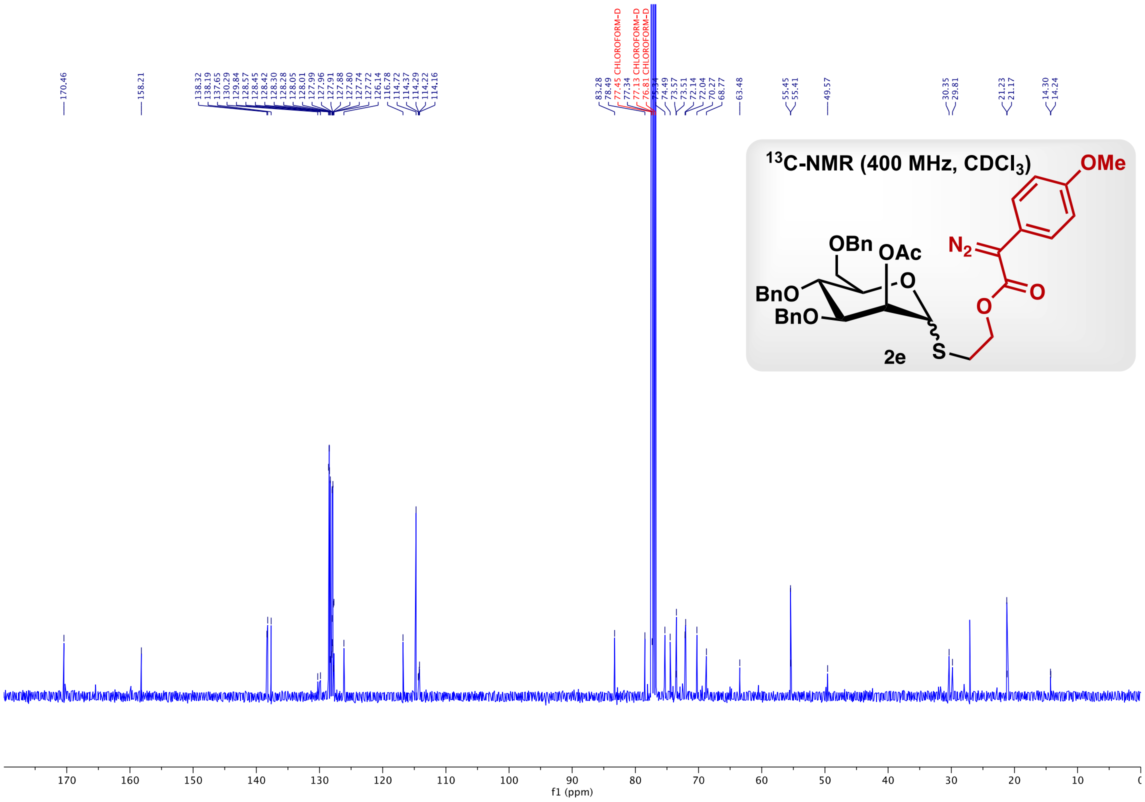

13C-NMR (400 MHz, CDCl3)
OMe
N2
OBn
OAc
O
BnO
BnO
S
2e
f1 (ppm)

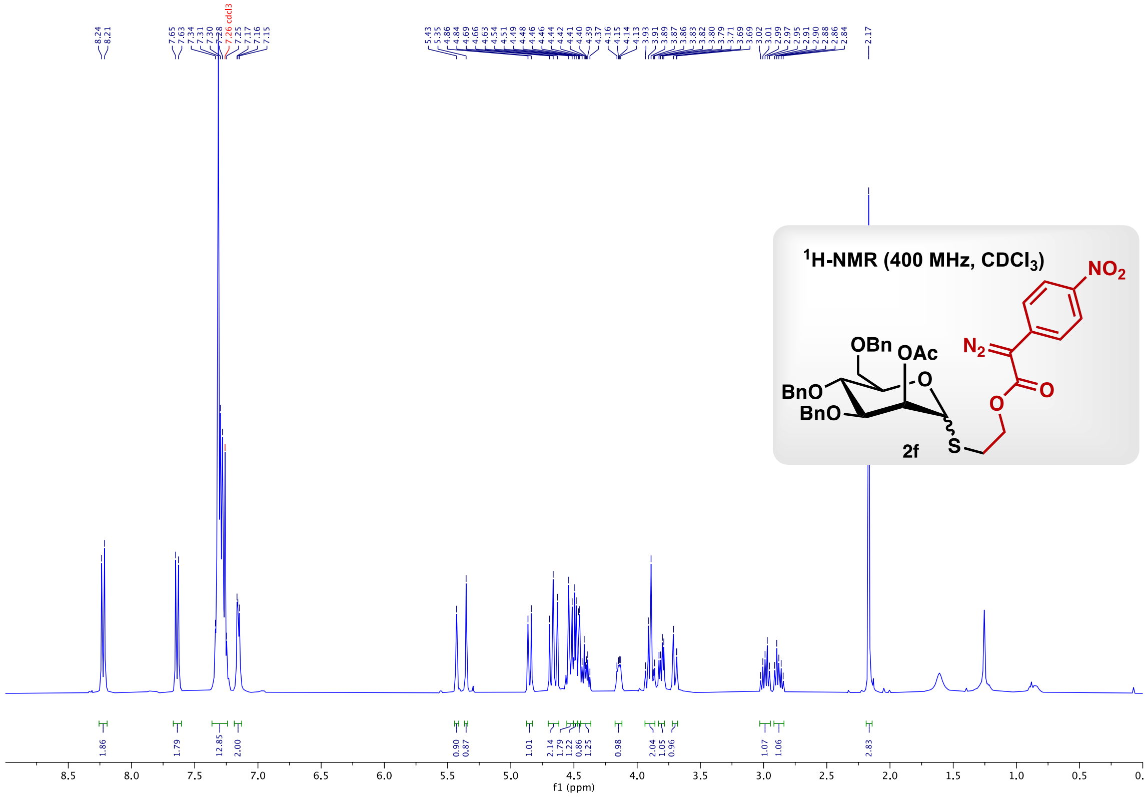

1H-NMR (400 MHz, CDCl3)
NO2
N2
OBn
OAc
BnO
BnO
O
O
O
S
2f
f1 (ppm)

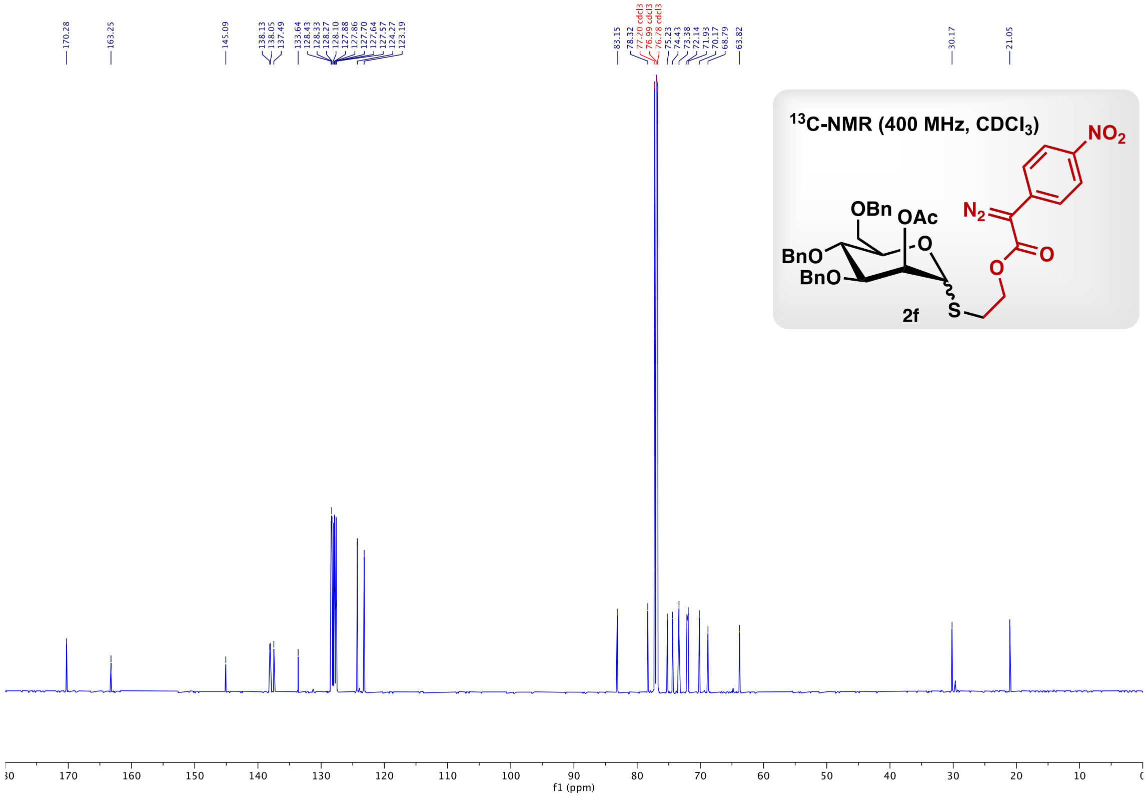

13C-NMR (400 MHz, CDCl3)
NO2
N2
OBn
OAc
BnO
BnO
O
O
O
S
2f
f1 (ppm)

Copy of NMR spectra for all literature known disaccharides and trisaccharide (4-27):

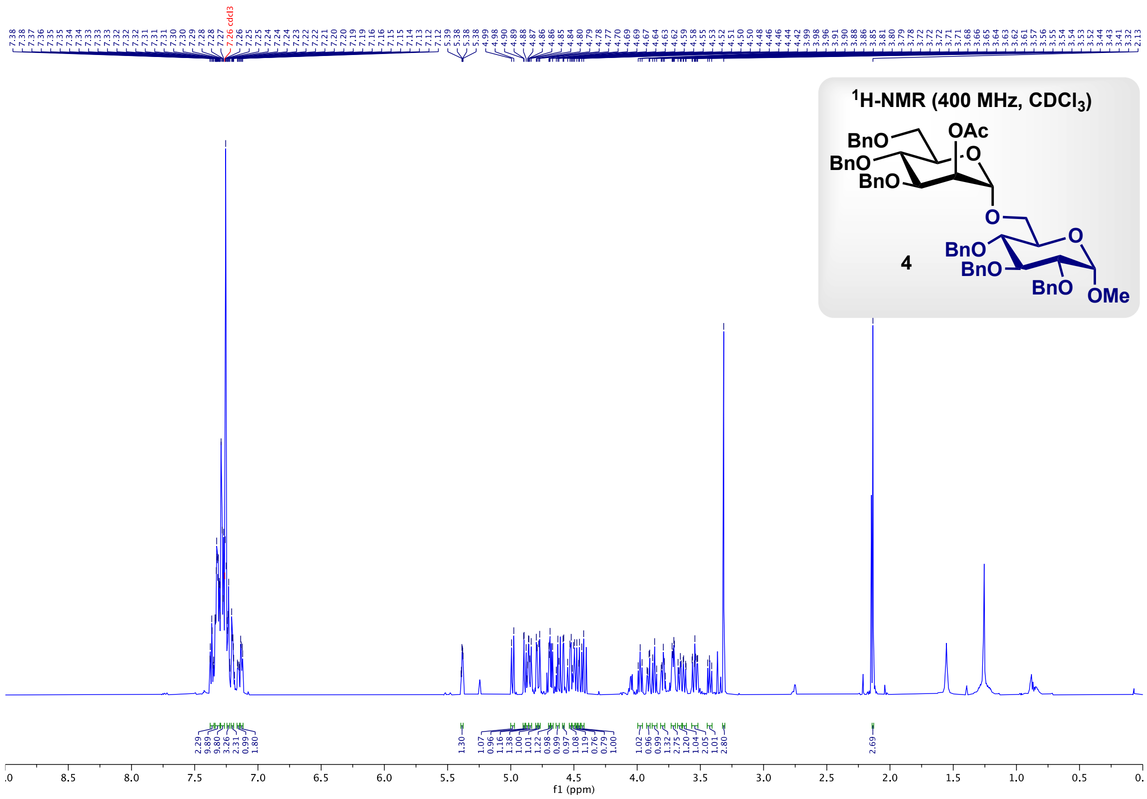

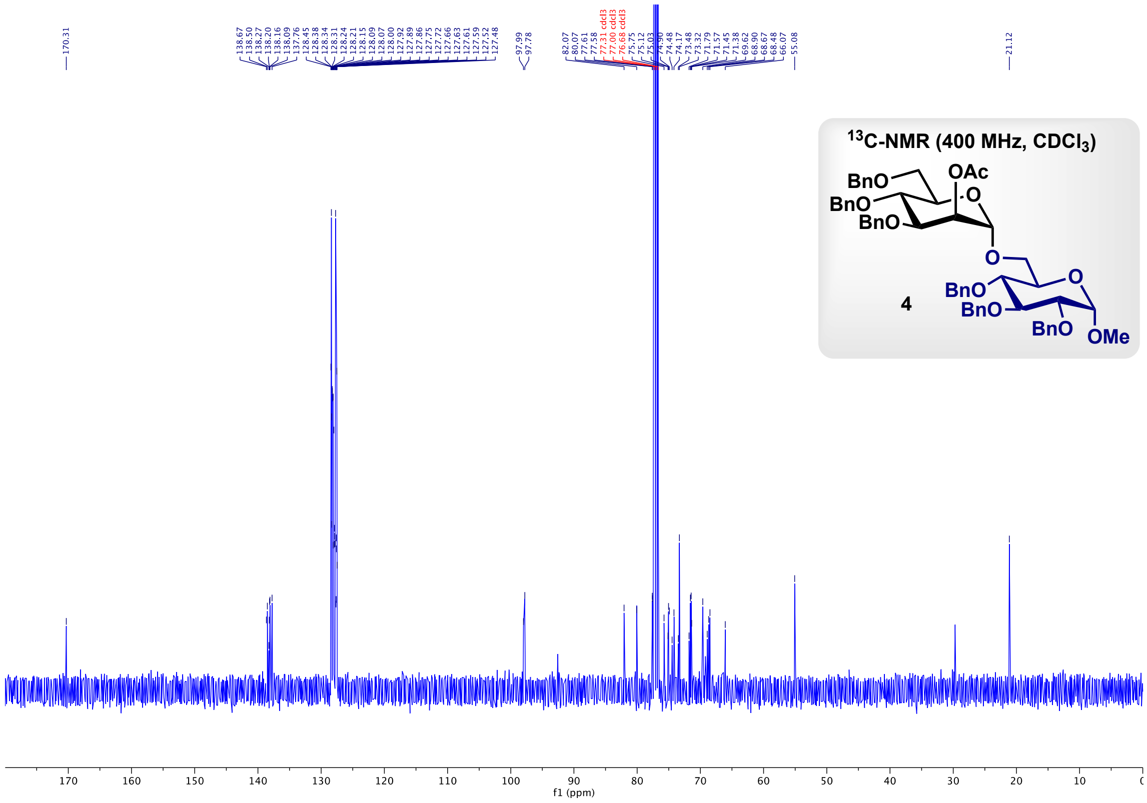

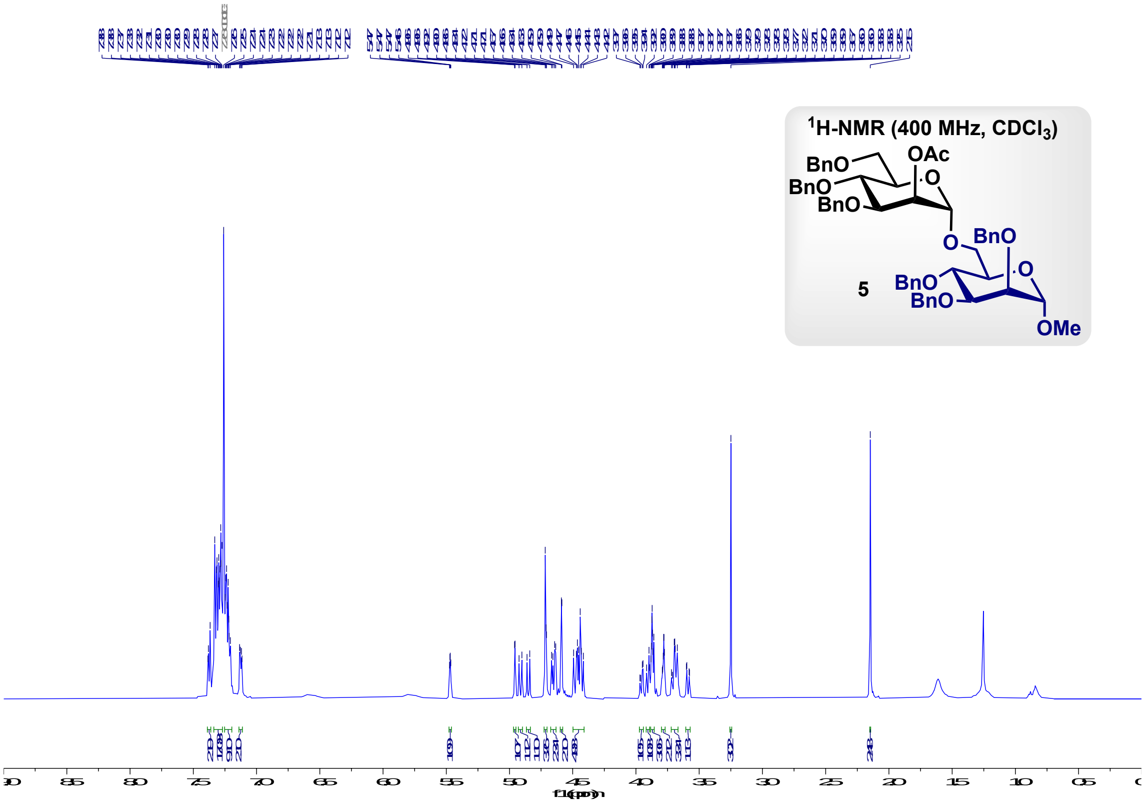
1H-NMR (400 MHz, CDCl3)
BnO
OAc
BnO
O
BnO
O
BnO
5
BnO
O
BnO
OMe
f1 (ppm)

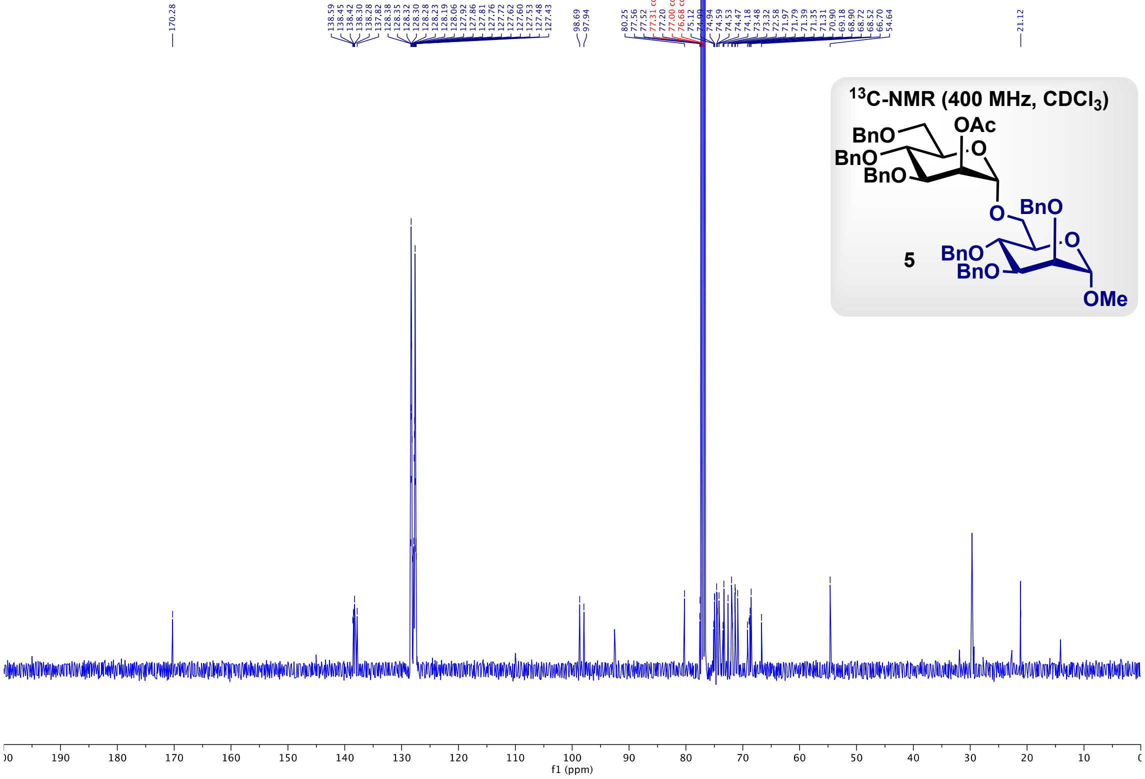
13C-NMR (400 MHz, CDCl3)
BnO
OAc
BnO
O
BnO
O
BnO
5
BnO
O
BnO
OMe
190
180
170
160
150
140
130
120
110
100
90
80
70
60
50
40
30
20
10
f1 (ppm)

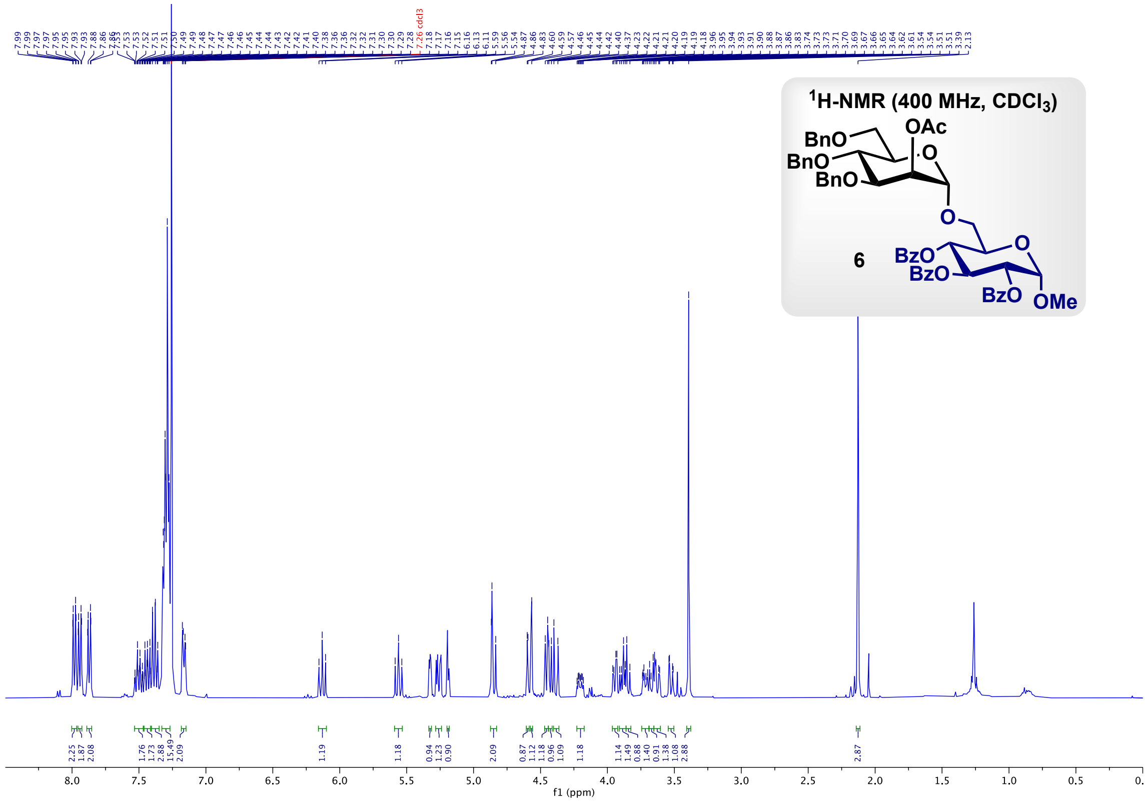

1H-NMR (400 MHz, CDCl3)
OAc
BnO
BnO
BnO
O
O
O
6
BzO
BzO
BzO
OMe
f1 (ppm)

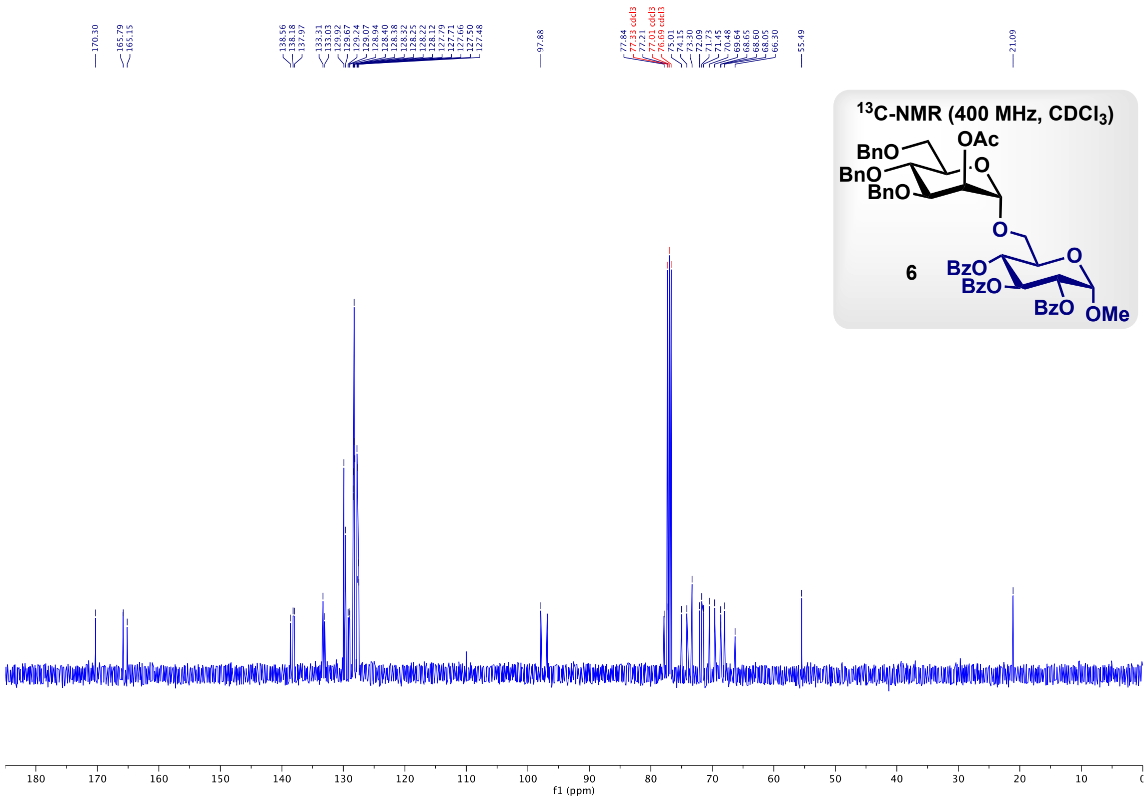

13C-NMR (400 MHz, CDCl3)
OAc
BnO
BnO
BnO
O
O
O
6
BzO
BzO
BzO
OMe
f1 (ppm)

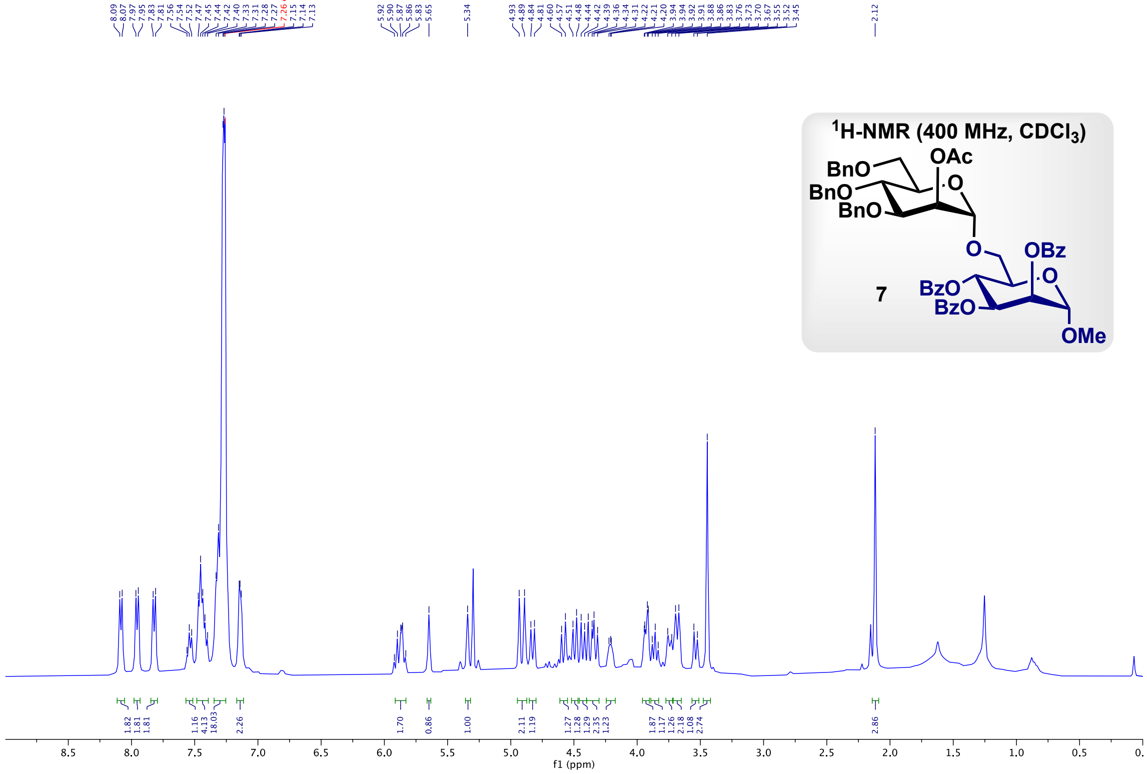
1H-NMR (400 MHz, CDCl3)
OAc
BnO
BnO
BnO
O
O
OBz
O
7
BzO
BzO
OMe
f1 (ppm)

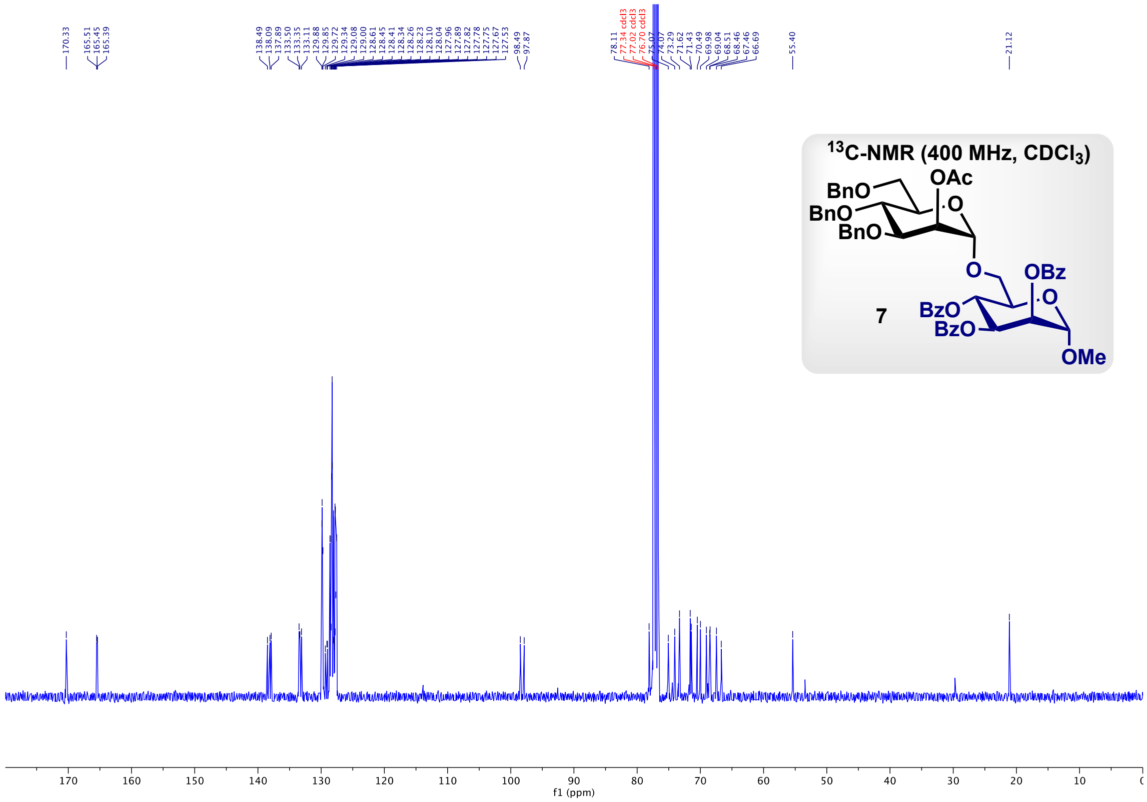
13C-NMR (400 MHz, CDCl3)
OAc
BnO
BnO
BnO
O
O
OBz
O
7
BzO
BzO
OMe
f1 (ppm)

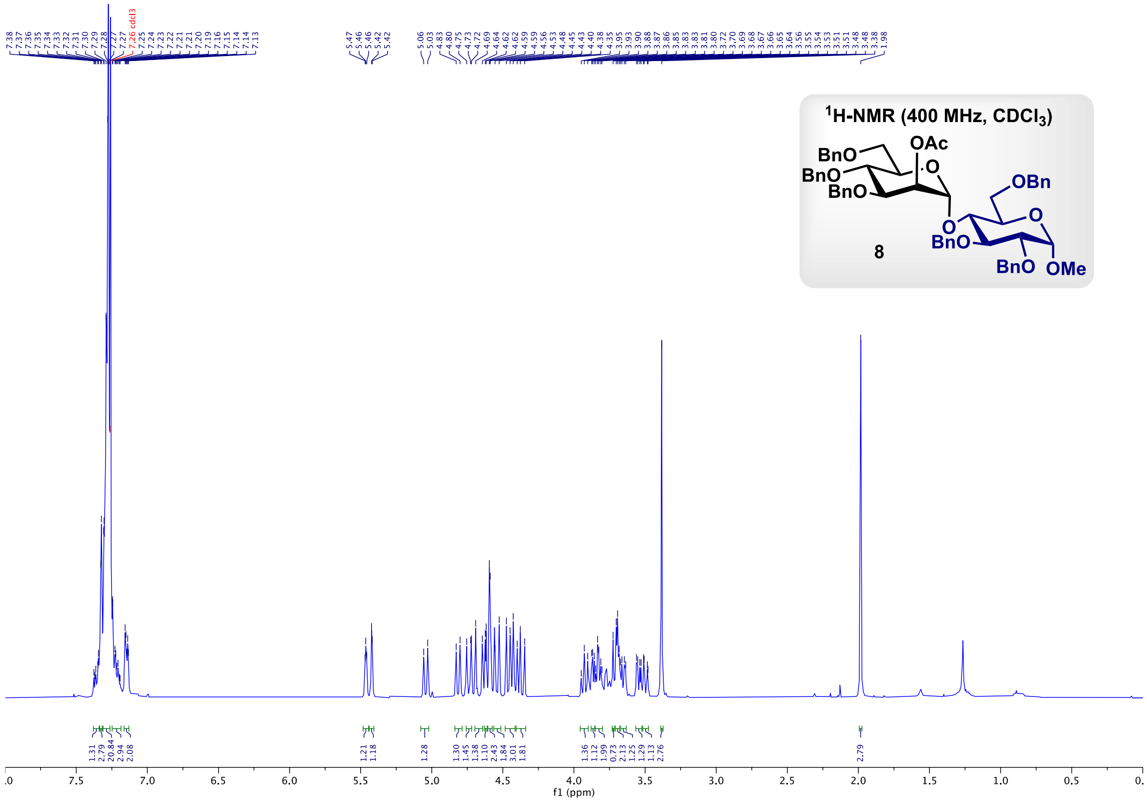

^{1}H-NMR (400 MHz, CDCl$_3$)
OAc
BnO
BnO
BnO
O
OBn
O
O
BnO
8
BnO
OMe
f1 (ppm)

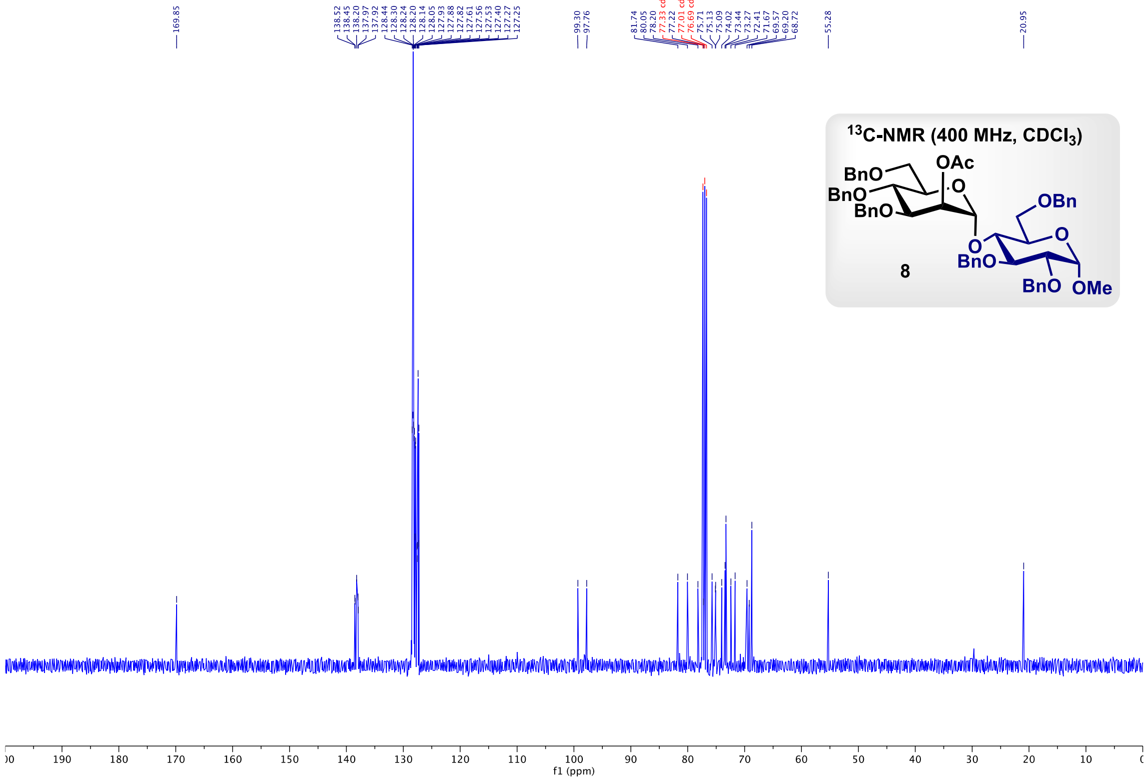

^{13}C-NMR (400 MHz, CDCl$_3$)
OAc
BnO
BnO
BnO
O
OBn
O
O
BnO
8
BnO
OMe
f1 (ppm)

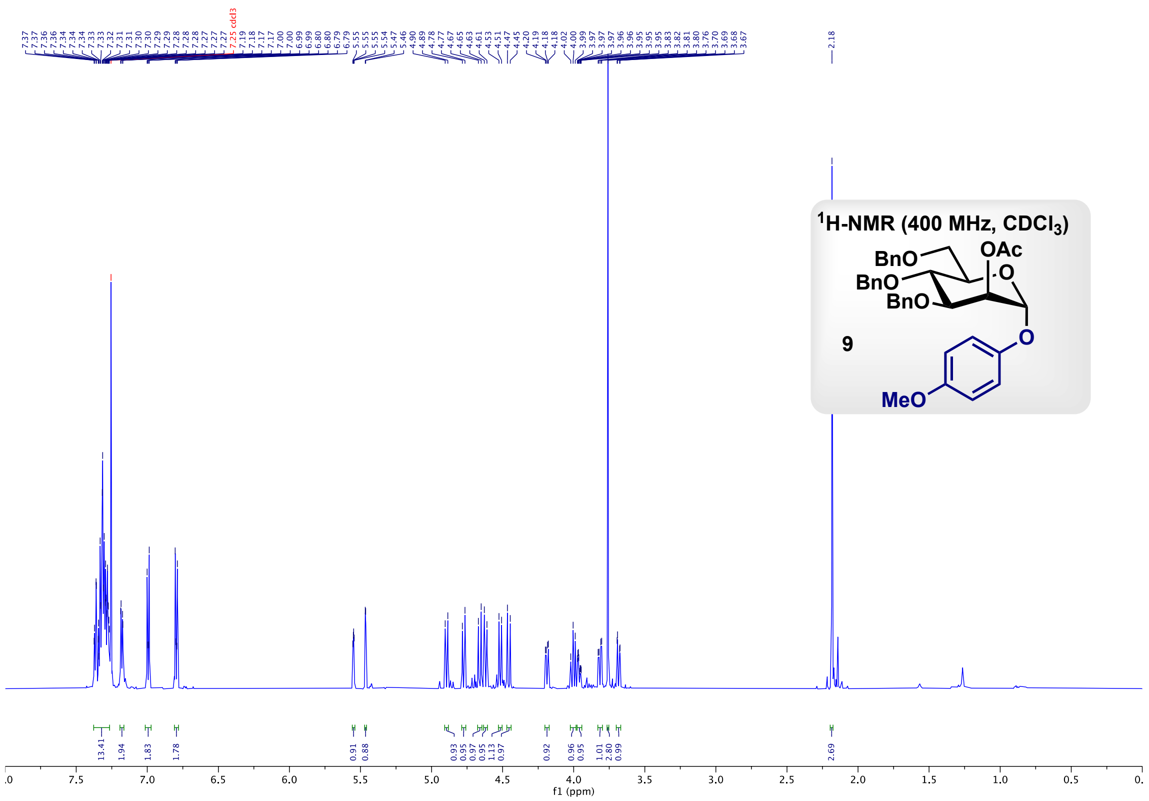

1H-NMR (400 MHz, CDCl3)
BnO
BnO
BnO
OAc
O
O
9
MeO
f1 (ppm)

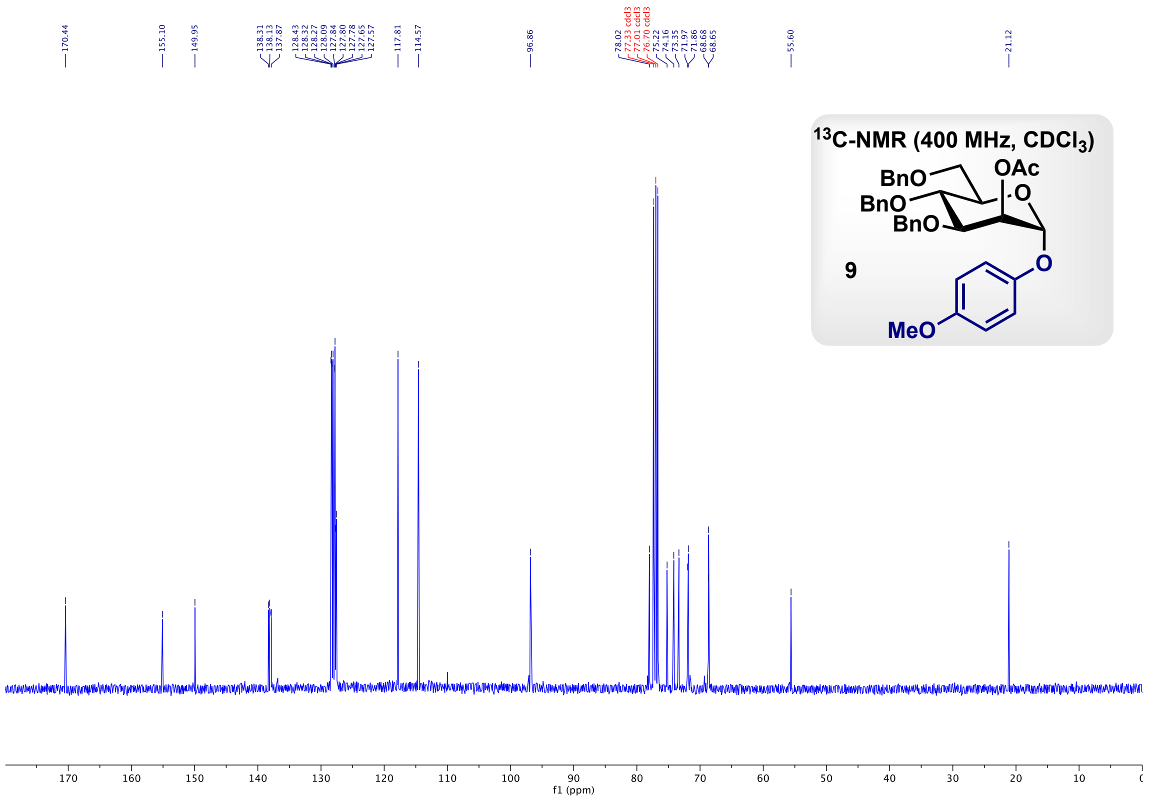

13C-NMR (400 MHz, CDCl3)
BnO
BnO
BnO
OAc
O
O
9
MeO
f1 (ppm)

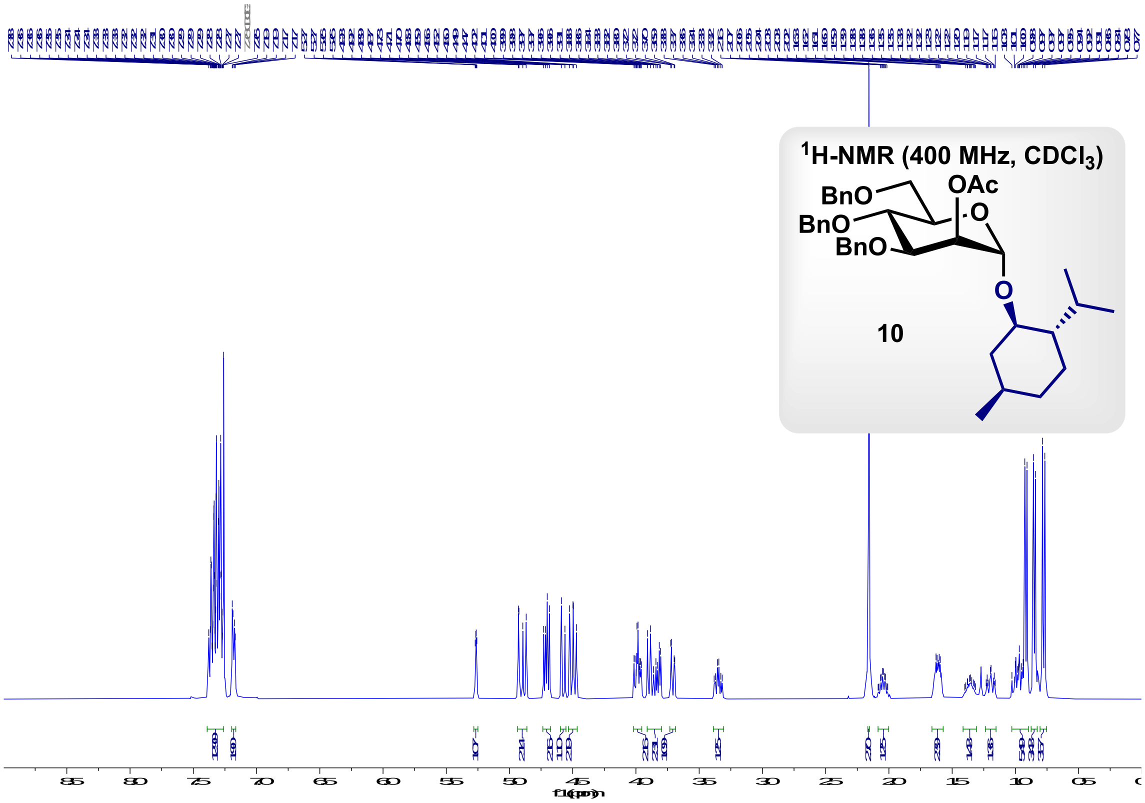

1H-NMR (400 MHz, CDCl3)
BnO
BnO
BnO
OAc
O
O
10
f1 (ppm)

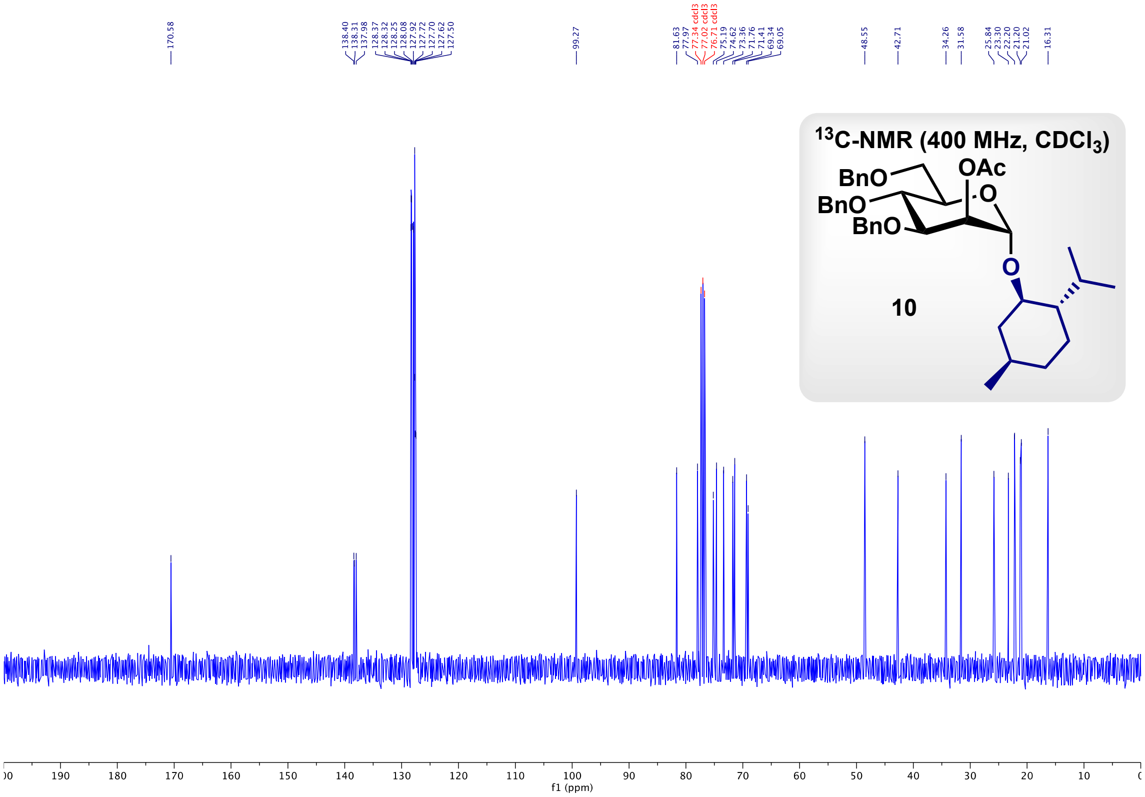

170.58
138.40
138.31
137.98
128.37
128.32
128.25
128.08
127.92
127.72
127.70
127.62
127.50
99.27
81.63
77.97
77.34 cdcl3
77.02 cdcl3
76.71 cdcl3
75.19
74.62
73.36
71.76
71.41
69.34
69.05
48.55
42.71
34.26
31.58
25.84
23.30
22.20
21.20
21.02
16.31
13C-NMR (400 MHz, CDCl3)
BnO
BnO
BnO
OAc
O
O
10
f1 (ppm)

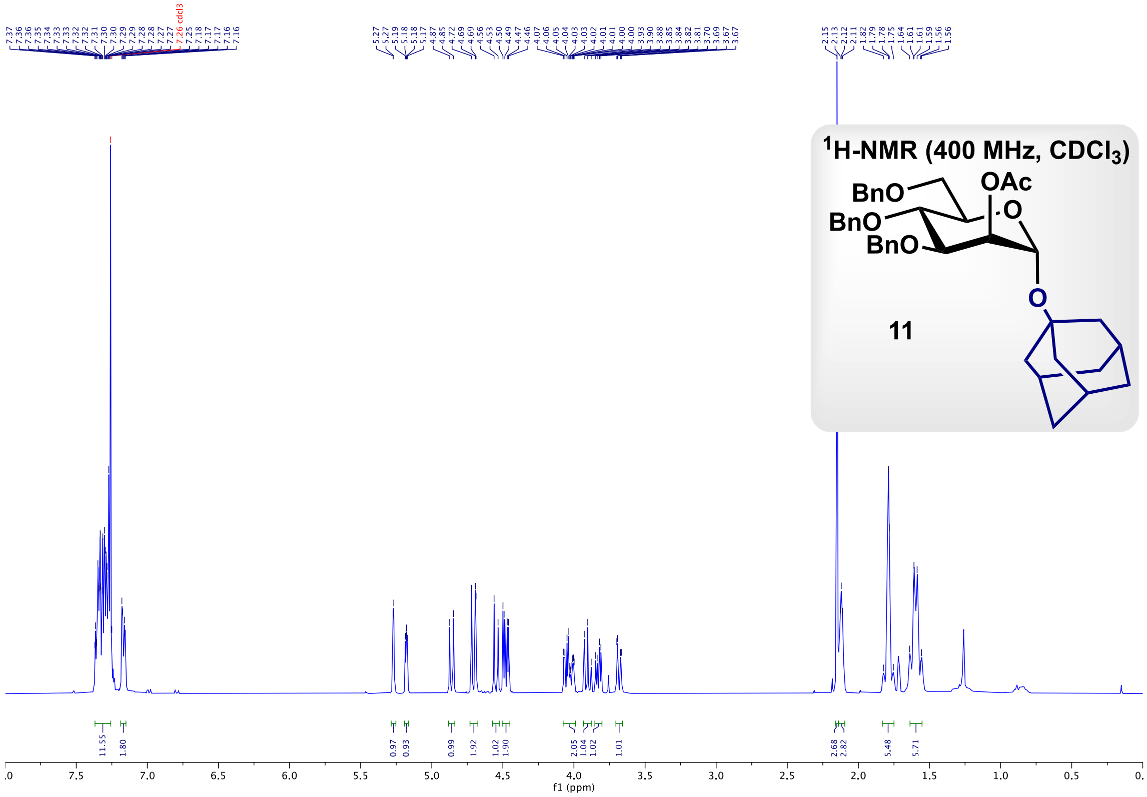
1H-NMR (400 MHz, CDCl3)
BnO
BnO
BnO
OAc
O
O
11
f1 (ppm)

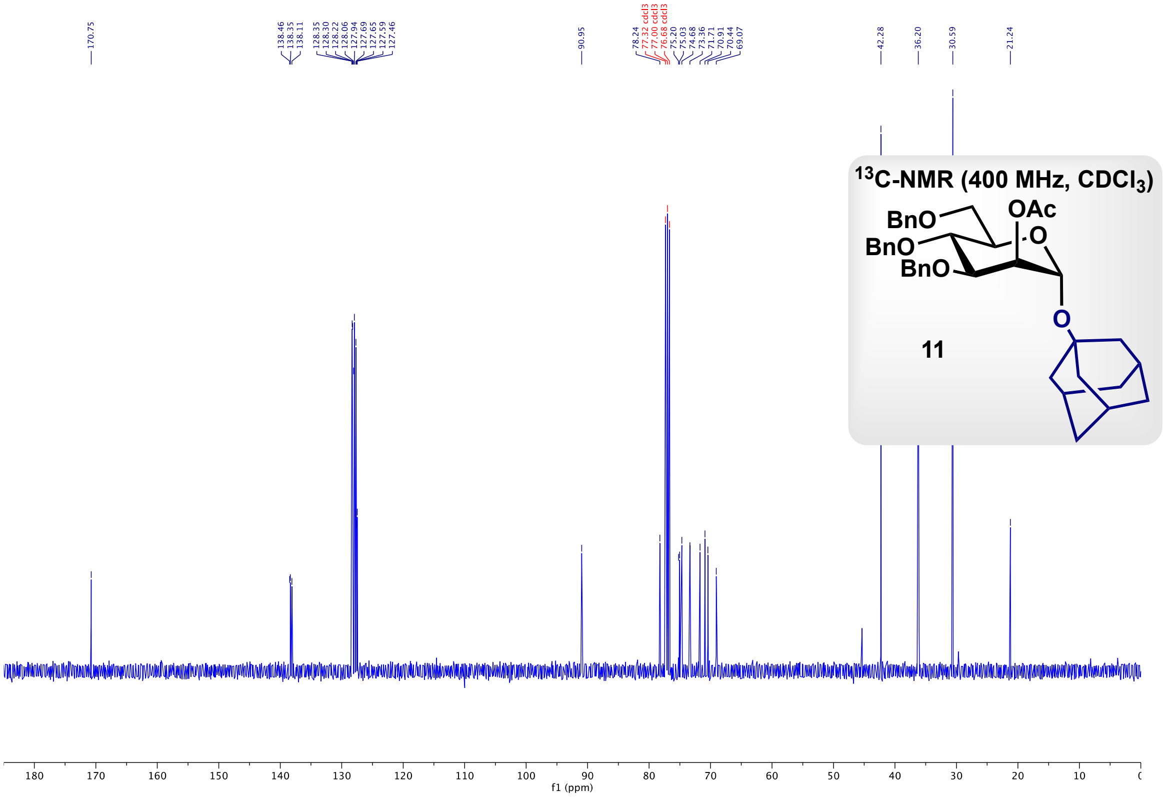
13C-NMR (400 MHz, CDCl3)
BnO
BnO
BnO
OAc
O
O
11
f1 (ppm)

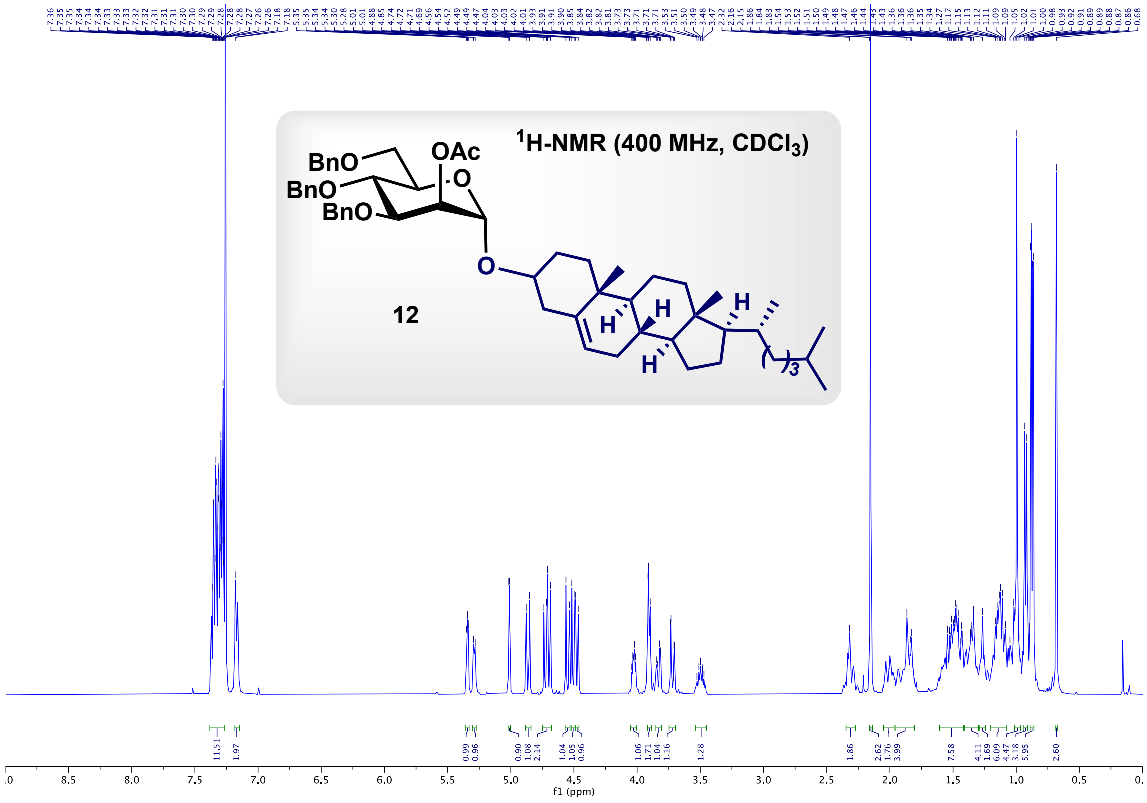
1H-NMR (400 MHz, CDCl3)
BnO
BnO
BnO
OAc
O
O
12
H
H
H
H
3
f1 (ppm)

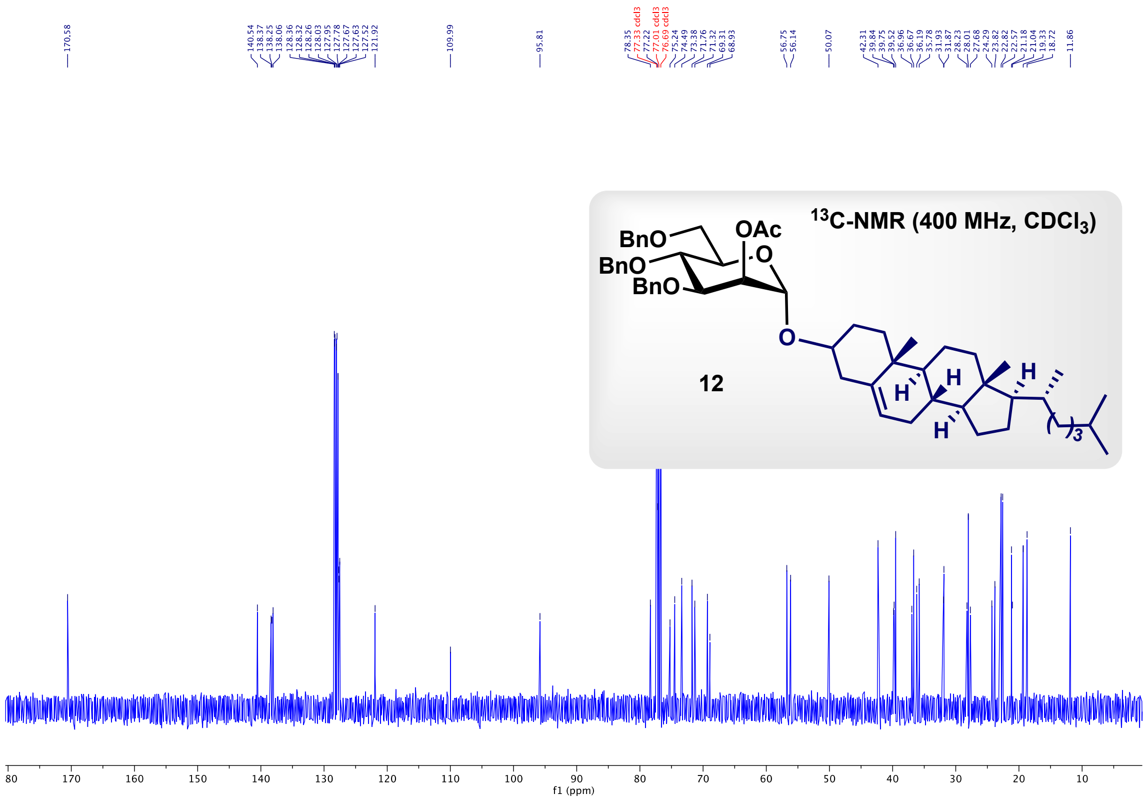
13C-NMR (400 MHz, CDCl3)
BnO
BnO
BnO
OAc
O
O
12
H
H
H
H
3
f1 (ppm)

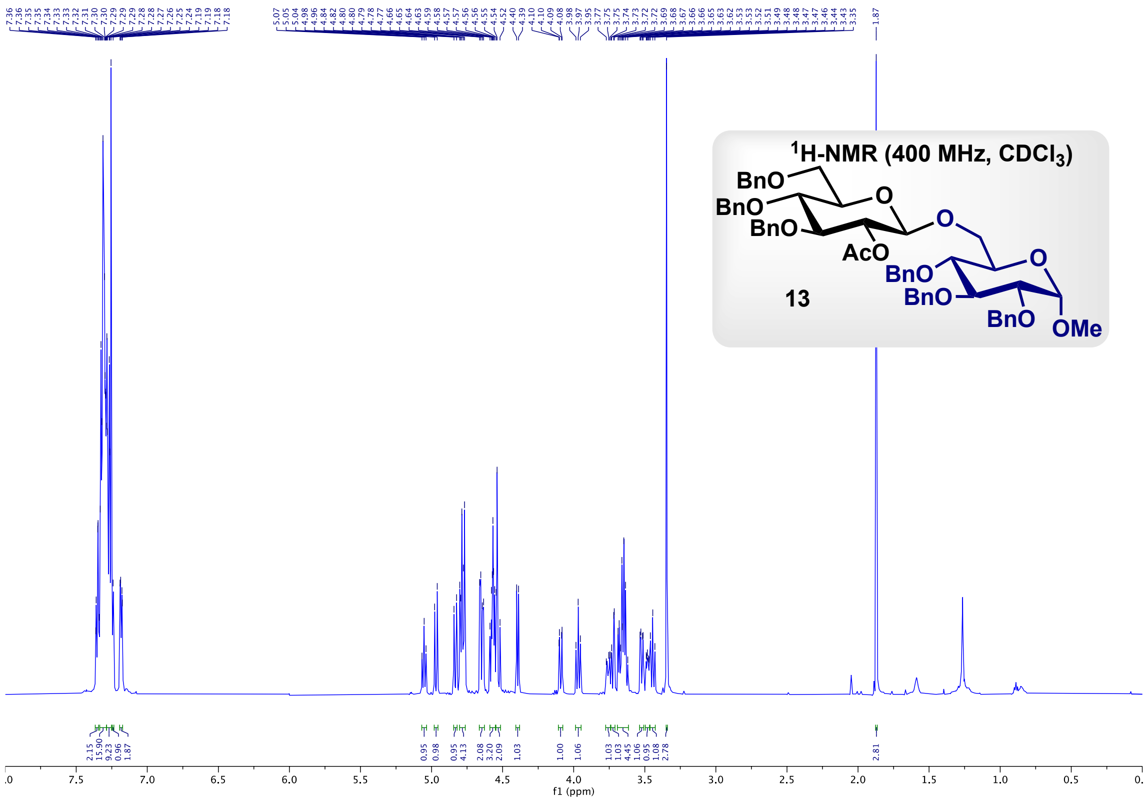

1H-NMR (400 MHz, CDCl3)
BnO
BnO
BnO
O
O
AcO
BnO
BnO
O
BnO
OMe
13
f1 (ppm)

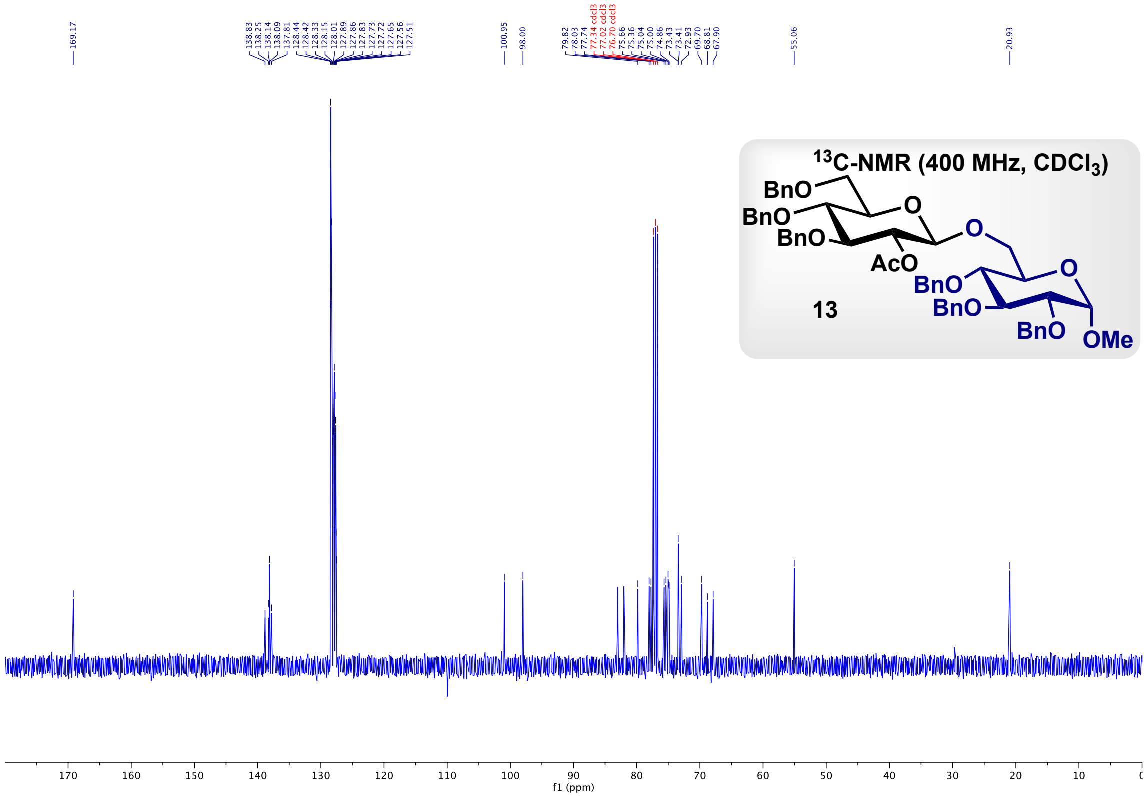

13C-NMR (400 MHz, CDCl3)
BnO
BnO
BnO
O
O
AcO
BnO
BnO
O
BnO
OMe
13
f1 (ppm)

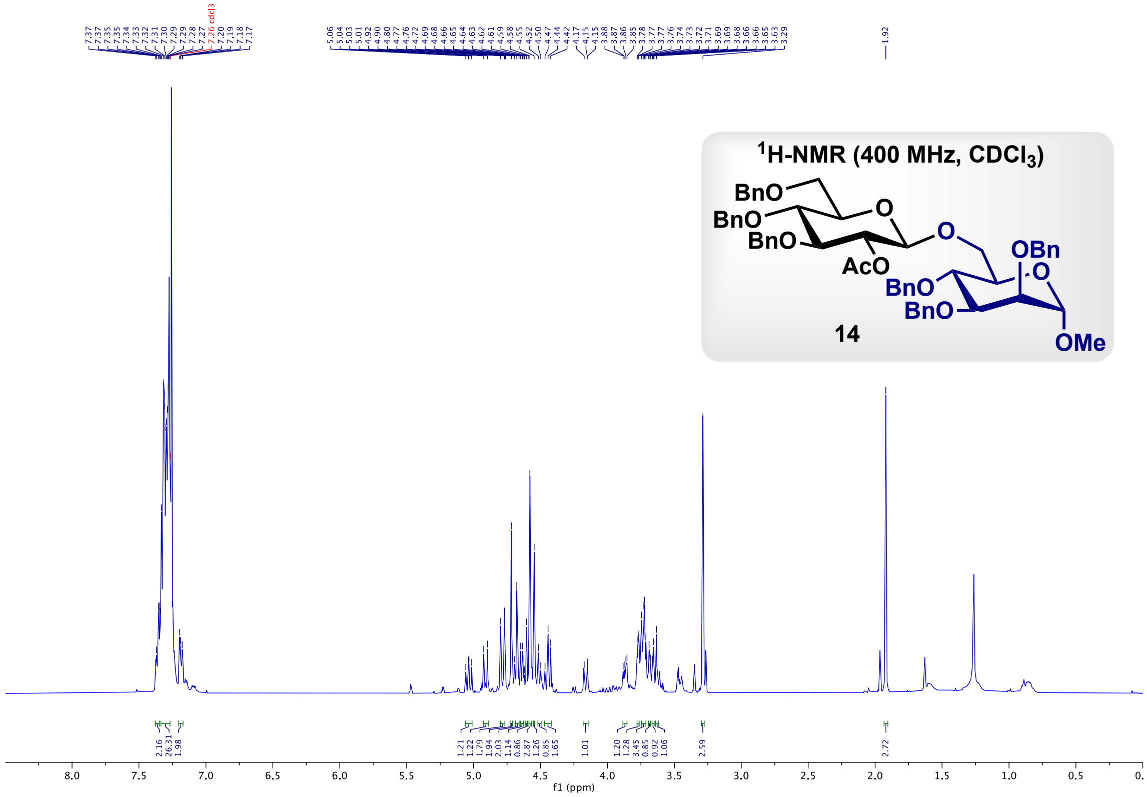
^{1}H-NMR (400 MHz, $CDCl_3$)
BnO
BnO
BnO
O
O
AcO
OBn
O
BnO
BnO
OMe
14
f1 (ppm)

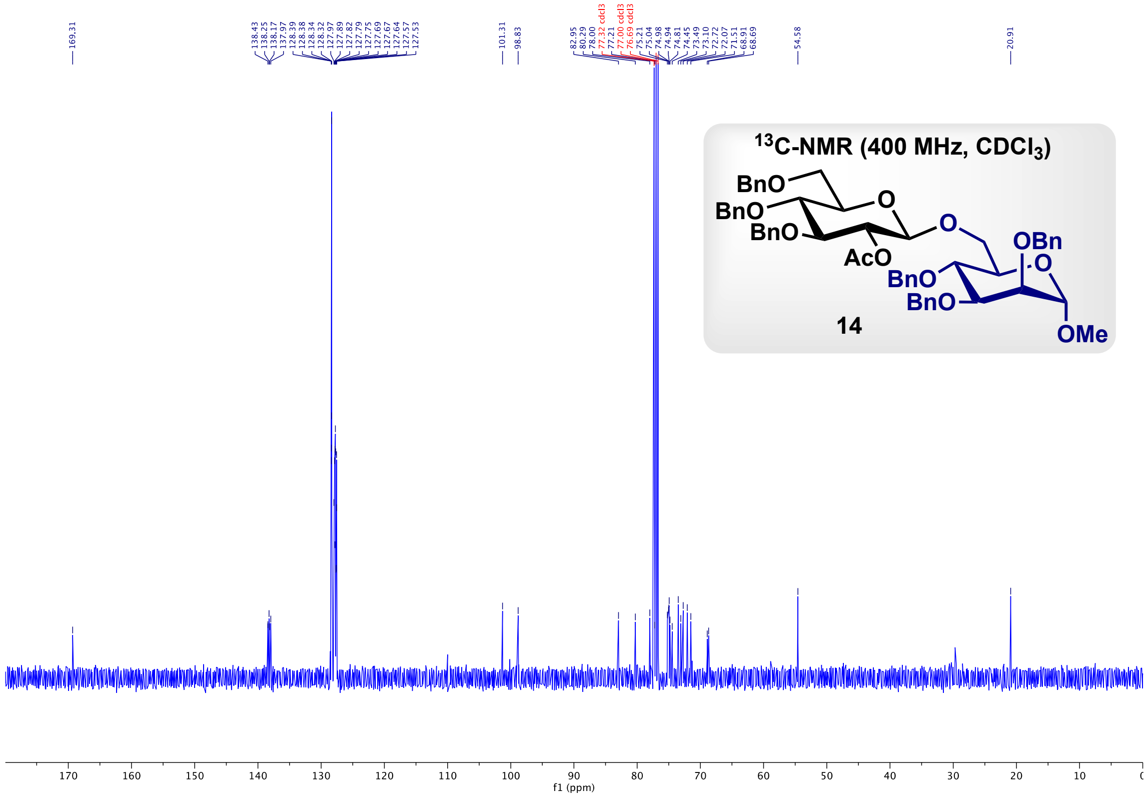
^{13}C-NMR (400 MHz, $CDCl_3$)
BnO
BnO
BnO
O
O
AcO
OBn
O
BnO
BnO
OMe
14
f1 (ppm)

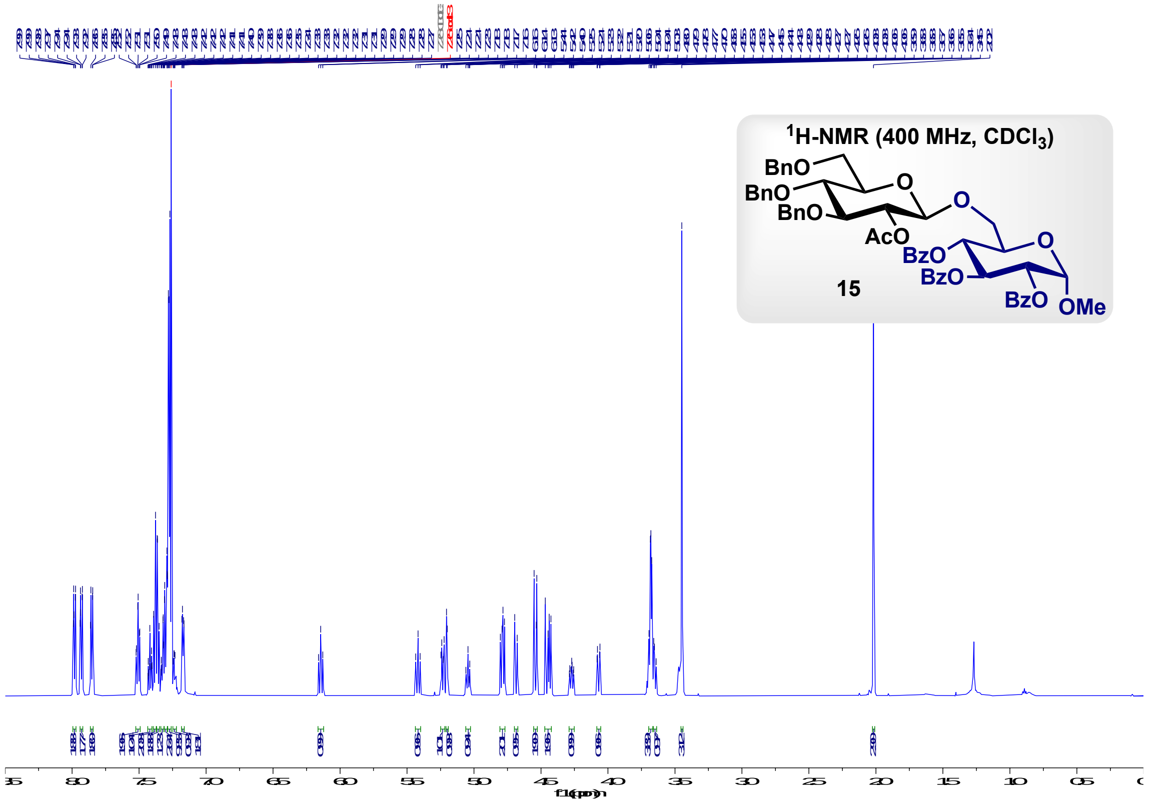

1H-NMR (400 MHz, CDCl3)
BnO
BnO
BnO
O
AcO
O
BzO
BzO
O
BzO
OMe
15
f1 (ppm)

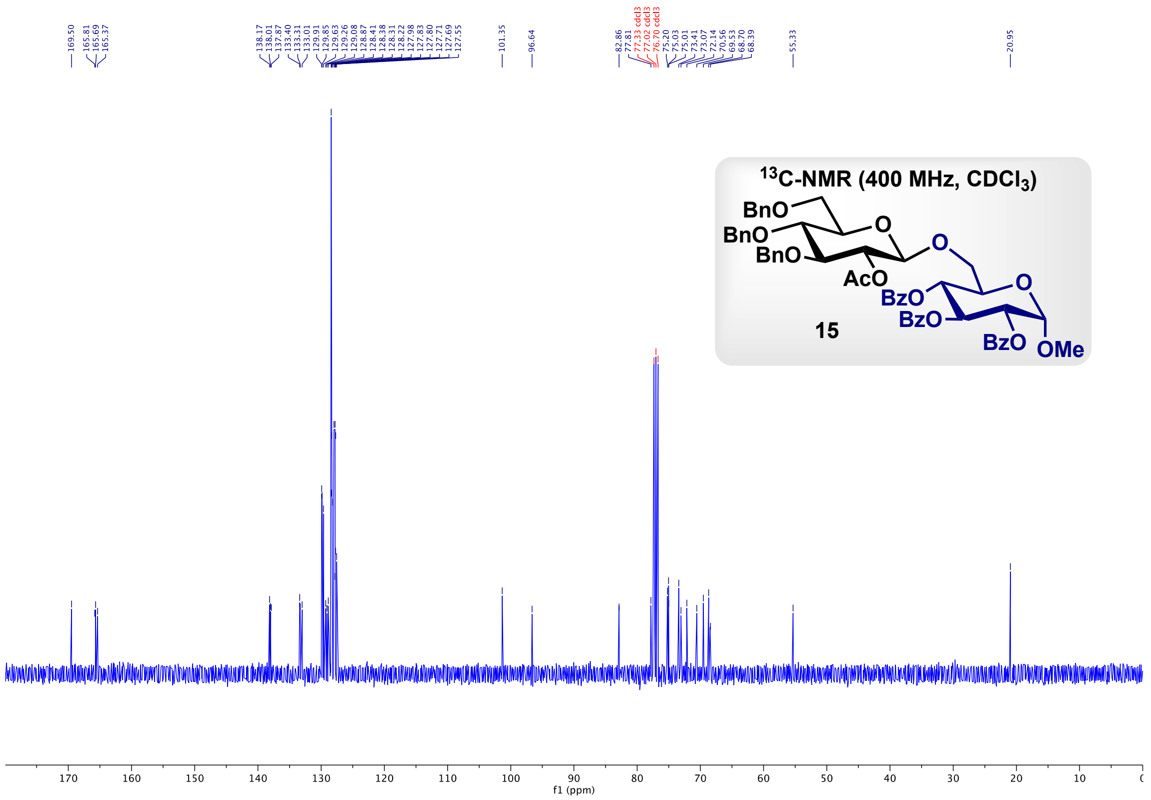

169.50
165.81
165.69
165.37
138.17
138.01
137.87
133.40
133.31
133.01
129.91
129.85
129.63
129.26
129.08
128.87
128.41
128.38
128.31
128.22
127.98
127.83
127.80
127.71
127.69
127.55
101.35
96.64
82.86
77.81
77.33 cdcl3
77.02 cdcl3
76.70 cdcl3
75.20
75.03
75.01
73.41
73.07
72.14
70.56
69.53
68.70
68.39
55.33
20.95
13C-NMR (400 MHz, CDCl3)
BnO
BnO
BnO
O
AcO
O
BzO
BzO
O
BzO
OMe
15
f1 (ppm)

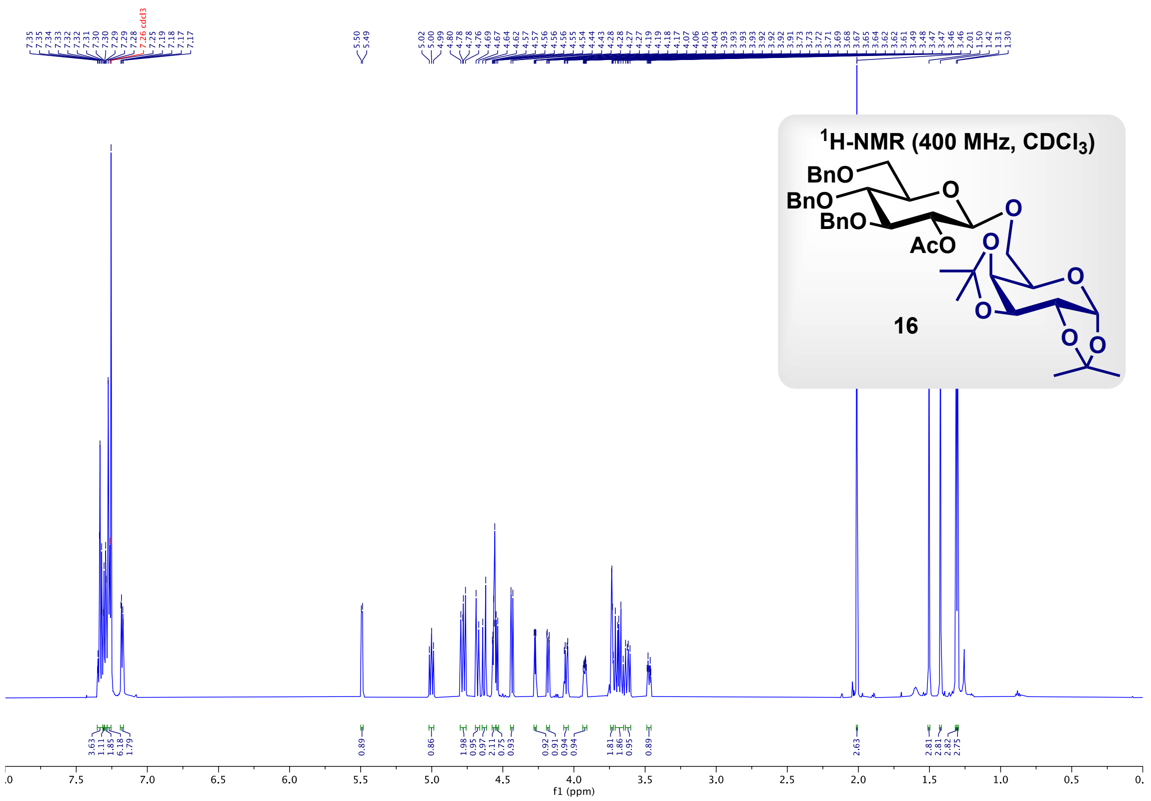

1H-NMR (400 MHz, CDCl3)
BnO
BnO
BnO
AcO
O
16
f1 (ppm)

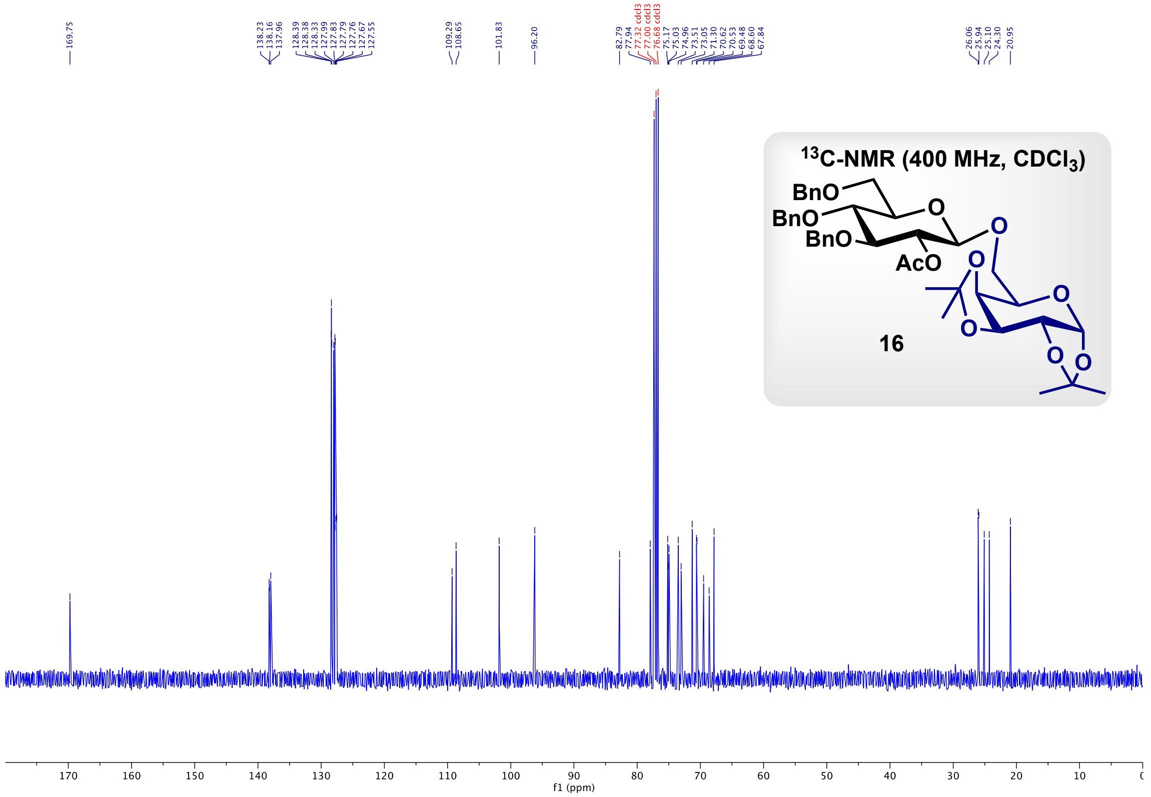

13C-NMR (400 MHz, CDCl3)
BnO
BnO
BnO
AcO
O
16
f1 (ppm)

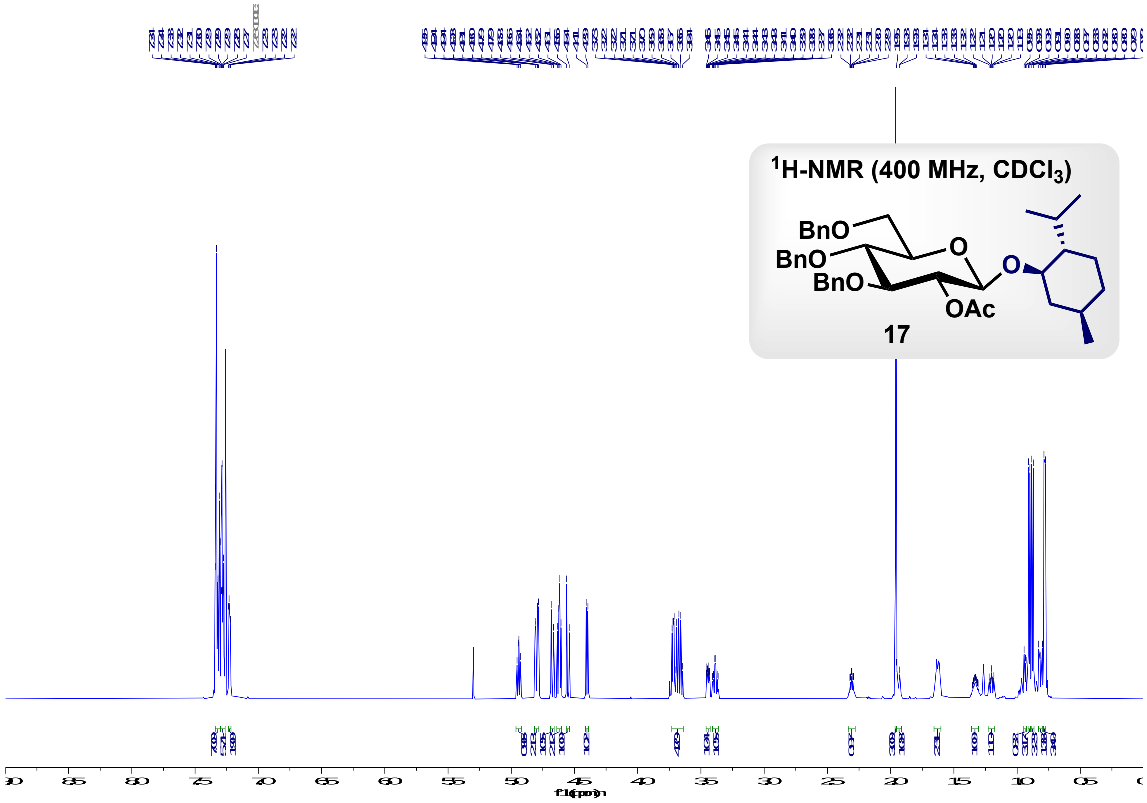
1H-NMR (400 MHz, CDCl3)
BnO
BnO
BnO
O
O
OAc
17
f1 (ppm)

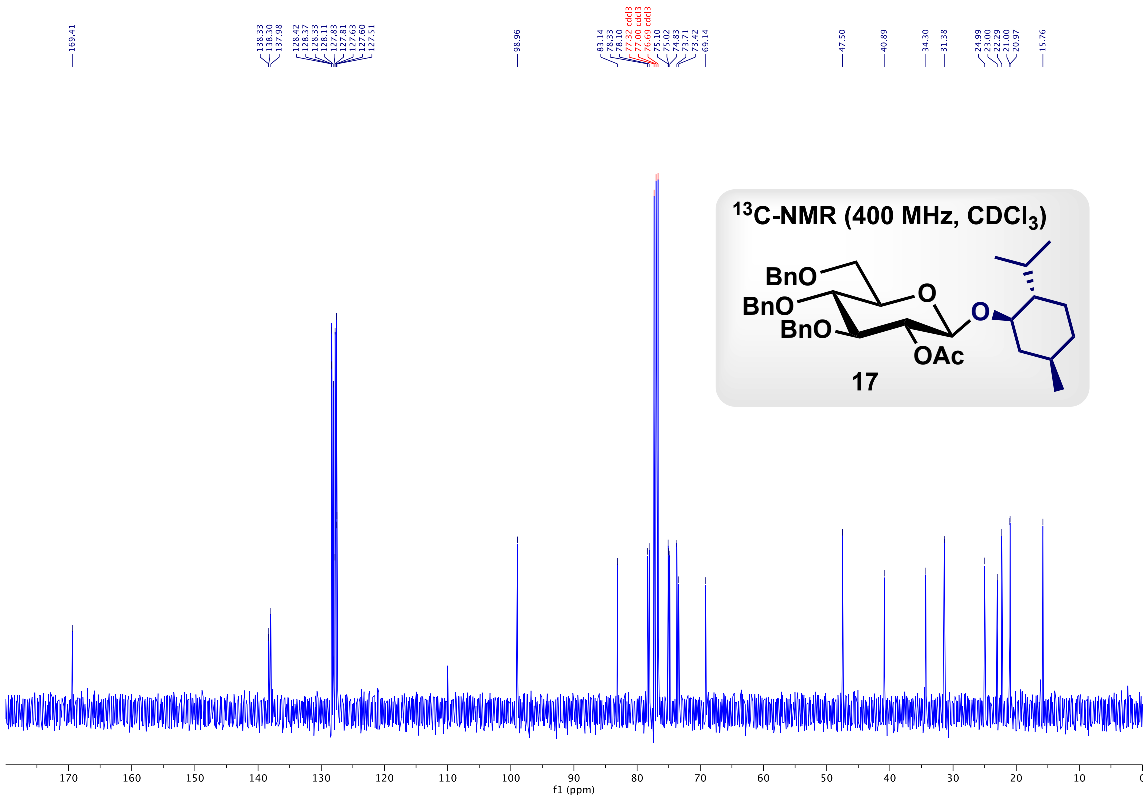
169.41
138.33
138.30
137.98
128.42
128.37
128.33
128.11
127.83
127.81
127.63
127.60
127.51
98.96
83.14
78.33
78.10
77.32 cdcl3
77.00 cdcl3
76.69 cdcl3
75.10
75.02
74.83
73.71
73.42
69.14
47.50
40.89
34.30
31.38
24.99
23.00
22.29
21.00
20.97
15.76
13C-NMR (400 MHz, CDCl3)
BnO
BnO
BnO
O
O
OAc
17
f1 (ppm)

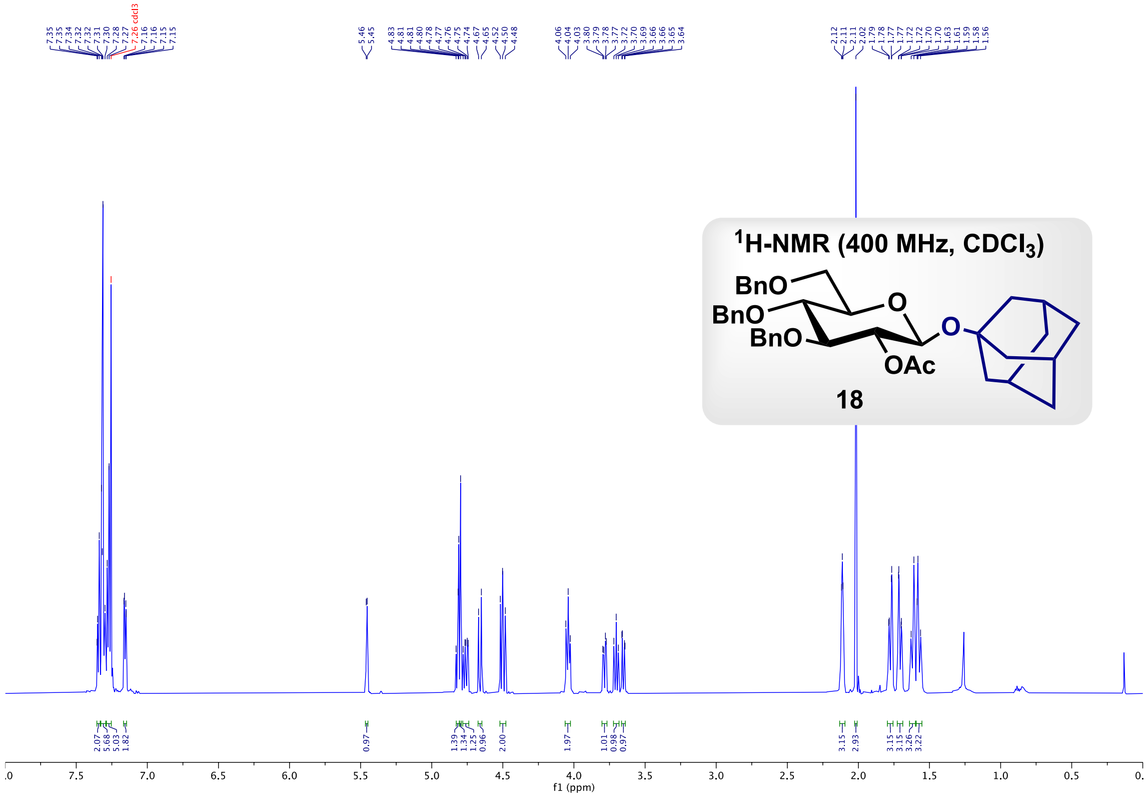

^{1}H-NMR (400 MHz, CDCl$_3$)
BnO
BnO
BnO
O
O
OAc
18
f1 (ppm)

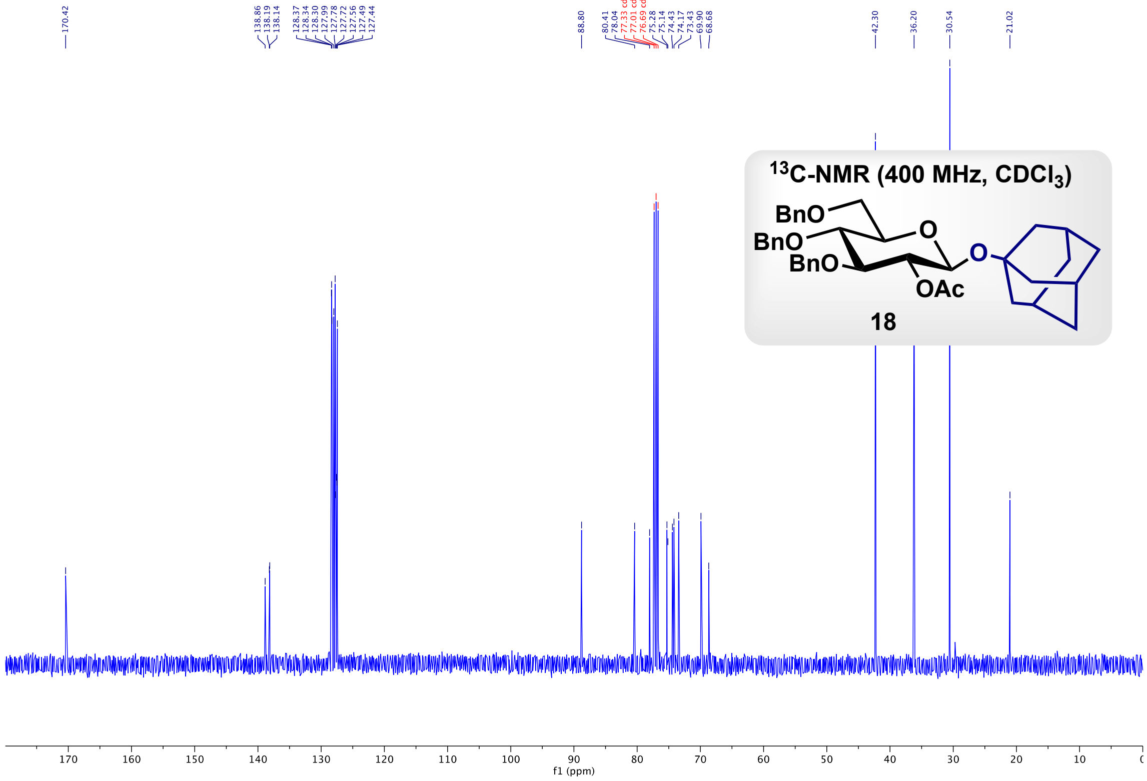

^{13}C-NMR (400 MHz, CDCl$_3$)
BnO
BnO
BnO
O
O
OAc
18
f1 (ppm)

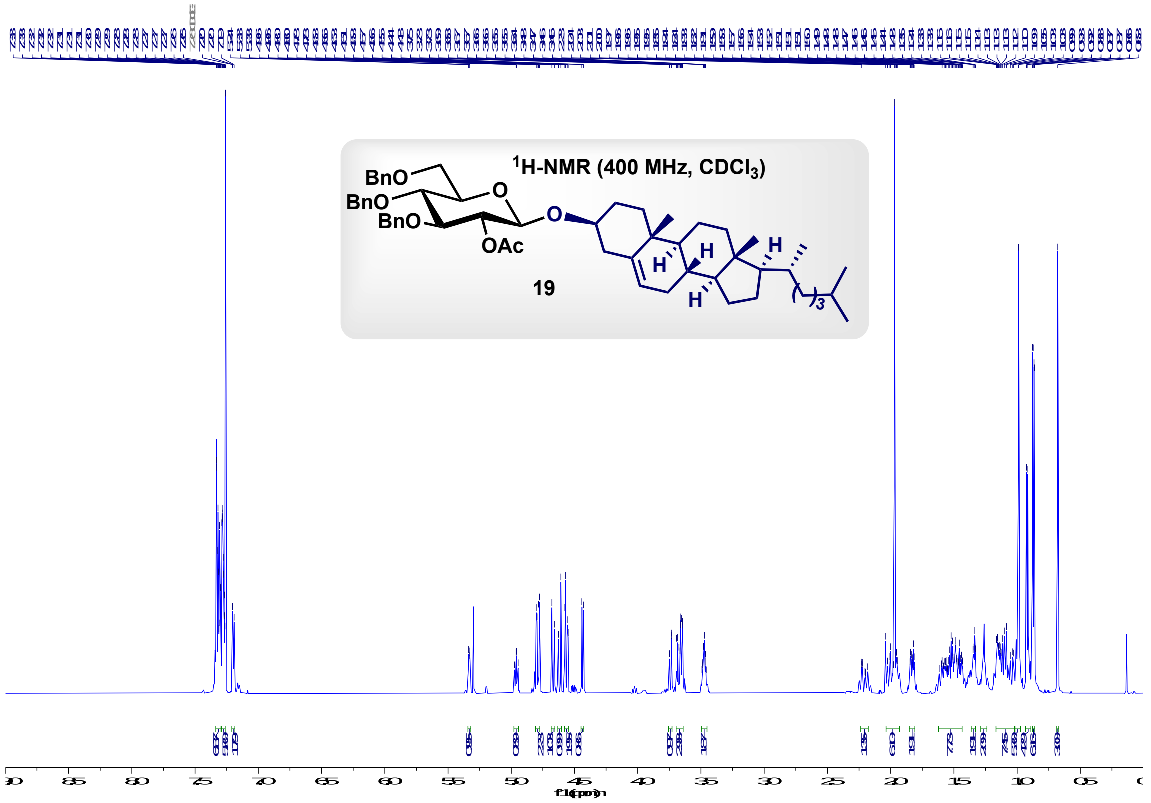
1H-NMR (400 MHz, CDCl3)
BnO
BnO
BnO
O
O
OAc
19
f1 (ppm)

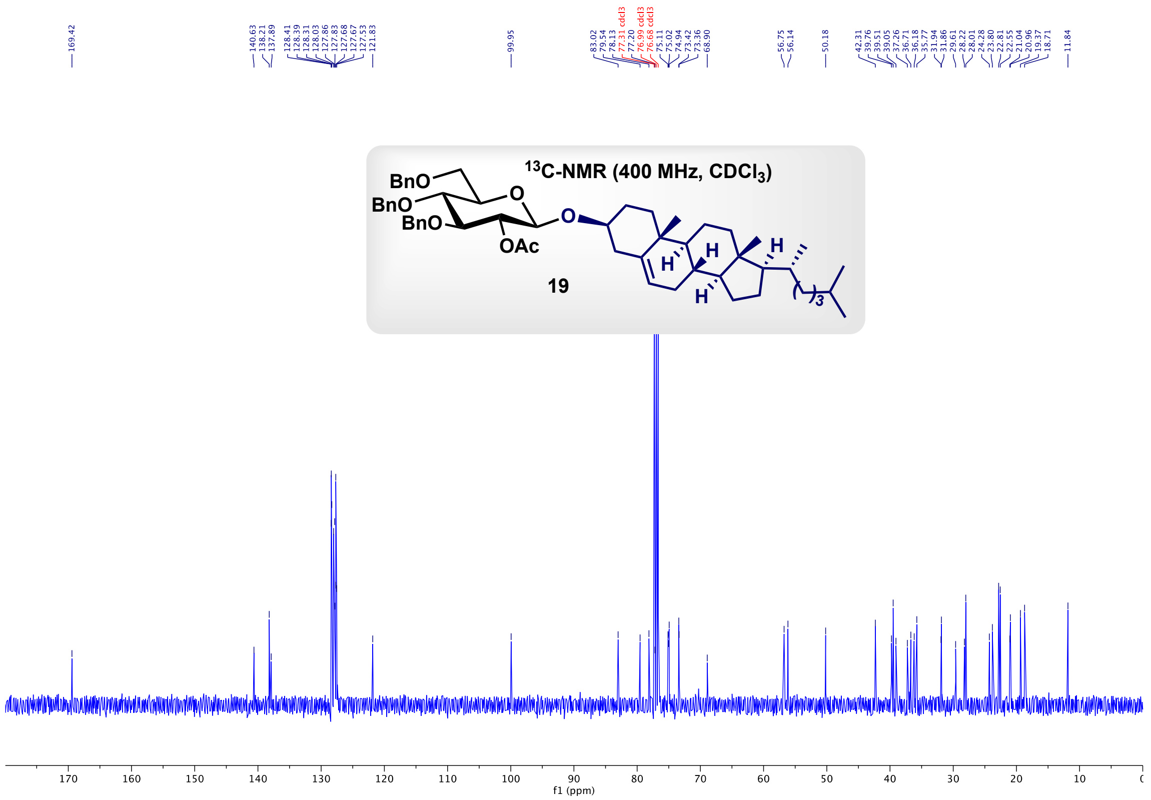
13C-NMR (400 MHz, CDCl3)
BnO
BnO
BnO
O
O
OAc
19
169.42
140.63
138.21
137.89
121.83
99.95
83.02
79.54
78.13
68.90
56.75
56.14
50.18
11.84
f1 (ppm)

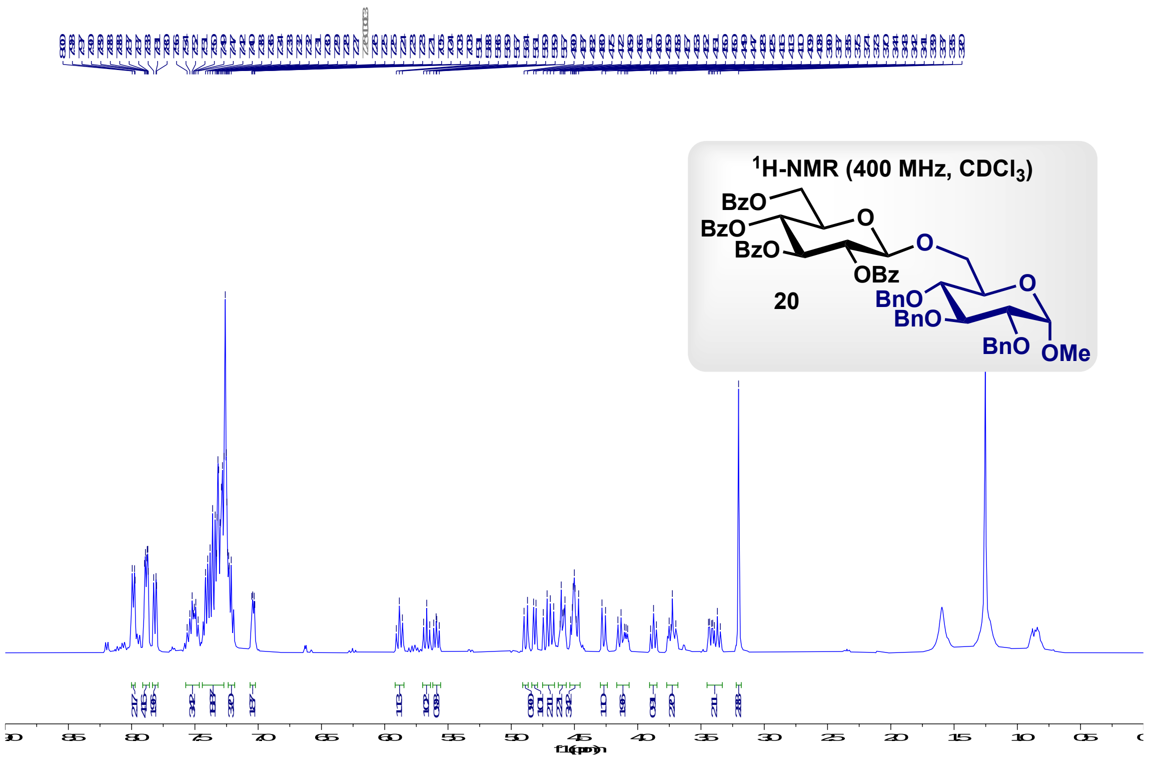

1H-NMR (400 MHz, CDCl3)
BzO
BzO
BzO
O
O
OBz
20
BnO
BnO
O
BnO
OMe
f1 (ppm)

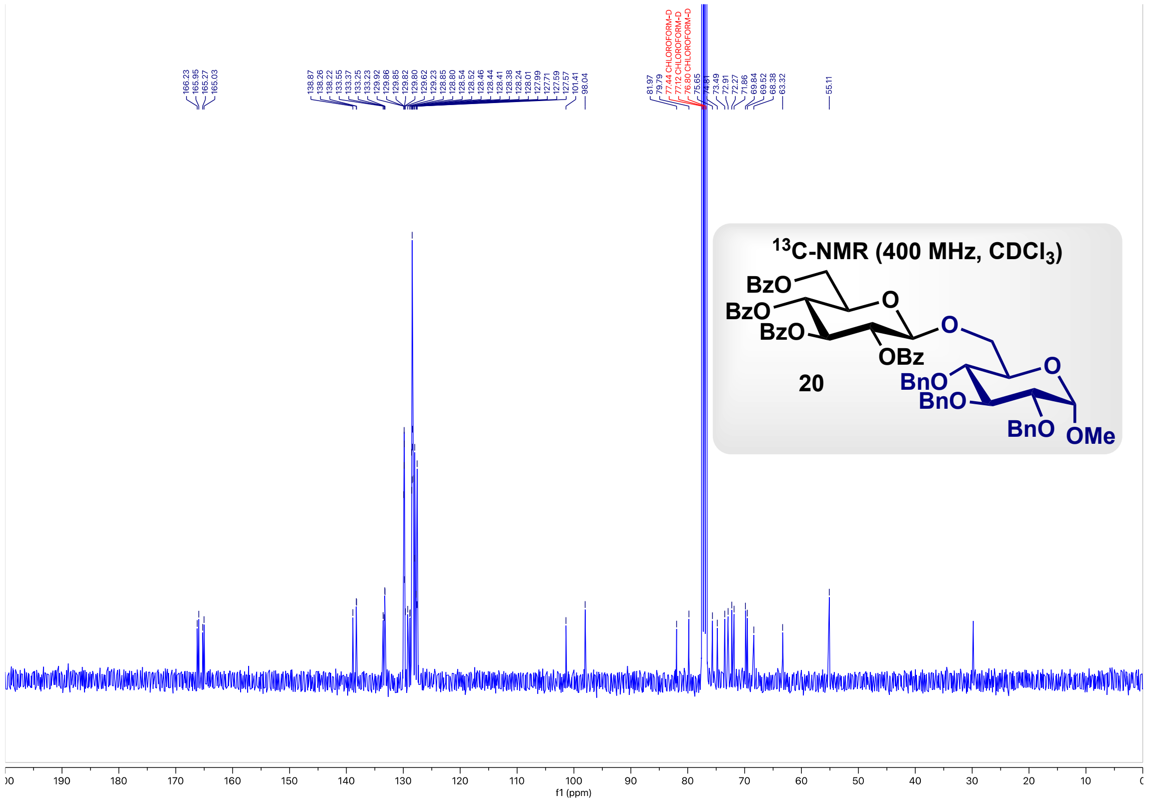

166.23
165.95
165.27
165.03
138.87
138.26
138.22
133.55
133.37
133.25
133.23
129.92
129.86
129.85
129.82
129.80
129.62
129.23
128.85
128.80
128.54
128.52
128.46
128.44
128.41
128.38
128.24
128.01
127.99
127.71
127.59
127.57
101.41
98.04
81.97
79.79
77.44 CHLOROFORM-D
77.12 CHLOROFORM-D
76.80 CHLOROFORM-D
75.65
73.49
72.91
72.27
71.86
69.84
69.52
68.38
63.32
55.11
13C-NMR (400 MHz, CDCl3)
BzO
BzO
BzO
O
O
OBz
20
BnO
BnO
O
BnO
OMe
f1 (ppm)

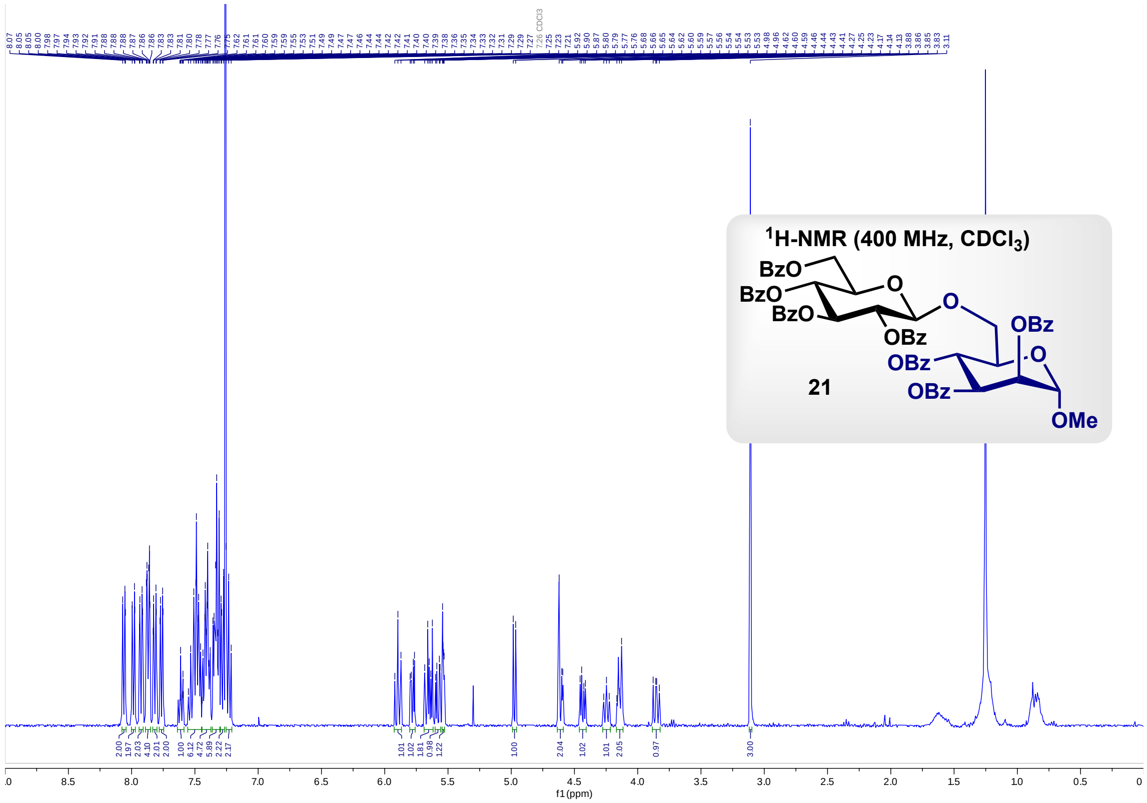

1H-NMR (400 MHz, CDCl3)
BzO
BzO
BzO
O
O
OBz
OBz
O
OBz
OBz
OMe
21
f1 (ppm)

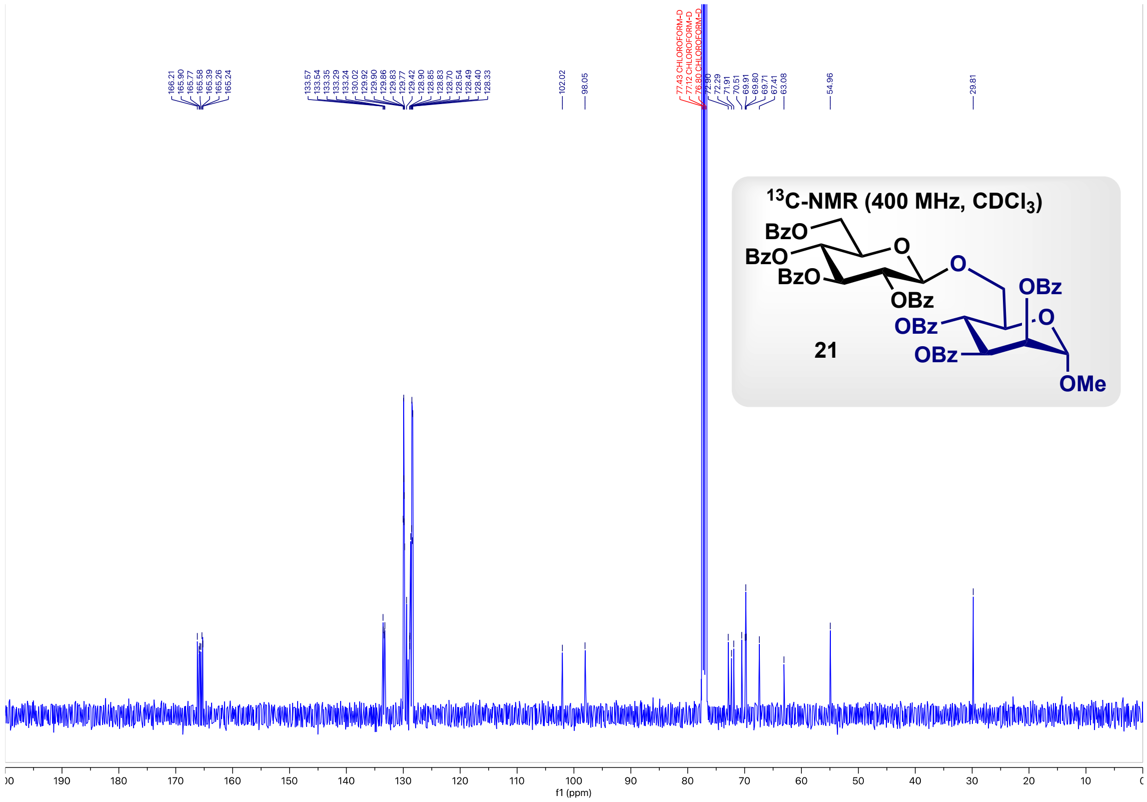

13C-NMR (400 MHz, CDCl3)
BzO
BzO
BzO
O
O
OBz
OBz
O
OBz
OBz
OMe
21
f1 (ppm)

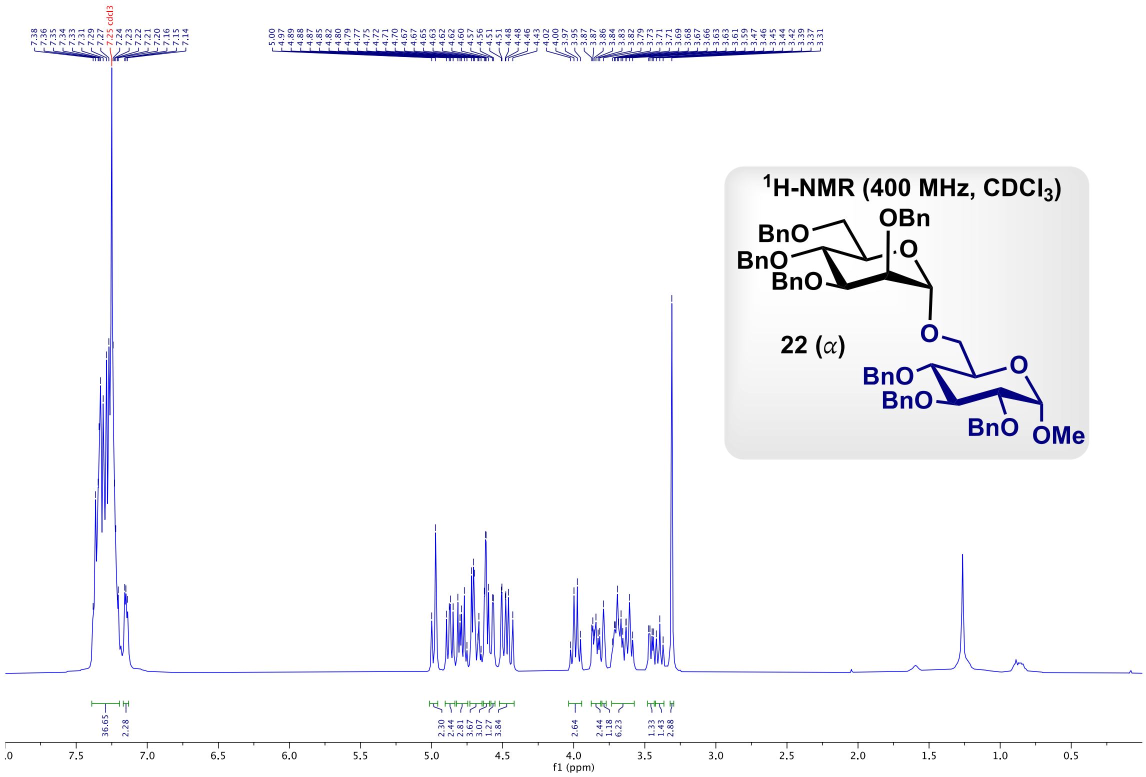
1H-NMR (400 MHz, CDCl3)
BnO
OBn
BnO
O
BnO
22 (α)
O
BnO
O
BnO
BnO
OMe
f1 (ppm)

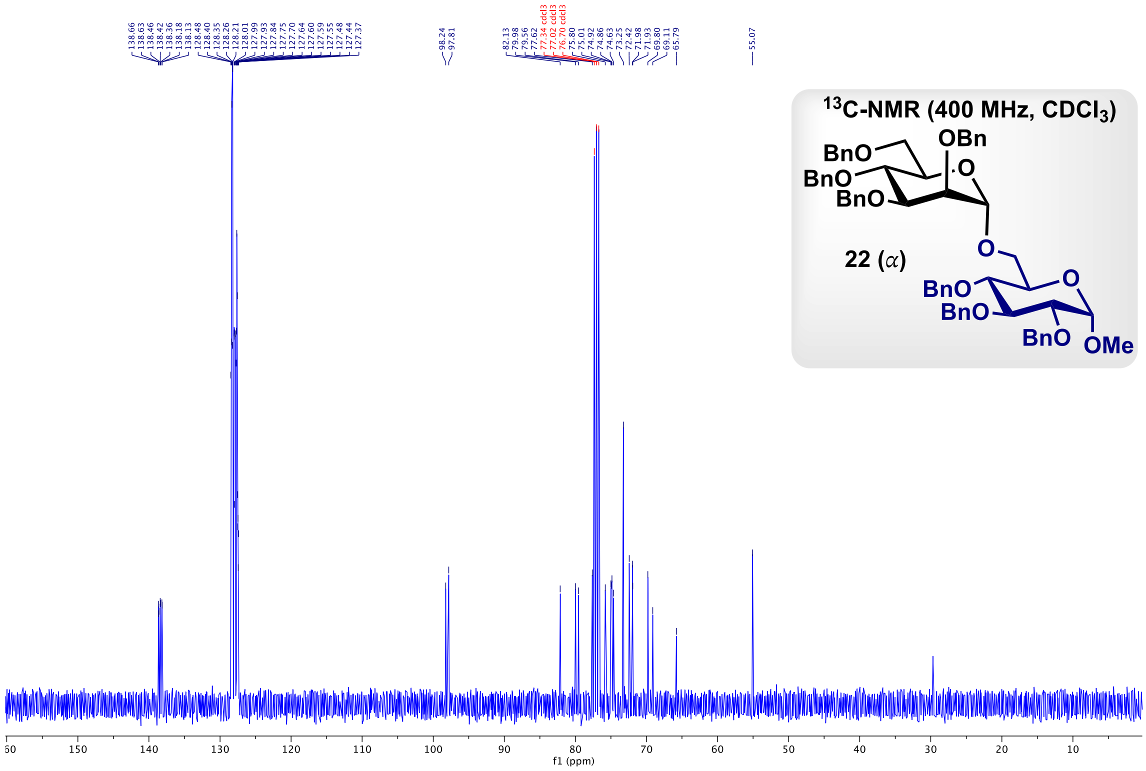
13C-NMR (400 MHz, CDCl3)
BnO
OBn
BnO
O
BnO
22 (α)
O
BnO
O
BnO
BnO
OMe
f1 (ppm)

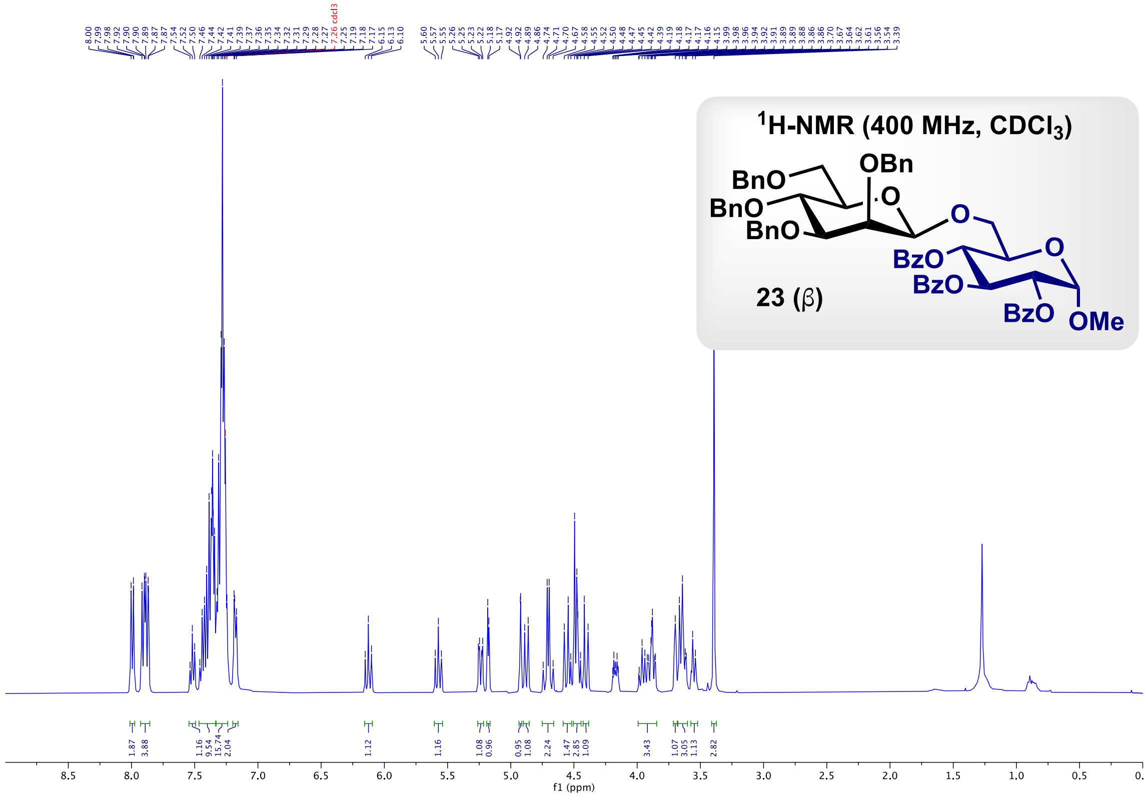
^{1}H-NMR (400 MHz, $CDCl_3$)
BnO
BnO
BnO
OBn
O
O
O
BzO
BzO
BzO
OMe
23 (β)
f1 (ppm)

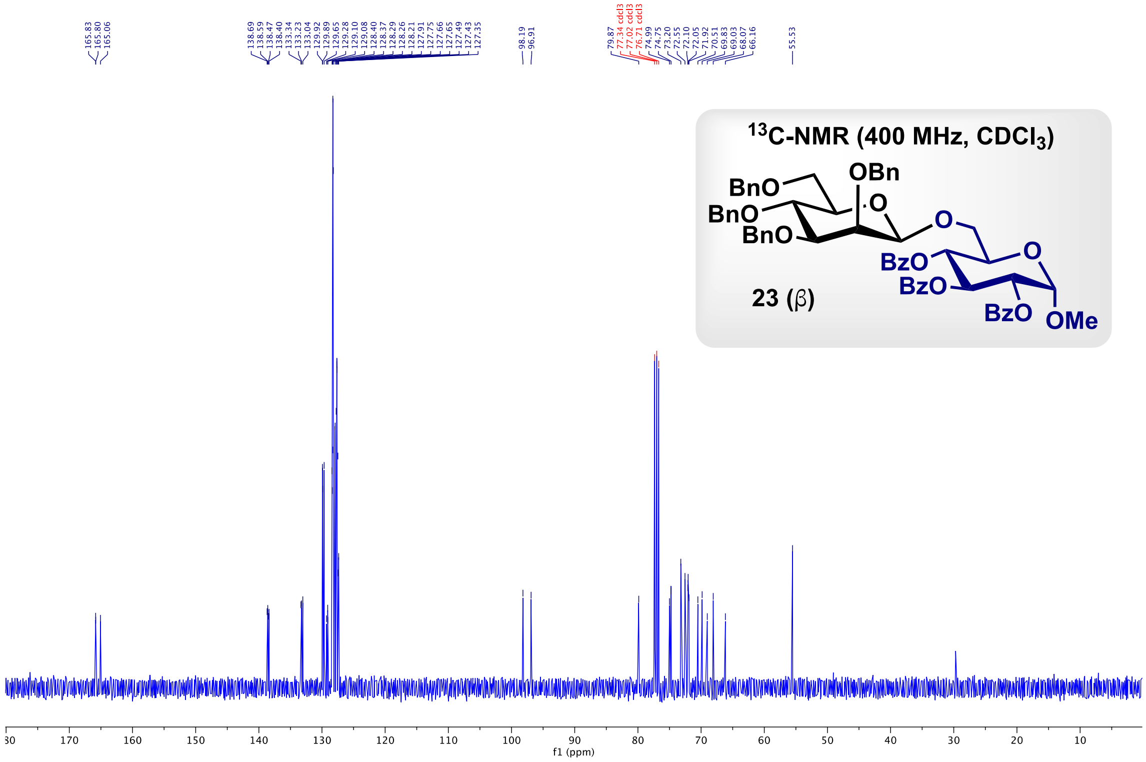
^{13}C-NMR (400 MHz, $CDCl_3$)
BnO
BnO
BnO
OBn
O
O
O
BzO
BzO
BzO
OMe
23 (β)
f1 (ppm)

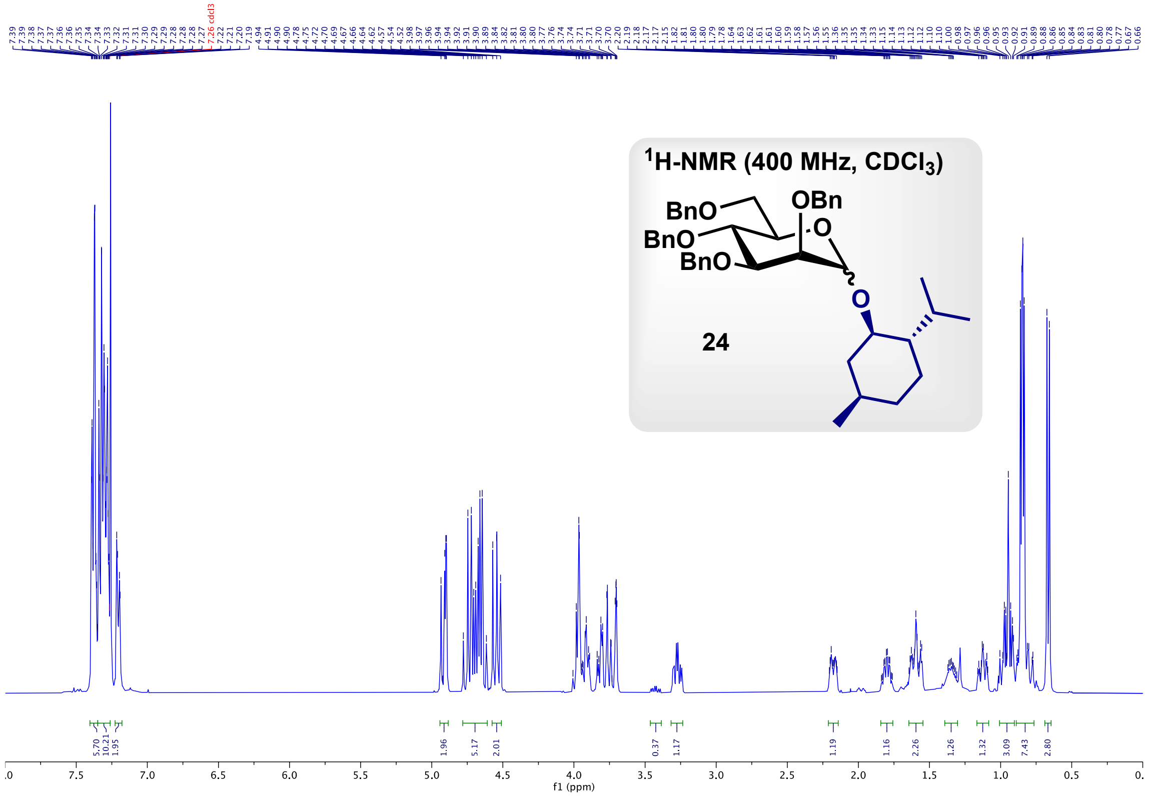
1H-NMR (400 MHz, CDCl3)
OBn
BnO
BnO
BnO
O
O
24
f1 (ppm)

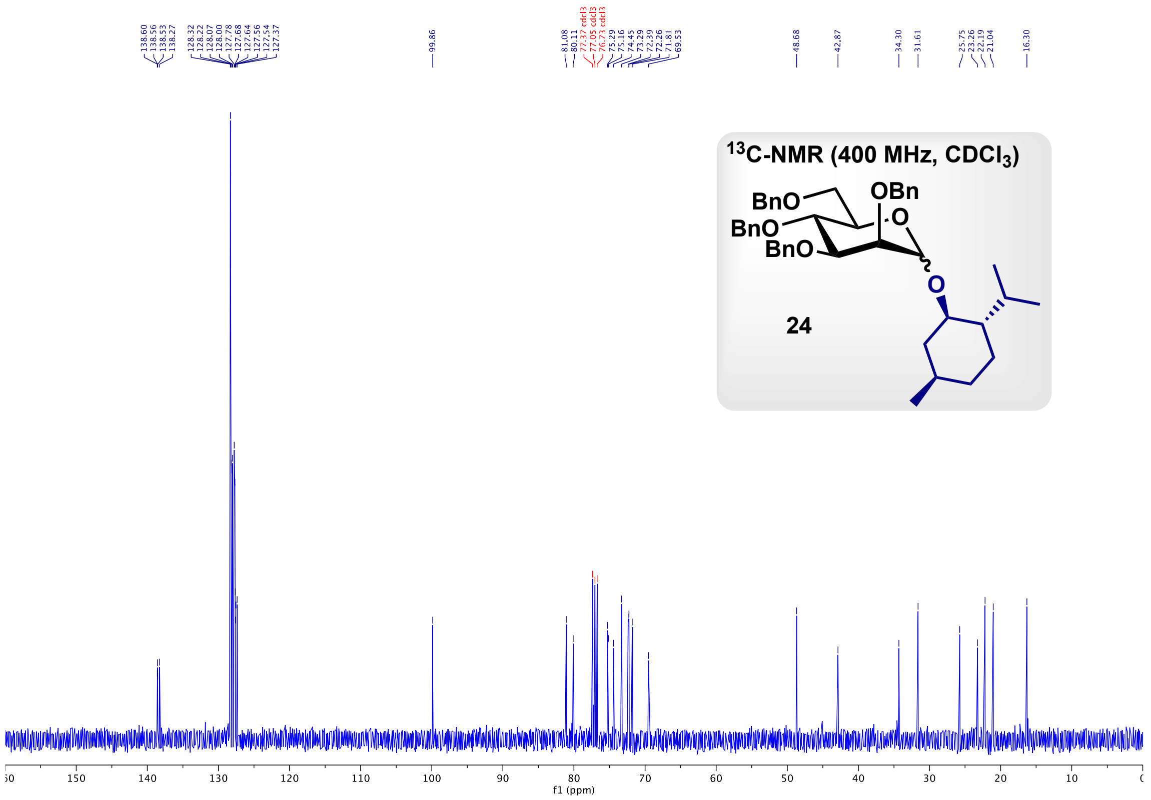
13C-NMR (400 MHz, CDCl3)
OBn
BnO
BnO
BnO
O
O
24
f1 (ppm)

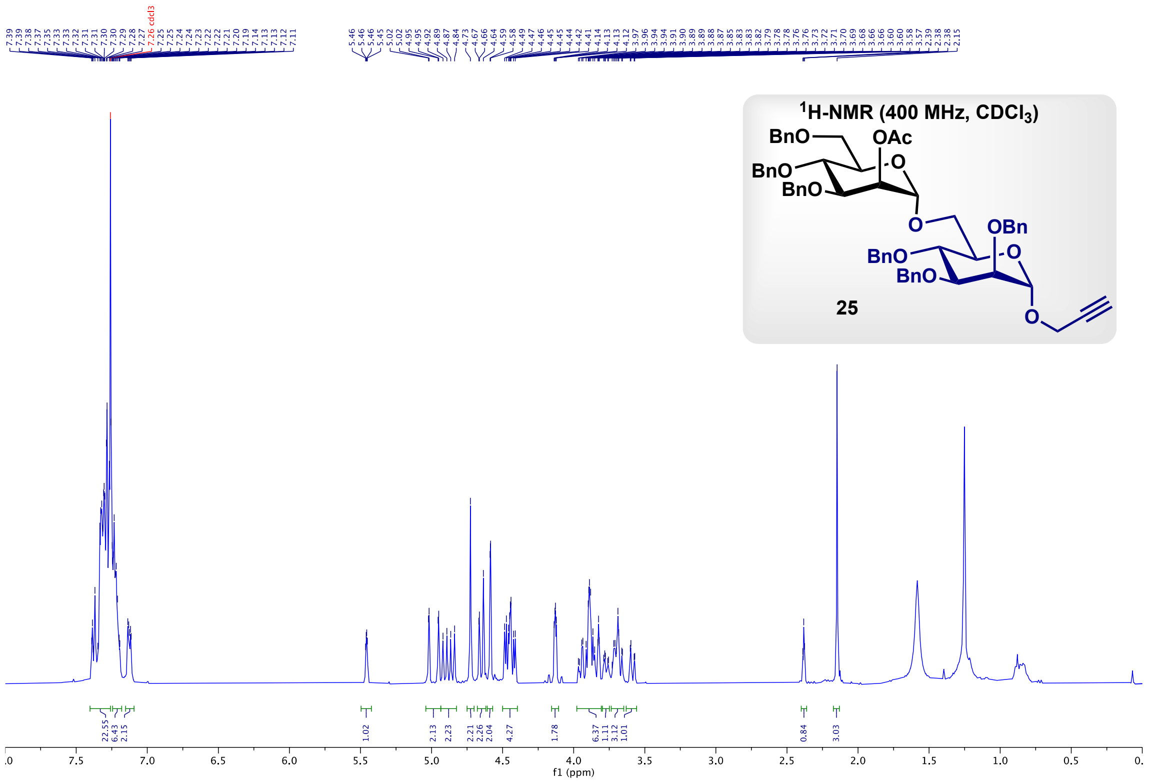

1H-NMR (400 MHz, CDCl3)
BnO
OAc
O
BnO
BnO
O
OBn
BnO
O
BnO
25
O
f1 (ppm)

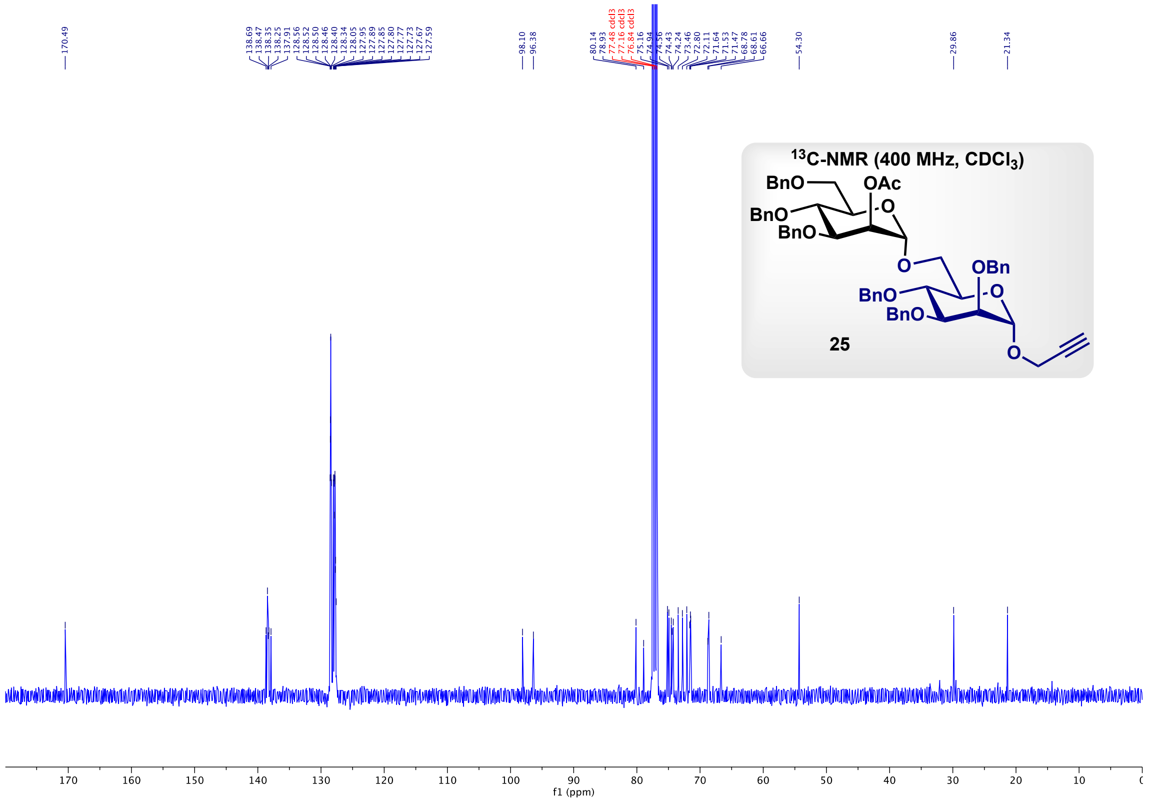

13C-NMR (400 MHz, CDCl3)
BnO
OAc
O
BnO
BnO
O
OBn
BnO
O
BnO
25
O
f1 (ppm)

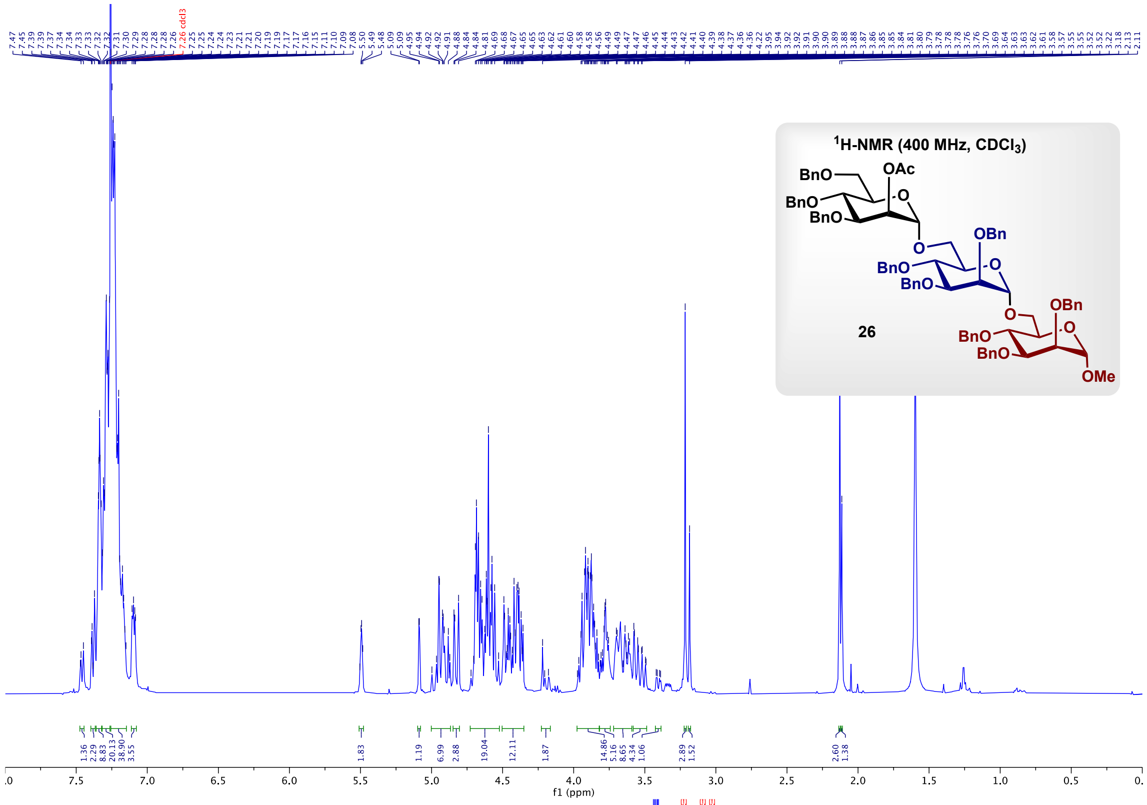
1H-NMR (400 MHz, CDCl3)
BnO
OAc
BnO
BnO
O
O
OBn
BnO
BnO
O
OBn
BnO
BnO
OMe
26
f1 (ppm)

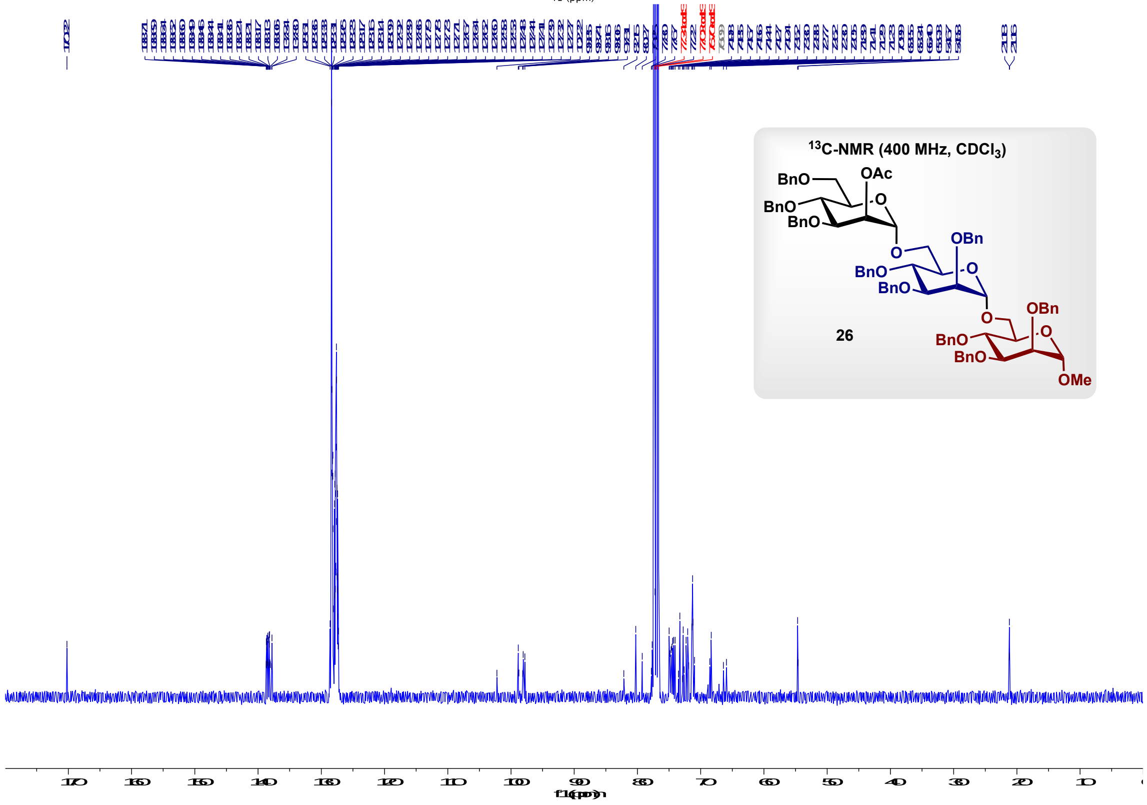
13C-NMR (400 MHz, CDCl3)
BnO
OAc
BnO
BnO
O
O
OBn
BnO
BnO
O
OBn
BnO
BnO
OMe
26

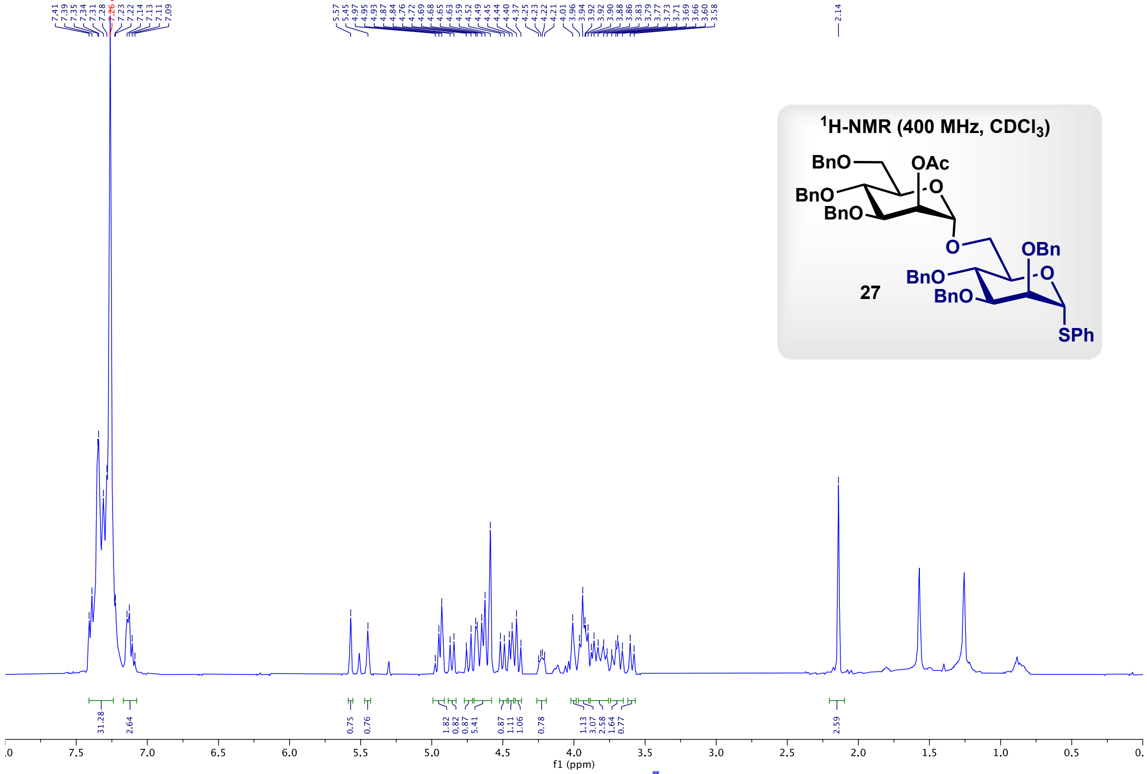

^{1}H-NMR (400 MHz, CDCl$_3$)
BnO
OAc
O
BnO
BnO
O
OBn
O
BnO
BnO
27
SPh
f1 (ppm)

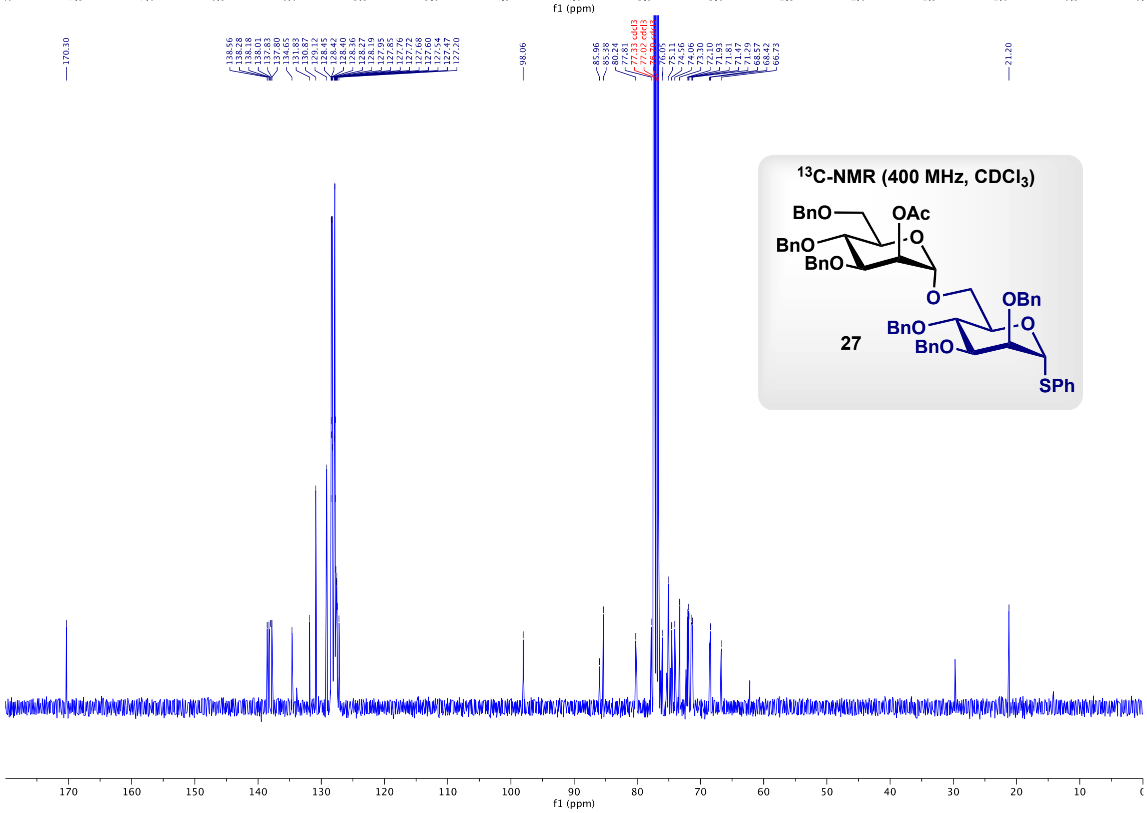

^{13}C-NMR (400 MHz, CDCl$_3$)
BnO
OAc
O
BnO
BnO
O
OBn
O
BnO
BnO
27
SPh
f1 (ppm)
